# Pharmacomodulation of the Redox-Active Lead Plasmodione: Synthesis of Substituted 2-Benzylnaphthoquinone Derivatives, Antiplasmodial Activities, and Physicochemical Properties

**DOI:** 10.3390/ijms26052114

**Published:** 2025-02-27

**Authors:** Armin Presser, Gregor Blaser, Eva-Maria Pferschy-Wenzig, Marcel Kaiser, Pascal Mäser, Wolfgang Schuehly

**Affiliations:** 1 Institute of Pharmaceutical Sciences, Pharmaceutical Chemistry, University of Graz, Schubertstrasse 1, 8010 Graz, Austria; gregor.blaser@gmx.at; 2 Institute of Pharmaceutical Sciences, Pharmacognosy, University of Graz, Beethovenstrasse 8, 8010 Graz, Austria; eva-maria.wenzig@uni-graz.at (E.-M.P.-W.); wolfgang.schuehly@uni-graz.at (W.S.); 3 Swiss Tropical and Public Health Institute, Kreuzstrasse 2, 4123 Allschwil, Switzerland or marcel.kaiser@unibas.ch (M.K.); or pascal.maeser@unibas.ch (P.M.); 4 Faculty of Philosophy and Natural Sciences, University of Basel, Petersplatz 1, 4003 Basel, Switzerland

**Keywords:** antiprotozoal activity, plasmodione, Kochi–Anderson reaction, physicochemical parameters, ligand efficiency indices

## Abstract

Malaria remains a major global health problem that has been exacerbated by the impact of the COVID-19 pandemic on health systems. To combat this, the World Health Organization (WHO) has set a target of driving forward research into innovative treatment methods such as new drugs and vaccines. Quinones, particularly 1,4-naphthoquinones, have been identified as promising candidates for the development of antiprotozoal drugs. Herein, we report several methods for the preparation of 2-benzyl-1,4-naphthoquinones. In particular, the silver-catalyzed Kochi–Anderson radical decarboxylation is well suited for the preparation of these compounds. The antiprotozoal activity of all synthesized compounds was evaluated against *Plasmodium falciparum* NF54 and *Trypanosoma brucei rhodesiense* STIB900. Cytotoxicity towards L6 cells was also determined, and the respective selectivity indices (SI) were calculated. The synthesized compounds exhibited good antiplasmodial activity against the *P. falciparum* (NF54) strain, particularly (2-fluoro-5-trifluoromethylbenzyl)-menadione **2e**, which showed strong efficacy and high selectivity (IC_50_ = 0.006 µM, SI = 7495). In addition, these compounds also displayed favorable physicochemical properties, suggesting that the benzylnaphthoquinone scaffold may be a viable option for new antiplasmodial drugs.

## 1. Introduction

Malaria, one of the world’s most malignant diseases, continues to be a major global health problem, especially in tropical and subtropical regions. In 2022, the World Health Organization (WHO) reported 249 million cases of malaria worldwide that caused an estimated 608,000 deaths in 85 countries [1]. This number demonstrates a significant worsening of the malaria situation compared to 2019 as a consequence of the global COVID-19 pandemic, which has resulted in strained health systems and diverted resources. In response to the ongoing challenges, WHO has updated its malaria strategy and its aims at reducing malaria cases and deaths by 90% by 2030 [2]. This ambitious goal may only be achieved through a renewed commitment to innovative strategies, e.g., effective malaria vaccines [3] and the development of new antimalarial drugs [4].

**Figure 1:**
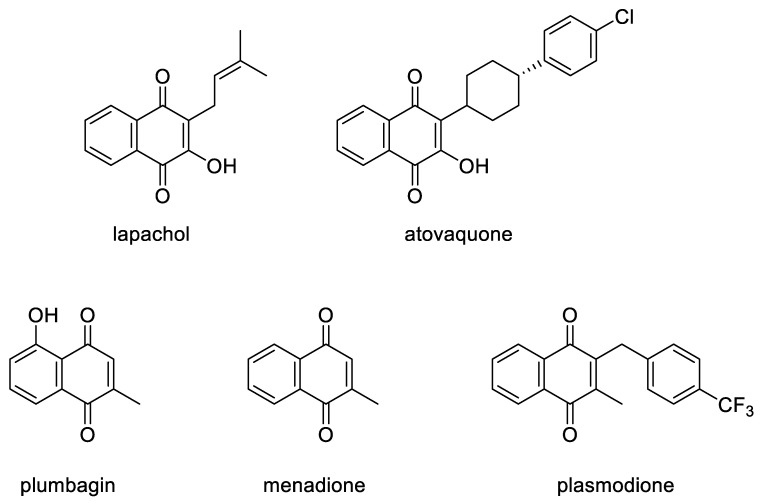
1,4-Naphthoquinones with remarkable biological activity.

Quinones have recently regained increasing interest in the field of medicinal chemistry, particularly in the discovery of new drugs for the treatment of neglected tropical parasitic diseases [5,6,7,8]. The quinone scaffold is found in a variety of secondary plant metabolites, e.g., lapachol or plumbagin (Figure 1), with a broad spectrum of biological activities [9,10,11,12,13]. Among the various quinone types, the 1,4-naphthoquinone (NQ) scaffold has been identified as a promising pharmacophore for further drug development due to its activity against various apicomplexan parasites, above all, the malaria pathogens of the subgroup *Plasmodium* [13,14,15,16,17].

In this context, a redox-active compound called plasmodione (**2b**), a member of the benzylmenadione family with a unique mechanism of action, has shown particularly promising profiles [18,19,20,21,22]. Plasmodione kills parasites as efficiently as the currently most efficient available drug, i.e., artemisinin, besides, it is effective against many drug-resistant parasites and shows a low potential for the development of genetic resistance [21]. Furthermore, despite its chemical similarity to the common antimalarial drug atovaquone, plasmodione does not target the same mitochondrial protein and is therefore effective against atovaquone-resistant strains [23].

In the present study, we synthesized 27 plasmodione-like derivatives with a benzylnaphthoquinone core to investigate their antimalarial potential in comparison to the original lead plasmodione. To assess the druggability of our synthesized compounds, we also analyzed the safety profile, various physicochemical parameters, and ligand efficiency metrics.

## 2. Results and Discussion

### 2.1. Synthetic Chemistry

There are several chemical routes to introduce a benzyl moiety into the 1,4-naphthoquinone scaffold [24,25], including a silver-catalyzed decarboxylative cross-coupling of the 1,4-naphthoquinone core with carboxylic acids [20,26], an iron-catalyzed or light-induced radical benzylation of quinones starting from benzyl bromides [27,28], or optionally via a four-step route based on tetralone [26,29].

**Scheme 1:**
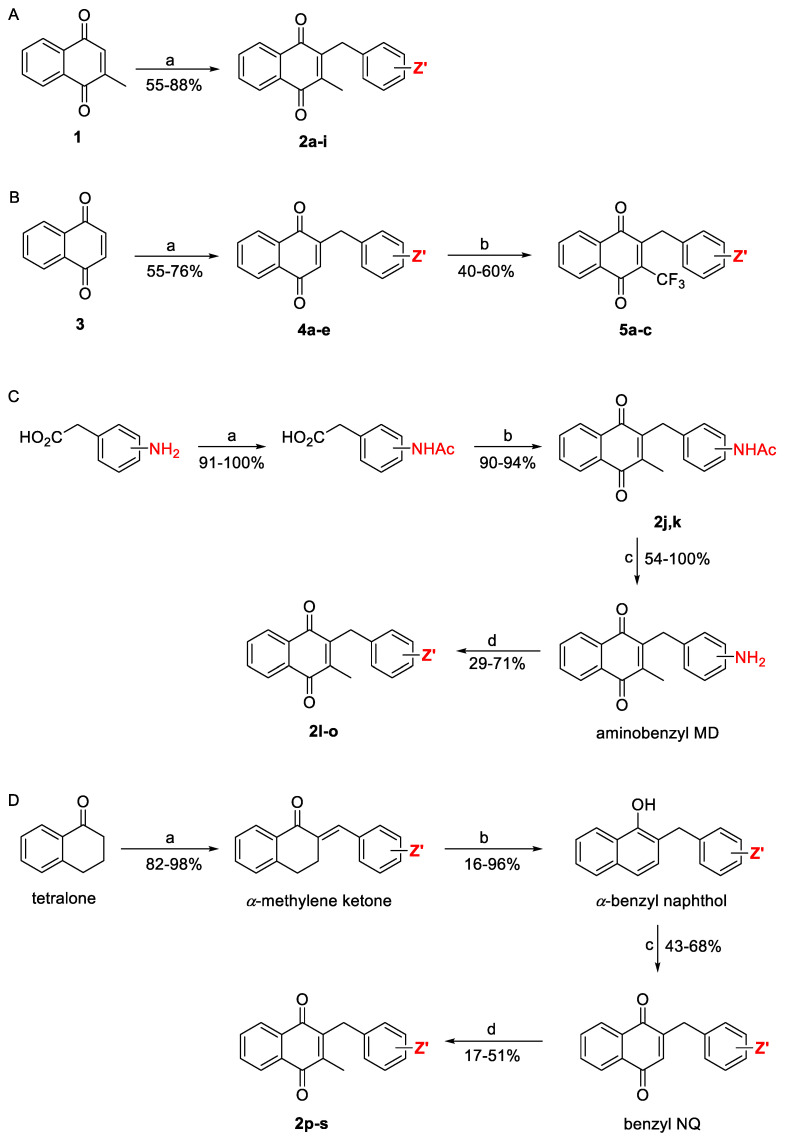
Synthesis of benzyl-substituted naphthoquinone and menadione derivatives via Kochi–Anderson reaction (**A**–**C**) or an alternative tetralone route (**D**). Reagents and conditions: (**A**): (a) phenylacetic acid derivative (1.4 equiv.), AgNO_3_ (0.35 equiv.), (NH_4_)_2_S_2_O_8_ (1.3 equiv.), CH_3_CN/H_2_O, 85 °C, 1–4 h. (**B**): (a) phenylacetic acid derivative (1.4 equiv.), AgNO_3_ (0.1 equiv.), (NH_4_)_2_S_2_O_8_ (2 equiv.), CH_3_CN/CH_2_Cl_2_, 80 °C, 1.5–2.5 h; (b). CuI (0.5 equiv.), B_2_pin_2_ (0.01 equiv.), Togni reagent (2 equiv.), CHCl_3_, 85 °C, 20–26 h. (**C**): (a) AcOH, mw, 150 °C, 40 min; (b) acetaminophenylacetic acid (2 equiv.), menadione (1 equiv.), AgNO_3_ (0.35 equiv.), (NH_4_)_2_S_2_O_8_ (1.3 equiv.), CH_3_CN/H_2_O, 85 °C, 1.5 h; (c) MeOH/HCl *conc*., mw, 150 °C, 30 min; (d) carboxylic acid (1 equiv.), DCC (1.2 equiv.), DMF, rt, 3–4 h. (**D**): (a) benzaldehyde derivative (1.1 equiv.), KOH/EtOH, rt, 2.5–4 h; (b) RhCl_3_ (0.1 equiv.), EtOH, 85 °C, 24 h; (c) PIDA (2.1 equiv.), CH_3_CN/H_2_O, −5 °C → rt, 1.5 h; (d) AcOH (5 equiv.), AgNO_3_ (0.35 equiv.), (NH_4_)_2_S_2_O_8_ (1.3 equiv.), CH_3_CN/H_2_O, 85 °C, 3 h.

The silver-catalyzed radical decarboxylation reaction (Kochi–Anderson reaction) [30] provides a powerful tool for fast and easy access to 2-methyl-1,4-naphthoquinone (menadione, **1**) derivatives in only one step. By using this method, we first synthesized a series of menadione (MD) derivatives (**2a**–**i**) from commercial phenylacetic acids in good to moderate yields (55–88%, Scheme 1, route A). The reaction was compatible with a wide range of substituents, such as halogens, trifluoromethoxy, or nitro groups. For the preparation of benzylated desmethyl derivatives (**4a**–**e**), the conditions had to be slightly modified to avoid the formation of disubstituted products (Scheme 1, route B).

Trifluoromenadione has also been proposed as a promising core for antimalarial drugs [31]. Therefore, a series of benzylated trifluoromenadiones (**5a**–**c**) were prepared from the previously obtained naphthoquinones **4a**–**c** via a direct copper-catalyzed C-H trifluoromethylation [32,33,34]. Interestingly, the reaction was only successful when a different approach to the published procedure was applied, such as the mandatory addition of bis(pinacolato)diboron (B_2_pin_2_) and the presence of CuI as a catalyst. Under these optimized conditions (which are described in detail in Section 3), the trifluoromenadiones **5a**–**c** could be obtained in satisfactory yields.

Urgin and co-workers [35] investigated the antimalarial potential of N-substituted benzylmenadiones and found unexpectedly high antimalarial activity in their N-tert-butoxycarbonyl-protected intermediates. The same authors also report that a Kochi–Anderson benzylation of unprotected amino phenylacetic acids is not successful and leads mainly to degradation of the starting materials. We have taken these observations as an incentive to investigate the antimalarial potential of a few (mainly fluorinated) amidobenzylmenadiones wherein a microwave-assisted N-acetyl-protection strategy was used to achieve this goal (Scheme 1, route C). In this way, the acetamidobenzyl derivatives **2j**,**k** were obtained excellently and the fluorinated derivatives **2l**–**o** in good to moderate yields.

Due to the limited choice of commercially available phenylacetic acids, we have taken an alternative 4-step route, starting from commercial tetralone via the corresponding α-methylene ketones and α-benzylnaphthols to the final benzylmenadione derivatives (Scheme 1, route D). In this alternative scenario, the final benzylic chain is already introduced in the first step by coupling with various benzaldehydes [26,29]. However, the tedious synthetic route in combination with the rather poor stability of the methyl radical in the final Kochi–Anderson step has led to overall not impressive yields for the benzylmenadiones **2p**–**s**. Nevertheless, sufficient material for biological evaluations could be obtained. Overall yields of all synthesized naphthoquinone and menadione derivatives are presented in Table 1.

### 2.2. Biological Evaluation

The synthesized benzylmenadione and naphthoquinone derivatives were tested in vitro for their antiprotozoal activity against *P. falciparum* (NF54) erythrocytic stages and *T. brucei rhodesiense* (STIB900) bloodstream forms. Cytotoxicity was assessed towards rat L6 skeletal muscle cells to calculate a selectivity index for each parasite (SI = IC_50(L6)_/IC_50(parasite)_).

According to the recommended hit-to-lead identification criteria [36,37,38], most of the derivatives showed good to very good antiplasmodial activity against NF54 (Table 2), with the (2-fluoro-5-trifluoromethylbenzyl) menadione **2e** exhibiting the strongest activity (IC_50_ = 0.006 μM) along with the highest selectivity (SI = 7495). Trypanocidal activity was also observed (strongest with benzyl-NQ **4a**), but generally much lower.

Toxicity prediction was performed using the online platform ProTox-3.0 [39], a web-based service that allows prediction of toxicity using different machine-learning models. These calculations predicted low acute oral toxicity for our compounds with LD_50_ values not lower than 500 mg/kg. The antiplasmodial top performer **2e** together with **2f** and **4e** achieved herein the best result with a calculated LD_50_ of 2000 mg/kg. Therefore, substituted 2-benzylnaphthoquinone derivatives may have a high potential for the development of new antiprotozoal drugs [40].

### 2.3. Physicochemical Investigations

Calculated physicochemical parameters play a pivotal role in drug development, as they help to assess the pharmacological profiles of candidates for new drugs in terms of pharmacokinetics, efficacy, and safety [41,42,43]. The fundamental work of Lipinski et al. [44,45], who established the ‘rule of five’, provides a framework for evaluating the drug suitability of compounds based on their physicochemical properties and emphasizes the importance of these parameters in the early stages of drug discovery. For this reason, all tested compounds were subjected to a detailed drug suitability evaluation, and a number of physicochemical parameters were calculated. All synthesized compounds have relatively low molecular weights (from 248 to 402 g mol^−1^), and the log*D* values (at pH 7.4) are also suitable [38]. Furthermore, they fulfill the drug-likeness criteria proposed by Lipinski et al. [44,45] and Veber et al. [46].

Efficacy and physicochemical properties must be given equal consideration in drug development [47,48,49,50]. In recent years, ligand efficiency indices and multi-parameter scores such as ligand efficiency metrics (LE), lipophilic ligand efficiency (LLE), and binding efficiency indices (BEI) have proven to be useful tools in lead discovery and optimization, thus providing a benchmark for assessing the pharmacological value of small molecules [51,52,53,54,55].

However, ligand efficiency (LE) is not the best choice to find a drug candidate across a wide range of ligand sizes. In certain cases, size-independent metrics such as SILE (size-independent ligand efficiency) should be preferred [51,56,57]. While Lipinski’s Rule of 5 (Ro5) describes the chemical space with the highest probability of good oral absorption, there is now increasing interest in new therapeutic agents that may not strictly follow this rule (bRo5) [43,49,58,59]. This requires more advanced tools such as the AbbVie Multi-Parameter Score (Abb-MPS) [60] or the Property Forecast Index (PFI) [61].

We have calculated the main physicochemical parameters, ligand efficiency indices and multi-parameter scores of our tested compounds and have listed them in Tables S1 and S2 in the Supplementary Materials.

### 2.4. Structure–Activity Relationships (SAR) of the Antiplasmodial Activity

Nearly all the compounds in this study met AB-MPS [60], PFI [61], CNS multiparameter optimization(CNS-MPO) [62,63], and Quantitative estimate of drug-likeness (QED) [64,65] recommendations.

Most remarkable was the strong correlation of some of the calculated ligand efficiency metrics (especially size-independent ligand efficiency (SILE)) with the obtained antiplasmodial activity of our synthesized compounds (e.g., SILE_P.f_., ρ = −0.95; LLE_P.f_., ρ = −0.75). The Spearman correlation (ρ) used for this purpose is robust against outliers, as it evaluates the relationship between two variables without assuming a linear association or requiring normally distributed data. Interestingly, other size-independent ligand efficiency metrics such as ligand efficiency lipophilic price (LELP) [51] or Astex lipophilic ligand efficiency (LLE_AT_) [66] showed no notable correlation with the efficacy of our substances.

**Figure 2:**
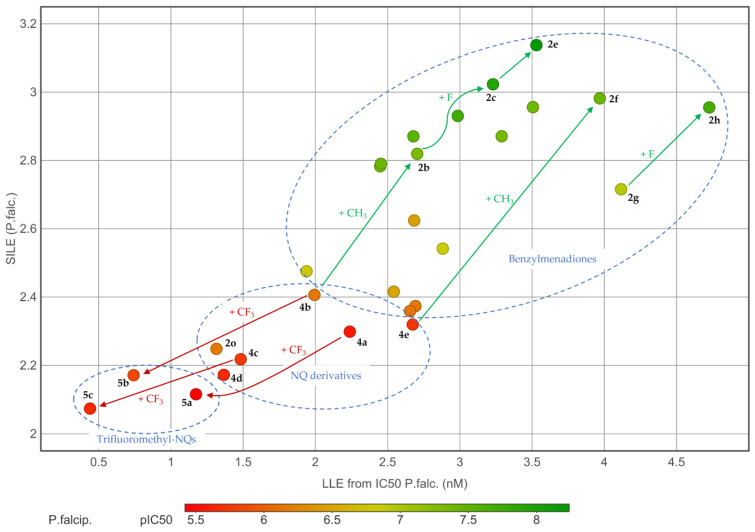
Scatterplot of lipophilic ligand efficiency (LLE) vs. size-independent ligand efficiency (SILE) for the synthesized compounds including some optimization trajectories; the colors are assigned according to their respective pIC_50_.

Closer examination of LLE and SILE values of our benzylnaphthoquinone derivatives revealed a clear clustering of compounds with similar structural elements and their respective antiplasmodial activities (Figure 2 and Figure S1 in the Supplementary Materials).

The 3-(trifluoromethyl)-NQs (**5a**–**c**) located in the lower left corner of the plot show the least exciting values of LLE < 1.2, SILE < 2.2 and pIC_50_ ≤ 5.8. The NQ derivatives (**4a**–**e**) also showed a rather low level of activity against *P. falciparum* and SILE values ≤ 2.4. Among the menadione derivatives, only the 4,4,4-trifluorobutanamidobenzyl menadione **2o** displayed comparably poor levels (with a pIC_50_ of 6.2). All other benzylmenadione derivatives, especially the polyfluorinated ones, are highly effective (pIC_50_ in most cases ≥ 7) and lie in the upper right quadrant.

The trajectories of selected compounds in LLE and SILE space are also visualized in Figure 2. The concept of trajectory mapping provides an excellent strategic framework to evaluate the optimization process in drug development [67]. For example, the introduction of a methyl group into the NQ backbone (**4b**→**2b**, **4e**→**2f**) leads to a significant increase in potency, as indicated by a clear shift towards the north-eastern part of the SILE-LLE region. Inserting a CF_3_ group, on the other hand (**4a**→**5a**, **4b**→**5b**, **4c**→**5c**), tends to reduce efficiency (shift to the bottom left). Furthermore, it can easily be deduced how the efficacy of the model substance plasmodione (**2b**) can be enhanced by further fluorination and subsequent rearrangement of the fluorinated building blocks (**2b**→**2c**→**2e**). These results underline the benefit of the SILE vs. LLE plot as very useful for assessing the efficacy of different functional groups and residues in a drug development process.

Abad-Zapatero et al. describe an equally useful mathematical approach that integrates different ligand efficiency indices (LEIs) and defines binding affinity as a function of ligand size (usually expressed as binding affinity per non-hydrogen atom or per molecular weight) and polarity [55,68]. The application of LEIs goes beyond simple efficacy assessment; they are an important tool for Cartesian mapping of chemical–biological space, a concept referred to as AtlasCBS [69]. This mapping allows the identification and visualization of promising drug candidates and the comparison of different ligands for various targets. The pair of variables (NSEI, *n*BEI) is particularly useful to determine the ’direction’ of the optimization path, which is specified by the corresponding framework. In the NSEI-*n*BEI plot, the compounds appear along a series of lines with slopes equal to NPOL (number of polar atoms). The most efficient compounds in terms of size and polarity are found in the north-eastern region of the plane and are therefore considered the more promising candidates for further drug development.

**Figure 3:**
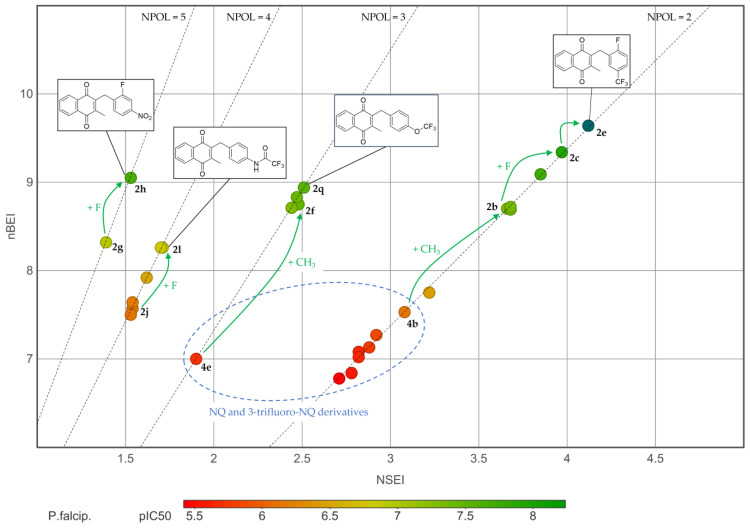
Application of the AtlasCBS concept using a schematic NSEI-*n*BEI plane analysis of the synthesized benzyl-naphthoquinones including optimization trajectories; the colors are assigned according to their respective pIC_50_.

The application of AtlasCBS to our synthesized compounds gave a very similar result to the LLE/SILE scatterplot (Figure 2). Again, the potent polyfluorinated menadione derivatives were located in the preferred northeastern part of the NSEI-*n*BEI plane, with **2e** leading the way, while the less potent NQ and 3-trifluoro-NQ derivatives tended to be found in the lower left region (Figure 3 and Figure S2 in the Supplementary Materials).

The concept of trajectory mapping can also be applied very well to the NSEI/nBEI plot; the increase in efficiency due to the introduction of a methyl group in the NQ backbone (**4b**→**2b**, **4e**→**2f**), as well as the introduction of fluorine atoms (**2g**→**2h**; **2j**→**2l**; **2b**→**2c**→**2e**) are clearly reflected by a significant shift towards the preferred northeastern part of the NSEI-*n*BEI plane.

**Figure 4:**
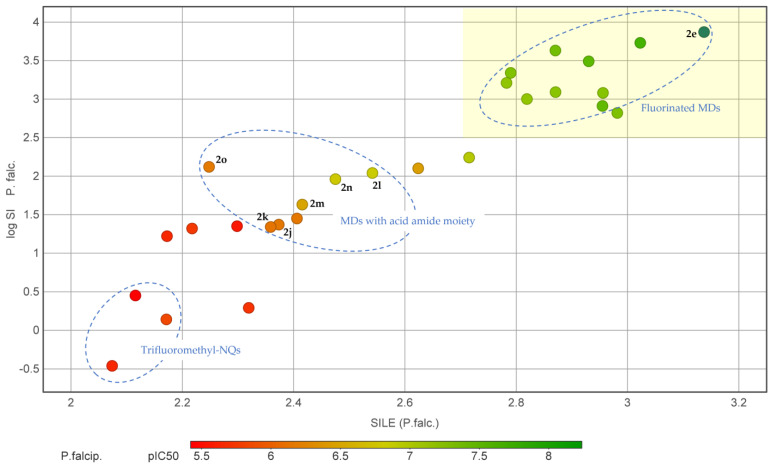
Plot of SILE (*P. falciparum*) data obtained in this study (x) vs. calculated log SI (*P. falciparum*) (y); the colors are assigned according to their respective pIC_50_. Substances that lie in the yellow shaded area combined high selectivity with acceptable size-independent ligand efficiency values.

The excellent potential of (poly)fluorinated menadiones for the development of new antiplasmodial agents is also reflected in the SILE/log SI correlation plot (Figure 4 and Figure S3 in the Supplementary Materials), where most of these compounds showed more than 300-fold selectivity (log SI > 2.5) between the IC_50_ for the rat L6 skeletal cell line and the IC_50_ for *Plasmodium falc.*, as well as adequate ligand efficiency indices. Compounds with SI > ~300 and pIC_50_ values greater than 7 (corresponding to activity < 0.1 mM) combine high activity with acceptable selectivity against an L6 cell line and are thus considered promising drug candidates for further studies [36,37,38]. However, the SILE/log SI scatterplot also proves that (even fluorinated) menadiones with an acid amide moiety **2j**–**o** do not lead to acceptable results.

## 3. Materials and Methods

### 3.1. Chemistry

#### 3.1.1. General Information

All reagents and solvents were obtained from Merck and Fluorochem Ltd. (Pune, India) Moisture-sensitive reactions were performed under an inert argon atmosphere. Each reaction was monitored by TLC on Merck TLC plates (silica gel 60 F254 0.2 mm, 200 × 200 mm) and detected at 254 nm. All reaction products were purified by flash column chromatography on silica gel 60 (Merck, Darmstadt, Germany, 70–230 mesh, pore diameter 60 Å) unless otherwise stated. Purity and homogeneity of the final compounds were checked by TLC and high-resolution mass spectrometry. Melting points were determined using a digital melting point meter (Electrothermal IA 9200, Thermo Fisher Scientific, Birmingham, UK). Microwave-assisted reactions were carried out in a CEM Discover/Explorer system in sealed 10 cm^3^ standard vessels with temperature control.

Accurate structural elucidation was confirmed by 1D and 2D NMR spectroscopy on a Bruker Avance Neo 400 MHz instrument (at 298 K) using 5 mm tubes. Chemical shifts were expressed in δ (ppm) using either tetramethylsilane (TMS) or the ^13^C signal of the solvents (CDCl_3_ δ 77.04 ppm, DMSO-*d*_6_ δ 39.45 ppm) as the internal standard. ^1^H and ^13^C resonances were numbered according to the formulae (see Supplementary Materials); signals marked with an asterisk are interchangeable.

ESI and APCI mass spectra were acquired by analyzing sample solutions on an Ultimate 3000 HPLC with a Q Exactive™ Hybrid Quadrupole-Orbitrap™ mass spectrometer equipped with a heated ESI II source or an APCI source (Thermo Fisher Scientific, Birmingham, UK) in positive or negative ionization mode.

#### 3.1.2. General Synthetic Procedure for Benzylmenadiones **2a**–**i** (Route A)

Menadione (1.0 mmol) was added to a stirred solution of the respective phenylacetic acid derivative (1.4 equiv.) in CH_3_CN (9 mL) and H_2_O (3 mL) and heated to 85 °C. AgNO_3_ (0.35 equiv.) was added first and then (NH_4_)_2_S_2_O_8_ (1.3 equiv.) dissolved in 4 mL CH_3_CN/H_2_O (3:1) dropwise over a period of 5 min. Stirring at 85 °C was continued until TLC showed complete consumption of the starting material (1–4 h). After cooling to ambient temperature, the mixture was extracted three times with CH_2_Cl_2_ (20 mL). The combined organic layers were washed with H_2_O, dried over Na_2_SO_4_, and concentrated in vacuo to give a residue, which was purified by flash chromatography as detailed below.

*2-Benzyl-3-methyl-1,4-naphthoquinone* (**2a**). Compound **2a** was obtained after stirring for 1 h, then purified by flash chromatography using toluene/cyclohexane (15:1). Yellow solid; yield: 88%; *R*_f_ = 0.50 (toluene:cyclohexane = 15:1); m.p.: 104–106 °C (lit [28] m.p. 105–106 °C). The spectroscopic data were found to be identical to the ones described in [28]. Although **2a** represents an already known compound, to our knowledge the complete assignment of the NMR signals has not been published so far: ^1^H NMR (400 MHz, CDCl_3_) δ = 8.09 (m, 2H, H-8, H-5), 7.69 (m, 2H, H-6, H-7), 7.26 (m, 4H, H-2′, H-3′, H-5′, H-6′), 7.19 (m, 1H, H-4′), 4.03 (s, 2H, H-9), 2.25 (s, 3H, H-10) ppm; ^13^C NMR (100 MHz, CDCl_3_): δ = 185.4 (C-4), 184.7 (C-1), 145.3 (C-3), 144.4 (C-2), 138.0 (C-1′), 133.5 (C-6, C-7), 132.1 (C-4a), 132.0 (C-8a), 128.6 (C-2′, C-3′, C-5′, C-6′), 126.5 (C-5), 126.4 (C-4′), 126.3 (C-8), 32.4 (C-9), 13.3 (C-10) ppm.

*2-Methyl-3-{[4-(trifluoromethyl)phenyl]methyl}-1,4-naphthoquinone* (**2b**). Compound **2b** was obtained after stirring for 1.5 h, then purified by flash chromatography using toluene/cyclohexane (15:1). Yellow solid; yield: 83%; *R*_f_ = 0.42 (toluene:cyclohexane = 15:1); m.p.: 70–71 °C (lit [28] m.p. 70–71 °C). The spectroscopic data were found to be identical to the ones described in [28]. Although **2b** represents an already known compound, to our knowledge the complete assignment of the NMR signals has not been published so far: ^1^H NMR (400 MHz, DMSO-*d*_6_) δ = 7.98 (m, 2H, H-5, H-8), 7.82 (m, 2H, H-6, H-7), 7.60 (d, *J* = 7.8 Hz, 2H, H-3′, H-5′), 7.45 (d, *J* = 7.8 Hz, 2H, H-2′, H-6′), 4.06 (s, 2H, H-9), 2.14 (s, 3H, H-10) ppm; ^13^C NMR (100 MHz, DMSO-*d*_6_) δ = 184.4 (C-4), 183.9 (C-1), 144.8* (C-2), 143.4* (C-3), 143.2 (C-1′), 133.9* (C-6), 133.8* (C-7), 131.6* (C-4a), 131.3* (C-8a), 129.2 (C-2′, C-6′), 126.9 (q, ^2^*J*_C,F_ = 31.8 Hz, C-4′), 125.9* (C-5), 125.8* (C-8), 125.2 (q, ^3^*J*_C,F_ = 3.9 Hz, C-3′, C-5′), 124.2 (q, ^1^*J*_C,F_ = 271.9 Hz, CF_3_), 31.6 (C-9), 13.0 (C-10) ppm.

*2-{[2-Fluoro-4-(trifluoromethyl)phenyl]methyl}-3-methyl-1,4-naphthoquinone *(**2c**). Compound **2c** was obtained after stirring for 3 h, then purified by flash chromatography using toluene/cyclohexane (15:1). Yellow solid; yield: 69%; *R*_f_ = 0.48 (toluene:cyclohexane = 15:1); m.p.: 86–87 °C. ^1^H NMR (400 MHz, CDCl_3_) δ = 8.10 (m, 2H, H-5, H-8), 7.72 (m, 2H, H-6, H-7), 7.31 (m, 3H, H-3′, H-5′, H-6′), 4.09 (s, 2H, H-9), 2.23 (s, 3H, H-10) ppm; ^13^C NMR (100 MHz, CDCl_3_) δ = 184.9 (C-4), 184.3 (C-1), 160.4 (d, ^1^*J*_C,F_ = 248.2 Hz, C-2′), 145.6 (C-3), 143.2 (C-2), 133.7 (C-6, C-7), 132.1 (C-4a), 131.9 (C-8a), 131.1 (d, ^3^*J*_C,F_ = 4.4 Hz, C-6′), 130.7 (qd, ^2^*J*_C,F_ = 33.4, ^3^*J*_C,F_ = 8.0 Hz, C-4′), 129.2 (d, ^2^*J*_C,F_ = 15.5 Hz, C-1′), 126.5 (C-5, C-8), 123.7 (qd, ^1^*J*_C,F_ = 272.1, ^4^*J*_C,F_ = 2.5 Hz, CF_3_), 121.1 (C-5′), 112.9 (dq, ^2^*J*_C,F_ = 25.7, ^3^*J*_C,F_ = 3.8 Hz, C-3′), 25.7 (C-9), 13.1 (C-10) ppm; HRMS (APCI) calcd. for C_19_H_12_F_4_O_2_ [M]^−^ = 348.0773; found: 348.0783.

*2-{[4-Fluoro-2-(trifluoromethyl)phenyl]methyl}-3-methyl-1,4-naphthoquinone* (**2d**). Compound **2d** was obtained after stirring for 3 h, then purified by flash chromatography using toluene/cyclohexane (15:1). Yellow solid; yield: 66%; *R*_f_ = 0.50 (toluene:cyclohexane = 15:1); m.p.: 86–87 °C. ^1^H NMR (400 MHz, DMSO-*d*_6_) δ = 8.06 (m, 1H, H-5), 7.98 (m, 1H, H-8), 7.85 (m, 2H, H-6, H-7), 7.64 (dd, *J* = 9.3, 2.7 Hz, 1H, H-3′), 7.33 (td, *J* = 8.4, 2.7 Hz, 1H, H-5′), 7.28 (m, 1H, H-6′), 4.07 (s, 2H, H-9), 2.05 (s, 3H, H-10) ppm; ^13^C NMR (100 MHz, DMSO-*d*_6_) δ = 184.3 (C-4), 183.8 (C-1), 160.1 (d, ^1^*J*_C,F_ = 244.8 Hz, C-4′), 146.1 (C-3), 142.3 (C-2), 133.9 (C-6), 133.8 (C-7), 132.5 (C-1′), 131.9 (C-4a), 131.5 (C-8a), 131.1 (d, ^3^*J*_C,F_ = 8.0 Hz, C-6′), 128.5 (qd, ^2^*J*_C,F_ = 30.6, ^3^*J*_C,F_ = 7.6 Hz, C-2′), 125.9 (C-5, C-8), 123.6 (qd, ^1^*J*_C,F_ = 274.3, ^4^*J*_C,F_ = 2.8 Hz, CF_3_), 119.4 (d, ^2^*J*_C,F_ = 20.3 Hz, C-5′), 113.7 (dq, ^2^*J*_C,F_ = 25.4, ^3^*J*_C,F_ = 6.0 Hz, C-3′), 27.8 (C-9), 12.8 (C-10) ppm; HRMS (APCI) calcd. for C_19_H_12_F_4_O_2_ [M]^−^ = 348.0773; found: 348.0784.

*2-{[2-Fluoro-5-(trifluoromethyl)phenyl]methyl}-3-methyl-1,4-naphthoquinone* (**2e**). Compound **2e** was obtained after stirring for 3.5 h, then purified by flash chromatography using toluene/cyclohexane (15:1). Yellow solid; yield: 63%; *R*_f_ = 0.44 (toluene:cyclohexane = 15:1); m.p.: 88–89 °C. ^1^H NMR (400 MHz, DMSO-*d*_6_) δ = 8.02 (m, 1H, H-5), 7.98 (m, 1H, H-8), 7.84 (m, 2H, H-6, H-7), 7.66 (m, 2H, H-4′, H-6′), 7.42 (t, *J* = 9.2 Hz, 1H, H-3′), 4.05 (s, 2H, H-9), 2.14 (s, 3H, H-10) ppm; ^13^C NMR (100 MHz, DMSO-*d*_6_) δ = 184.4 (C-4), 183.8 (C-1), 162.3 (dq, ^1^*J*_C,F_ = 250.4, 1.5 Hz, C-2′), 145.4* (C-3), 142.1* (C-2), 133.7 (C-6, C-7), 131.8* (C-4a), 131.5* (C-8a), 127.7 (C-6′), 127.1 (C-1′), 125.9 (C-4′), 125.8 (C-5, C-8), 125.3 (qd, ^2^*J*_C,F_ = 32.3, ^4^*J*_C,F_ = 3.3 Hz, C-5′), 123.7 (q, ^1^*J*_C,F_ = 271.1 Hz, CF_3_), 116.3 (d, ^2^*J*_C,F_ = 23.8 Hz, C-3′), 25.4 (C-9), 12.9 (C-10) ppm; HRMS (ESI) calcd. for C_19_H_11_F_4_O_2_ [M-H]^−^ = 347.0695; found: 347.0703.

*2-[(6-Chloropyridin-3-yl)methyl]-3-methyl-1,4-naphthoquinone* (**2f**). Compound **2f** was obtained after stirring for 3 h, then purified by flash chromatography using toluene/cyclohexane (15:1). Yellow solid; yield: 60%; *R*_f_ = 0.48 (toluene:cyclohexane = 15:1); m.p.: 159–160 °C. ^1^H NMR (400 MHz, DMSO-*d*_6_) δ = 8.35 (dd, *J* = 2.5, 0.8 Hz, 1H, H-2′), 8.00 (m, 2H, H-5, H-8), 7.84 (m, 2H, H-6, H-7), 7.71 (dd, *J* = 8.3, 2.6 Hz, 1H, H-4′), 7.40 (dd, *J* = 8.3, 0.7 Hz, 1H, H-5′), 4.00 (s, 2H, H-9), 2.18 (s, 3H, H-10) ppm; ^13^C NMR (100 MHz, DMSO-*d*_6_) δ = 184.4 (C-4), 184.0 (C-1), 149.7 (C-2′), 148.0 (C-6′), 145.0 (C-3), 142.9 (C-2), 139.6 (C-4′), 133.9* (C-7), 133.8* (C-6), 133.7 (C-3′), 131.7 (C-8a), 131.4 (C-4a), 125.9* (C-5), 125.8* (C-8), 124.0 (C-5′), 28.5 (C-9), 13.0 (C-10) ppm; HRMS (ESI) calcd. for C_17_H_13_ClNO_2_ [M+H]^+^ = 298.0635; found: 298.0627.

*2-Methyl-3-[(4-nitrophenyl)methyl]-1,4-naphthoquinone* (**2g**). Compound **2g** was obtained after stirring for 2.5 h, then purified by flash chromatography using toluene/cyclohexane (15:1). Yellow solid; yield: 66%; *R*_f_ = 0.40 (toluene:cyclohexane = 15:1); m.p.: 160–161 °C (lit [18] m.p. 156–157 °C). The spectroscopic data were found to be identical to the ones described in [18,28]. Although **2g** represents an already known compound, to our knowledge the complete assignment of the NMR signals has not been published so far: ^1^H NMR (400 MHz, DMSO-*d*_6_) δ = 8.13 (d, *J* = 8.8 Hz, 2H, H-2′, H-6′), 8.02 (m, 2H, H-5, H-8), 7.85 (m, 2H, H-6, H-7), 7.53 (d, *J* = 8.8 Hz, 2H, H-3′, H-5′), 4.13 (s, 2H, H-9), 2.16 (s, 3H, H-10) ppm; ^13^C NMR (100 MHz, DMSO-*d*_6_) δ = 184.5 (C-4), 183.9 (C-1), 146.6 (C-4′), 145.9 (C-1′), 145.2 (C-3), 143.1 (C-2), 133.9 (C-6, C-7), 131.7*(C-4a), 131.4*(C-8a), 129.5 (C-3′, C-5′), 125.9 (C-5, C-8), 123.5 (C-2′, C-6′), 31.7 (C-9), 13.1 (C-10) ppm.

*2-[(2-Fluoro-4-nitrophenyl)methyl]-3-methyl-1,4-naphthoquinone* (**2h**). Compound **2h** was obtained after stirring for 3 h, then purified by flash chromatography using toluene/cyclohexane (15:1). Yellow solid; yield: 58%; *R*_f_ = 0.48 (toluene:cyclohexane = 15:1); m.p.: 170–171 °C. ^1^H NMR (400 MHz, DMSO-*d*_6_) δ = 8.10 (dd, *J* = 9.9, 2.4 Hz, 1H, H-3′), 8.04 (m, 1H, H-5), 7.98 (m, 1H, H-8), 7.95 (m, 1H, H-5′), 7.85 (m, 2H, H-6, H-7), 7.53 (dd, *J* = 8.5, 7.7 Hz, 1H, H-6′), 4.09 (s, 2H, H-9), 2.15 (s, 3H, H-10) ppm; ^13^C NMR (100 MHz, DMSO-*d*_6_) δ = 184.3 (C-4), 183.6 (C-1), 159.4 (d, ^1^*J*_C,F_ = 248.7 Hz, C-2′), 146.8 (d, ^3^*J*_C,F_ = 9.1 Hz, C-4′), 145.8 (C-3), 141.9 (C-2), 134.0 (C-7), 133.9 (C-6), 133.5 (d, ^2^*J*_C,F_ = 15.8 Hz, C-1′), 131.7 (C-4a), 131.4 (C-8a), 131.0 (d, ^3^*J*_C,F_ = 4.6 Hz, C-6′), 125.9 (C-5, C-8), 119.5 (d, ^4^*J*_C,F_ = 3.4 Hz, C-5′), 110.9 (d, ^2^*J*_C,F_ = 27.6 Hz, C-3′), 25.6 (d, ^3^*J*_C,F_ = 2.8 Hz, C-9), 13.0 (C-10) ppm; HRMS (ESI) calcd. for C_18_H_11_FNO_4_ [M-H]^−^ = 324.0672; found: 324.0680.

*2-Methyl-3-[(2,4,5-trifluorophenyl)methyl]-1,4-naphthoquinone* (**2i**). Compound **2i** was obtained after stirring for 4 h, then purified by flash chromatography using toluene/cyclohexane (15:1). Yellow solid; yield: 55%; *R*_f_ = 0.39 (toluene:cyclohexane = 15:1); m.p.: 159–160 °C. ^1^H NMR (400 MHz, DMSO-*d*_6_) δ = 8.02 (m, 1H, H-5), 7.97 (m, 1H, H-8), 7.84 (m, 2H, H-6, H-7), 7.51 (ddd, *J* = 10.9, 9.7, 6.9 Hz, 1H, H-3′), 7.36 (ddd, *J* = 11.5, 9.0, 7.1 Hz, 1H, H-6′) 3.92 (s, 2H, H-9), 2.11 (s, 3H, H-10) ppm; ^13^C NMR (100 MHz, DMSO-*d*_6_) δ = 184.3 (C-4), 183.7 (C-1), 155.2 (ddd, ^1^*J*_C,F_ = 243.2, ^3^*J*_C_,_F_ = 9.8, ^4^*J*_C,F_ = 2.3 Hz, C-2′), 147.8 (ddd, ^1^*J*_C,F_ = 246.7, ^2^*J*_C,F_ = 14.3, ^3^*J*_C,F_ = 13.0 Hz, C-4′), 146.0 (ddd, ^1^*J*_C,F_ = 241.6, ^2^*J_C_*_,F_ = 12.4, ^4^*J*_C,F_ = 3.5 Hz, C-5′), 145.6 (C-3), 141.9 (C-2), 133.8* (C-6), 133.7* (C-7), 131.8 (C-4a), 131.5 (C-8a), 125.9* (C-5), 125.8* (C-8), 122.2 (ddd, ^2^*J*_C,F_ = 18.1, ^3^*J*_C,F_ = 5.9, ^4^*J*_C,F_ = 4.0 Hz, C-1′), 117.8 (dd, ^2^*J*_C,F_ = 19.8, ^3^*J*_C,F_ = 5.7 Hz, C-6′), 105.6 (dd, ^2^*J*_C,F_ = 29.1, ^3^*J*_C,F_ = 21.3 Hz, C-3′), 24.7 (d, ^3^*J*_C,F_ = 2.9 Hz, C-9), 12.8 (C-10) ppm; HRMS (APCI) calcd. for C_18_H_11_F_3_O_2_ [M]^−^ = 316.0711; found: 316.0721.

#### 3.1.3. General Synthetic Procedure for Acetamidobenzylmenadiones **2j**,**k** (Route C)

The respective aminophenylacetic acid (200 mg) was added to glacial acetic acid (3 mL) and subjected to microwave irradiation (180 W, 150 °C) for 40 min. The reaction mixture was poured into EtOAc (50 mL) and washed with H_2_O (15 mL) three times. The combined organic layers were dried over Na_2_SO_4_ and concentrated in vacuo to yield the crude products as orange solids, which were used without further purification.

As described in the Section 3.1.2, menadione (1 equiv.) was then added to a stirred solution of the above acetaminophenylacetic acid (2 equiv.) and treated with AgNO_3_ and (NH_4_)_2_S_2_O_8_ to give the corresponding acetamidobenzylmenadiones.

*2-[(4-Acetamidophenyl)methyl]-3-methyl-1,4-naphthoquinone* (**2j**). Compound **2j** was obtained after stirring for 1.5 h, then purified by flash chromatography using toluene/cyclohexane (15:1). Yellow solid; yield: 94%; *R*_f_ = 0.41 (toluene:cyclohexane = 15:1); m.p.: 196–197 °C (lit [35] m.p. 198–200 °C). The spectroscopic data were found to be identical to the ones described in [35]. Although **2j** represents an already known compound, to our knowledge the complete assignment of the NMR signals has not been published so far: ^1^H NMR (400 MHz, DMSO-*d*_6_) δ = 9.85 (s, 1H, NH), 8.00 (m, 2H, H-5, H-8), 7.83 (m, 2H, H-6, H-7), 7.45 (d, *J* = 8.6 Hz, 2H, H-3′, H-5′), 7.13 (d, *J* = 8.6 Hz, 2H, H-2′, H-6′), 3.92 (s, 2H, H-9), 2.14 (s, 3H, H-10, 1.99 (s, 3H, C*H*_3_-CO) ppm; ^13^C NMR (100 MHz, DMSO-*d*_6_) δ = 184.7 (C-4), 184.1 (C-1), 168.0 (*C*O-CH_3_), 144.5* (C-2), 144.1* (C-3), 137.4 (C-4′), 133.9* (C-6), 133.8* (C-7), 132.5 (C-1′), 131.6* (C-4a), 131.4* (C-8a), 128.5 (C-2′, C-6′), 125.9* (C-5), 125.8* (C-8), 119.1 (C-3′, C-5′), 31.0 (C-9), 23.8 (*C*H_3_-CO), 12.9 (C-10) ppm.

*2-[(3-Acetamidophenyl)methyl]-3-methyl-1,4-naphthoquinone* (**2k**). Compound **2k** was obtained after stirring for 1.5 h, then purified by flash chromatography using toluene/cyclohexane (15:1). Yellow solid; yield: 90%; *R*_f_ = 0.41 (toluene:cyclohexane = 15:1); m.p.: 188–189 °C (lit [35] m.p. 163–165 °C). The spectroscopic data were found to be identical to the ones described in [35]. Although **2k** represents an already known compound, to our knowledge the complete assignment of the NMR signals has not been published so far: ^1^H NMR (400 MHz, DMSO-*d*_6_) δ = 9.81 (s, 1H, NH), 8.01 (m, 2H, H-5, H-8), 7.84 (m, 2H, H-6, H-7), 7.49 (ddd, *J* = 8.2, 2.2, 1.1 Hz, 1H, H-4′), 7.31 (t, *J* = 1.9 Hz, 1H, H-2′), 7.18 (t, *J* = 7.8 Hz, 1H, H-5′), 6.91 (m, 1H, H-6′), 3.95 (s, 2H, H-9), 2.15 (s, 3H, H-10), 1.98 (s, 3H, C*H*_3_-CO) ppm; ^13^C NMR (100 MHz, DMSO-*d*_6_) δ = 184.6 (C-4), 183.9 (C-1), 168.1 (*C*O-CH_3_), 144.4* (C-2), 144.3* (C-3), 139.5 (C-3′), 138.5 (C-1′), 133.9 (C-6, C-7), 131.6* (C-4a), 131.4* (C-8a), 128.7 (C- 5′), 126.0* (C-5), 125.8* (C-8), 118.4 (C-2′), 116.8 (C-4′), 123.1 (C-6′), 31.6 (C-9), 23.9 (*C*H_3_-CO), 12.9 (C-10) ppm.

#### 3.1.4. General Synthetic Procedure for Amidobenzylmenadiones **2l**–**o** (Route C)

The acetamido derivatives **2j** resp. **2k** (200 mg) were added to a mixture of HCl *conc*. (0.5 mL) and MeOH (4 mL) and subjected to microwave irradiation (250 W, 150 °C) for 30 min. The reaction mixture was poured into EtOAc (50 mL) and washed with sat. aq Na_2_CO_3_ (15 mL) three times. The combined organic layers were dried over Na_2_SO_4_ and concentrated in vacuo to obtain crude products as orange solids, which were used without further purification.

The above prepared unprotected aminobenzyl derivative (1 equiv.) and dicyclohexyl-carbodiimide (DCC, 1.2 equiv.) were added in sequence to a stirred solution of the corresponding carboxylic acid (1.25 mmol, 1 equiv.) in anhydrous DMF (4 mL) under exclusion of air or humidity. The reaction mixture was stirred at rt until TLC showed complete consumption of the starting material (3–4 h). The mixture was then filtered and the residue extracted with MTBE (20 mL). The combined organic layers were washed successively with H_2_O, 1M HCl, and sat. aq Na_2_CO_3_, dried over Na_2_SO_4_, and concentrated in vacuo to give a residue, which was purified by flash chromatography as detailed below.

*2-Methyl-3-{[4-(2,2,2-trifluoroacetamido)phenyl]methyl}-1,4-naphthoquinone* (**2l**). Compound **2l** was obtained after stirring for 3 h, then purified by flash chromatography using toluene/cyclohexane (15:1). Yellow solid; yield: 71%; *R*_f_ = 0.51 (toluene:cyclohexane = 15:1); m.p.: 194–195 °C. ^1^H NMR (400 MHz, DMSO-*d*_6_) δ = 11.19 (s, 1H, NH), 8.01 (m, 2H, H-5, H-8), 7.84 (m, 2H, H-6, H-7), 7.56 (m, 2H, H-3′, H-5′), 7.27 (m, 2H, H-2′, H-6′), 3.98 (s, 2H, H-9), 2.16 (s, 3H, H-10) ppm; ^13^C NMR (100 MHz, DMSO-*d*_6_) δ = 184.6 (C-4), 184.0 (C-1), 154.3 (q, ^2^*J*_C,F_ = 36.8 Hz, *C*O-CF_3_), 144.4* (C-3), 144.1* (C-2), 135.6 (C-1′), 134.3 (C-4′), 133.9* (C-6), 133.8* (C-7), 131.7* (C-4a), 131.4* (C-8a), 128.8 (C-2′, C-6′), 125.9* (C-5), 125.8* (C-8), 121.2 (C-3′, C-5′), 115.7 (q, ^1^*J*_C,F_ = 288.9 Hz, CF_3_), 31.2 (C-9), 12.9 (C-10) ppm; HRMS (ESI) calcd. for C_20_H_13_F_3_NO_3_ [M-H]^−^ = 372.0848; found: 372.0854.

*2-Methyl-3-{[3-(2,2,2-trifluoroacetamido)phenyl]methyl}-1,4-naphthoquinone* (**2m**). Compound **2m** was obtained after stirring for 3 h, then purified by flash chromatography using toluene/cyclohexane (15:1). Yellow solid; yield: 31%; *R*_f_ = 0.53 (toluene:cyclohexane = 15:1); m.p.: 194–195 °C. ^1^H NMR (400 MHz, DMSO-*d*_6_) δ = 11.12 (s, 1H, NH), 8.01 (m, 2H, H-5, H-8), 7.84 (m, 2H, H-6, H-7), 7.57 (ddd, *J* = 7.9, 2.2, 1.1 Hz, 1H, H-4′), 7.45 (t, *J* = 1.9 Hz, 1H, H-2′), 7.31 (t, *J* = 7.9 Hz, 1H, H-5′), 7.13 (ddd, *J* = 7.9, 1.8, 1.0 Hz, 1H, H-6′), 4.00 (s, 2H, H-9), 2.17 (s, 3H, H-10) ppm; ^13^C NMR (100 MHz, DMSO-*d*_6_) δ = 184.6 (C-4), 183.9 (C-1), 154.3 (q, ^2^*J*_C,F_ = 37.0 Hz, *C*O-CF_3_), 144.5* (C-2), 144.1* (C-3), 139.1 (C-1′), 136.5 (C-3′), 133.9 (C-6, C-7), 131.6* (C-4a), 131.4* (C-8a), 129.0 (C-5′), 126.0* (C-5), 125.9* (C-8), 125.8 (C-6′), 118.7 (C-4′), 120.4 (C-2′), 115.6 (q, ^1^*J*_C,F_ = 288.8 Hz, CF_3_), 31.6 (C-9), 13.0 (C-10) ppm; HRMS (ESI) calcd. for C_20_H_13_F_3_NO_3_ [M-H]^−^ = 372.0848; found: 372.0855.

*2-Methyl-2-{[4-(4,4,4-trifluorobutanamido)phenyl]methyl}-1,4-naphthoquinone* (**2n**). Compound **2n** was obtained after stirring for 4 h, then purified by flash chromatography using toluene/cyclohexane (15:1). Yellow solid; yield: 70%; *R*_f_ = 0.40 (toluene:cyclohexane = 15:1); m.p.: 180–181 °C. ^1^H NMR (400 MHz, DMSO-*d*_6_) δ = 10.00 (s, 1H, NH), 8.01 (m, 2H, H-5, H-8), 7.84 (m, 2H, H-6, H-7), 7.46 (d, *J* = 8.4 Hz, 2H, H-3′, H-5′), 7.16 (d, *J* = 8.4 Hz, 2H, H-2′, H-6′), 3.93 (s, 2H, H-9), 2.57 (m, 4H, H-2″, H-3″), 2.15 (s, 3H, H-10) ppm; ^13^C NMR (100 MHz, DMSO-*d*_6_) δ = 184.7 (C-4), 184.1 (C-1), 168.0 (C-1″), 144.5* (C-2), 144.1* (C-3), 137.1 (C-4′), 133.9* (C-6), 133.8* (C-7), 132.9 (C-1′), 131.6* (C-4a), 131.4* (C-8a), 128.6 (C-2′, C-6′), 127.4 (q, ^1^*J*_C,F_ = 276.2 Hz, C-4″), 125.9 (C-5, C-8), 119.2 (C-3′, C-5′), 31.1 (C-9), 28.5 (C-2″), 28.4 (q, ^2^*J*_C,F_ = 28.7 Hz, C-3″), 12.9 (C-10) ppm; HRMS (ESI) calcd. for C_22_H_17_F_3_NO_3_ [M-H]^−^ = 400.1161; found: 400.1168.

*2-Methyl-3-{[3-(4,4,4-trifluorobutanamido)phenyl]methyl}-1,4-naphthoquinone* (**2o**). Compound **2o** was obtained after stirring for 4 h, then purified by flash chromatography using toluene/cyclohexane (15:1). Yellow solid; yield: 16%; *R*_f_ = 0.42 (toluene:cyclohexane = 15:1); m.p.: 180–181 °C. ^1^H NMR (400 MHz, DMSO-*d*_6_) δ = 9.97 (s, 1H, NH), 8.01 (m, 2H, H-5, H-8), 7.85 (m, 2H, H-6, H-7), 7.50 (dt, *J* = 7.9, 1.3 Hz, 1H, H-4′), 7.33 (t, *J* = 1.3 Hz, 1H, H-2′), 7.21 (t, *J* = 7.9 Hz, 1H, H-5′), 6.95 (dt, *J* = 7.9, 1.3 Hz, 1H, H-6′), 3.96 (s, 2H, H-9), 2.54 (m, 2H, H-2″), 2.51 (m, 2H, H-3″), 2.16 (s, 3H, H-10) ppm; ^13^C NMR (100 MHz, DMSO-*d*_6_) δ = 184.6 (C-4), 183.9 (C-1), 168.1 (C-1″), 144.4* (C-2), 144.3* (C-3), 139.1 (C-3′), 138.7 (C-1′), 134.0* (C-6), 133.9* (C-7), 131.6* (C-4a), 131.4* (C-8a), 128.8 (C-5′), 127.4 (q, ^1^*J*_C,F_ = 276.2 Hz, C-4″), 126.0* (C-5), 125.9* (C-8), 123.4 (C-6′), 118.5 (C-2′), 116.9 (C-4′), 31.6 (C-9), 28.6 (q, ^3^*J*_C,F_ = 2.9 Hz, C-2″), 28.3 (q, ^2^*J*_C,F_ = 28.4 Hz, C-3″), 12.9 (C-10) ppm; HRMS (ESI) calcd. for C_22_H_19_F_3_NO_3_ [M+H]^+^ = 402.1317; found: 402.1305.

#### 3.1.5. General Synthetic Procedure for Benzylmenadiones **2p**–**s** (Route D)

Tetralone (200 mg, 1 equiv.) and the respective aldehyde (1.1 equiv.) were added successively to a stirred solution of KOH (1.2 equiv.) in EtOH (5 mL) at rt. The stirring was continued until the TLC showed complete consumption of the starting material (2.5–4 h). The mixture was poured into 30 mL of ice-cold water, and a white precipitate was formed. The product was filtered, washed with ice-cold water and concentrated in vacuo to give a beige solid, which was used without further purification.

Trichlororhodium trihydrate (0.1 equiv.) was added to a stirred solution of the benzylidene derivative prepared above (200 mg, 1 equiv.) in well-degassed EtOH (5 mL) under strict exclusion of air or moisture. The mixture was refluxed until the TLC showed complete consumption of the starting material (24 h). After concentration, the residue was partitioned between H_2_O (15 mL) and EtOAC (50 mL) and the aqueous layer was extracted again with EtOAc (30 mL). The combined organic layers were dried over Na_2_SO_4_ and concentrated in vacuo to obtain the crude product, which was used without further purification.

(Diacetoxyiodo)benzene (PIDA) (2.1 equiv.) was added in portions to a stirred solution of above prepared naphthol (200 mg, 1 equiv.) in acetonitrile (5 mL) and water (2 mL) at −5 °C for 20–30 min. The reaction mixture was allowed to reach ambient temperature and stirred until the TLC showed complete consumption of the starting material (1 h). After removal of CH_3_CN under reduced pressure, the resulting residue was extracted three times with CH_2_Cl_2_ (20 mL). The combined organic phases were washed with a 1M aqueous solution of triethylammonium acetate, dried over Na_2_SO_4_ and concentrated in vacuo to give a yellow solid, which was used without further purification.

The above-prepared 2-benzyl naphthoquinone derivative (200 mg, 1 equiv.) and acetic acid (5 equiv.) were added to a stirred solution of CH_3_CN/H_2_O (3:1, 12 mL) and heated to 85 °C. AgNO_3_ (0.35 equiv.) was added first and then (NH_4_)_2_S_2_O_8_ (1.3 equiv.) dissolved in 4 mL CH_3_CN/H_2_O (3:1) dropwise over a period of 5 min. Stirring at 85 °C was continued until TLC showed complete consumption of the starting material (3–4 h). After cooling to ambient temperature, the mixture was extracted three times with CH_2_Cl_2_ (20 mL). The combined organic layers were washed with H_2_O, dried over Na_2_SO_4_ and concentrated in vacuo to give a residue, which was purified by flash chromatography as detailed below.

*2-[(4-Fluorophenyl)methyl]-3-methyl-1,4-naphthoquinone* (**2p**). Compound **2p** was purified by flash chromatography using toluene/cyclohexane (15:1). Yellow solid; yield: 17%; *R*_f_ = 0.52 (toluene:cyclohexane = 15:1); m.p.: 108–109 °C (lit [28] m.p. 114–115 °C). The spectroscopic data were found to be identical to the ones described in [28]. Although **2p** represents an already known compound, to our knowledge the complete assignment of the NMR signals has not been published so far: ^1^H NMR (400 MHz, DMSO-*d*_6_) δ = 7.26 (m, 2H, H-2′, H-6′), 7.08 (t, *J* = 8.9 Hz, 2H, H-3′, H-5′), 3.96 (s, 2H, H-9), 2.15 (s, 3H, H-10) ppm; ^13^C NMR (100 MHz, DMSO-*d*_6_) δ = 184.6 (C-4), 184.0 (C-1), 160.7 (d, ^1^*J*_C,F_ = 242.0 Hz, C-4′), 144.3* (C-2), 144.2* (C-3), 134.2 (d, ^4^*J*_C,F_ = 2.9 Hz, C-1′), 133.9* (C-6), 133.8* (C-7), 131.6* (C-4a), 131.4* (C-8a), 130.1 (d, ^3^*J*_C,F_ = 8.0 Hz, C-2′, C-6′), 125.9* (C-5), 125.8* (C-8), 115.1 (d, ^2^*J*_C,F_ = 21.1 Hz, C-3′, C-5′), 30.8 (C-9), 12.9 (C-10) ppm.

*2-Methyl-3-{[4-(trifluoromethoxy)phenyl]methyl}-1,4-naphthoquinone* (**2q**). Compound **2q** was purified by flash chromatography using toluene/cyclohexane (15:1). Yellow solid; yield: <5%; *R*_f_ = 0.59 (toluene:cyclohexane = 15:1); m.p.: 72–73 °C (lit [28] m.p. 65–66 °C). The spectroscopic data were found to be identical to the ones described in [28]. Although **2p** represents an already known compound, to our knowledge the complete assignment of the NMR signals has not been published so far: ^1^H NMR (400 MHz, DMSO-*d*_6_) δ = 8.01 (m, 2H, H-5, H-8), 7.84 (m, 2H, H-6, H-7), 7.36 (d, *J* = 8.8 Hz, 2H, H-2′, H-6′), 7.25 (d, *J* = 8.2 Hz, 2H, H-3′, H-5′), 4.01 (s, 2H, H-9), 2.16 (s, 3H, H-10) ppm; ^13^C NMR (100 MHz, DMSO-*d*_6_) δ = 184.5 (C-4), 184.0 (C-1), 146.6 (q, ^3^*J*_C,F_ = 1.8 Hz, C-4′), 144.6* (C-2), 143.8* (C-3), 137.7 (C-1′), 133.9* (C-6), 133.8* (C-7), 131.7* (C-4a), 131.4* (C-8a), 130.1 (C-2′, C-6′), 125.9* (C-5), 125.8* (C-8), 121.1 (C-3′, C-5′), 120.0 (q, ^1^*J*_C,F_ = 255.8 Hz, CF_3_), 30.9 (C-9), 12.9 (C-10) ppm.

*2-Methyl-3-{[3-(trifluoromethoxy)phenyl]methyl}-1,4-naphthoquinone* (**2r**). Compound **2r** was purified by flash chromatography using toluene/cyclohexane (15:1). Yellow solid; yield: <5%; *R*_f_ = 0.59 (toluene:cyclohexane = 15:1); m.p.: 70–71 °C. ^1^H NMR (400 MHz, DMSO-*d*_6_) δ = 8.00 (m, 2H, H-5, H-8), 7.84 (m, 2H, H-6, H-7), 7.40 (t, *J* = 8.1 Hz, 1H, H-5′), 7.25 (m, 2H, H-2′, H-6′), 7.18 (ddt, *J* = 8.1, 2.4, 1.1 Hz, 1H, H-4′), 4.04 (s, 2H, H-9), 2.14 (s, 3H, H-10) ppm; ^13^C NMR (100 MHz, DMSO-*d*_6_) δ = 184.5 (C-4), 184.0 (C-1), 148.4 (q, ^3^*J*_C,F_ = 1.6 Hz, C-3′), 144.8* (C-2), 143.4* (C-3), 141.0 (C-1′), 133.9* (C-6), 133.8* (C-7), 131.7* (C-4a), 131.4* (C-8a), 130.3 (C-5′), 125.9* (C-5), 125.8* (C-8), 127.3 (C-6′), 120.9 (C-2′), 120.0 (q, ^1^*J*_C,F_ = 256.1 Hz, CF_3_), 118.5 (C-4′), 31.3 (C-9), 12.9 (C-10) ppm; HRMS (APCI) calcd. for C_19_H_13_F_3_O_3_ [M]^−^ = 346.0817; found: 346.0829.

*2-{[2-Fluoro-5-(trifluoromethoxy)phenyl]methyl}-3-methyl-1,4-naphthoquinone* (**2s**). Compound **2s** was purified by flash chromatography using toluene/cyclohexane (15:1). Yellow solid; yield: <5%; *R*_f_ = 0.58 (toluene:cyclohexane = 15:1); m.p.: 78–79 °C. ^1^H NMR (400 MHz, DMSO-*d*_6_) δ = 8.03 (m, 1H, H-5), 7.99 (m, 1H, H-8), 7.84 (m, 2H, H-6, H-7), 7.33 (t, *J* = 9.1 Hz, 1H, H-3′), 7.29 (d, *J* = 5.4 Hz, 1H, H-6′), 7.28 (m, 1H, H-4′), 4.01 (s, 2H, H-9), 2.12 (s, 3H, H-10) ppm; ^13^C NMR (100 MHz, DMSO-*d*_6_) δ = 184.3 (C-4), 183.7 (C-1), 158.6 (d, ^1^*J*_C,F_ = 244.5 Hz, C-2′), 145.6* (C-2), 144.2 (C-5′), 142.0* (C-3), 133.9* (C-6), 133.8* (C-7), 131.8 (C-4a), 131.5 (C-8a), 127.3 (d, ^2^*J*_C,F_ = 18.1 Hz, C-1′), 125.9 (C-5), 125.8 (C-8), 123.2 (d, ^3^*J*_C,F_ = 5.1 Hz, C-6′), 120.9 (d, ^3^*J*_C,F_ = 9.1 Hz, C-4′), 119.9 (q, ^1^*J*_C,F_ = 256.1 Hz, CF_3_), 116.7 (d, ^2^*J*_C,F_ = 24.8 Hz, C-3′), 25.3 (d, ^3^*J*_C,F_ = 2.9 Hz, C-9), 12.8 (C-10) ppm; HRMS (APCI) calcd. for C_19_H_12_F_4_O_3_ [M]^−^ = 364.0723; found: 364.0736.

#### 3.1.6. General Synthetic Procedure for Benzylnaphthoquinones **4a**–**e** (Route B)

AgNO_3_ (0.1 equiv.) and (NH_4_)_2_S_2_O_8_ (2 equiv.) were dissolved in water (4 mL) and a solution of 1,4-naphthoquinone (200 mg, 1 equiv.) and the respective phenylacetic acid derivative (1.4 equiv.) in a 1:1 mixture of CH_3_CN/CH_2_Cl_2_ (4 mL) was quickly added. The two-phase mixture was heated to 80 °C and stirred until the TLC showed complete consumption of the starting material (1.5–2.5 h). After cooling to ambient temperature, the mixture was extracted three times with CH_2_Cl_2_ (20 mL). The combined organic layers were washed three times with H_2_O, dried over Na_2_SO_4_, and concentrated in vacuo to give a residue, which was purified by flash chromatography as detailed below.

*2-Benzyl-1,4-naphthoquinone* (**4a**). Compound **4a** was obtained after stirring for 1.5 h and purified by flash chromatography using toluene/cyclohexane (15:1). Amber solid; yield: 75%; *R*_f_ = 0.41 (toluene:cyclohexane = 15:1); m.p.: 92–93 °C (lit [70] m.p. 85–86 °C). The spectroscopic data were found to be identical to the ones described in [70]. Although **4a** represents an already known compound, to our knowledge the complete assignment of the NMR signals has not been published so far: ^1^H NMR (400 MHz, CDCl_3_) δ = 8.11 (m, 1H, H-8), 8.04 (m, 1H, H-5), 7.72 (m, 2H, H-6, H-7), 7.34 (m, 2H, H-3′, H-5′), 7.26 (m, 1H, H-4′), 7.25 (m, 2H, H-2′, H-6′), 6.61 (t, *J* = 1.5 Hz, 1H, H-3), 3.90 (s, 2H, H-9) ppm; ^13^C NMR (100 MHz, CDCl_3_): δ = 185.2 (C-4), 185.0 (C-1), 150.9 (C-2), 136.7 (C-1′), 135.6 (C-3), 133.8* (C-6), 133.7* (C-7), 132.2 (C-8a), 132.1 (C-4a), 129.4 (C-2′, C-6′), 128.9 (C-3′, C-5′), 127.0 (C-4′), 126.7 (C-8), 126.1 (C-5), 35.7 (C-9) ppm.

*2-{[4-(Trifluoromethyl)phenyl]methyl}-1,4-naphthoquinone* (**4b**). Compound **4b** was obtained after stirring for 2 h and purified by flash chromatography using toluene/cyclohexane (15:1). Amber solid; yield: 66%; *R*_f_ = 0.36 (toluene:cyclohexane = 15:1); m.p.: 99–100 °C (lit [26] m.p. 98–100 °C). The spectroscopic data were found to be identical to the ones described in [26]. Although **4b** represents an already known compound, to our knowledge the complete assignment of the NMR signals has not been published so far: ^1^H NMR (400 MHz, DMSO-*d*_6_) δ = 7.99 (m, 1H, H-8), 7.95 (m, 1H, H-5), 7.84 (m, 2H, H-6, H-7), 7.67 (d, *J* = 7.9 Hz, 2H, H-3′, H-5′), 7.55 (d, *J* = 7.9 Hz, 2H, H-2′, H-6′), 6.85 (s, 1H, H-3), 3.96 (s, 2H, H-9) ppm; ^13^C NMR (100 MHz, DMSO-*d*_6_): δ = 184.5 (C-4), 184.2 (C-1), 149.3 (C-2), 142.6 (C-1′), 135.8 (C-3), 134.1* (C-6), 134.0* (C-7), 131.6 (C-4a, C-8a), 129.9 (C-2′, C-6′), 127.2 (q, ^2^*J*_C,F_ = 31.8 Hz, C-4′), 126.1 (C-8), 125.6 (C-5), 125.3 (q, ^3^*J*_C,F_ = 3.9 Hz, C-3′, C-5′), 124.2 (q, ^1^*J*_C,F_ = 272.1 Hz, CF_3_), 34.7 (C-9) ppm.

*2-{[2-Fluoro-4-(trifluoromethyl)phenyl]methyl}-1,4-naphthoquinone* (**4c**). Compound **4c** was obtained after stirring for 2.5 h and purified by flash chromatography using toluene/cyclohexane (15:1). Amber solid; yield: 58%; *R*_f_ = 0.58 (toluene:cyclohexane = 15:1); m.p.: 130–131 °C. ^1^H NMR (400 MHz, CDCl_3_) δ = 8.12 (m, 1H, H-5), 8.05 (m, 1H, H-8), 7.75 (m, 2H, H-6, H-7), 7.45 (m, 1H, H-6′), 7.41 (m, 1H, H-5′), 7.35 (d, *J* = 9.7 Hz, 1H, H-3′), 6.62 (d, *J* = 1.6 Hz, 1H, H-3), 3.99 (s, 2H, H-9) ppm; ^13^C NMR (100 MHz, CDCl_3_): δ = 184.8 (C-4), 184.5 (C-1), 160.8 (d, ^1^*J*_C,F_ = 249.2 Hz, C-2′), 148.1 (C-2), 135.8 (d, ^5^*J*_C,F_ = 1.1 Hz, C-3), 134.0 (C-7), 133.9 (C-6), 132.4 (d, ^3^*J*_C,F_ = 4.6 Hz, C-6′), 132.0 (C-4a, C-8a), 131.6 (qd, ^2^*J*_C,F_ = 33.5, ^3^*J*_C,F_ = 8.0 Hz, C-4′), 128.2 (dq, ^2^*J*_C,F_ = 15.7, ^5^*J*_C,F_ = 1.0 Hz, C- 1′), 126.7 (C-5), 126.2 (C-8), 123.2 (qd, ^1^*J*_C,F_ = 272.5, ^4^*J*_C,F_ = 2.3 Hz, CF_3_), 121.6 (C-5′), 113.2 (dq, ^2^*J*_C,F_ = 25.1, ^3^*J*_C,F_ = 3.7 Hz, C-3′), 29.2 (C-9) ppm; HRMS (ESI) calcd. for C_18_H_9_F_4_O_2_ [M-H]^−^ = 333.0544; found: 333.0550.

*2-{[4-Fluoro-2-(trifluoromethyl)phenyl]methyl}-1,4-naphthoquinone* (**4d**). Compound **4d** was obtained after stirring for 2.5 h and purified by flash chromatography using toluene/cyclohexane (15:1). Amber solid; yield: 61%; *R*_f_ = 0.43 (toluene:cyclohexane = 15:1); m.p.: 132–133 °C. ^1^H NMR (400 MHz, CDCl_3_) δ = 8.15 (m, 1H, H-5), 8.05 (m, 1H, H-8), 7.76 (m, 2H, H-6, H-7), 7.44 (dd, *J* = 9.0, 2.6 Hz, 1H, H-3′), 7.29 (m, 1H, H-6′), 7.25 (td, *J* = 8.4, 2.6 Hz, 1H, H-5′), 6.27 (s, 1H, H-3), 4.08 (s, 2H, H-9) ppm; ^13^C NMR (100 MHz, CDCl_3_): δ = 184.7 (C-4), 184.6 (C-1), 161.3 (d, ^1^*J*_C,F_ = 248.7 Hz, C-4′), 150.1 (C-2), 135.8 (C-3), 134.3 (d, ^3^*J*_C,F_ = 7.7 Hz, C-6′), 133.9 (C-6, C-7), 132.0 (C-4a, C-8a), 131.2 (qd, ^2^*J*_C,F_ = 31.4, ^3^*J*_C,F_ = 7.4 Hz, C-2′), 130.8 (C-1′), 126.8 (C-5), 126.2 (C-8), 123.3 (qd, ^1^*J*_C,F_ = 274.1, ^4^*J*_C,F_ = 2.7 Hz, CF_3_), 119.3 (dq, ^2^*J*_C,F_ = 20.7, ^5^*J*_C,F_ = 1.1 Hz, C-5′), 114.3 (dq, ^2^*J*_C,F_ = 25.0, ^3^*J*_C,F_ = 5.7 Hz, C-3′) 31.7 (C-9) ppm; HRMS (ESI) calcd. for C_18_H_9_F_4_O_2_ [M-H]^−^ = 333.0544; found: 333.0549.

*2-[(6-Chloropyridin-3-yl)methyl]-1,4-naphthoquinone* (**4e**). Compound **4e** was obtained after stirring for 2 h and purified by flash chromatography using toluene/cyclohexane (15:1). Amber solid; yield: 55%; *R*_f_ = 0.57 (toluene:cyclohexane = 15:1); m.p.: 154–155 °C. ^1^H NMR (400 MHz, CDCl_3_) δ = 8.34 (d, *J* = 2.5 Hz, 1H, H-2′), 8.09 (dd, *J* = 5.8, 3.4 Hz, 1H, H-8), 8.06 (dd, *J* = 5.7, 3.4 Hz, 1H, H-5), 7.75 (m, 2H, H-6, H-7), 7.58 (dd, *J* = 8.2, 2.5 Hz, 1H, H-4′), 7.30 (d, 8.2 Hz, 1H, H-5′), 6.68 (s, 1H, H-3), 3.88 (s, 2H, H-9) ppm; ^13^C NMR (100 MHz, CDCl_3_): δ = 184.7 (C-4), 184.6 (C-1), 150.3 (C-2′, C-6′), 149.0 (C-2), 139.7 (C- 4′), 135.9 (C-3), 134.1* (C-6), 134.0* (C-7), 132.0* (C-4a), 131.9* (C-8a), 131.6 (C-3′), 126.8 (C-8), 126.3 (C-5), 124.4 (C-5′), 32.6 (C-9) ppm; HRMS (APCI) calcd. for C_16_H_11_ClNO_2_ [M+H]^+^ = 284.0473; found: 284.0465.

#### 3.1.7. General Synthetic Procedure for Trifluoromenadiones **5a**–**c** (Route B)

CuI (0.5 equiv.), bis(pinacolato)diboron (B_2_pin_2_, 0.01 equiv.), and Togni reagent (2 equiv.) were dissolved in CHCl_3_ (3 mL) in the strict exclusion of air or moisture, and the mixture was refluxed in an oil bath at 85 °C. The corresponding benzylnaphthoquinone derivative (200 mg. 1 equiv.) dissolved in 5 mL of CHCl_3_ was then added, and stirring was continued at 85 °C until TLC showed complete consumption of the starting material (20–26 h). After cooling to ambient temperature, the mixture was diluted with CHCl_3_ (20 mL) and washed with H_2_O (15 mL) three times. The organic layer was dried over Na_2_SO_4_ and then concentrated in vacuo to give a residue, which was purified by flash chromatography as detailed below.

*2-Benzyl-3-(trifluoromethyl)-1,4-naphthoquinone* (**5a**). Compound **5a** was obtained from **4a** after stirring for 20 h and purified by flash chromatography using toluene/cyclohexane (15:1). Yellow solid; yield: 60%; *R*_f_ = 0.49 (toluene:cyclohexane = 15:1); m.p.: 72–73 °C. ^1^H NMR (400 MHz, DMSO-*d*_6_) δ = 8.06 (m, 1H, H-5), 8.03 (m, 1H, H-8), 7.93 (m, 1H, H-6), 7.89 (m, 1H, H-7), 7.28 (m, 2H, H-3′, H-5′), 7.20 (dd, *J* = 7.5, 1.8 Hz, 2H, H-2′, H-6′), 7.18 (m, 1H, H-4′), 4.18 (s, 2H, H-9) ppm; ^13^C NMR (100 MHz, DMSO-*d*_6_): δ = 183.3 (C-1), 180.3 (C-4), 149.2 (q, ^3^*J*_C,F_ = 1.8 Hz, C-2), 137.5 (C-1′), 134.7 (C-6), 134.4 (C-7), 132.4 (q, ^2^*J*_C,F_ = 27.0 Hz, C-3), 131.6 (q, ^4^*J*_C,F_ = 1.5 Hz, C-4a), 131.0 (C-8a), 128.4 (C-3′, C-5′), 127.9 (C-2′, C-6′), 126.4 (C-8), 126.2 (C-4′), 125.9 (C-5), 122.4 (q, ^1^*J*_C,F_ = 278.1 Hz, C-10), 31.7 (q, ^4^*J*_C,F_ = 3.3 Hz, C-9) ppm; HRMS (APCI) calcd. for C_18_H_11_F_3_O_2_ [M]^−^ = 316.0711; found: 316.0719.

*2-(Trifluoromethyl)-3-{[4-(trifluoromethyl)phenyl]methyl}-1,4-naphthoquinone* (**5b**). Compound **5b** was obtained from **4b** after stirring for 24 h and purified by flash chromatography using toluene/cyclohexane (15:1). Yellow solid; yield: 57%; *R*_f_ = 0.32 (toluene:cyclohexane = 15:1); m.p.: 83–84 °C. ^1^H NMR (400 MHz, DMSO-*d*_6_) δ = 8.07 (m, 1H, H-5), 8.03 (m, 1H, H-8), 7.94 (td, *J* = 7.4, 1.7 Hz, 1H, H-6), 7.90 (td, *J* = 7.4, 1.6 Hz, 1H, H-7), 7.63 (d, *J* = 8.2 Hz, 2H, H-3′, H-5′), 7.46 (d, *J* = 8.2 Hz, 2H, H-2′, H-6′), 4.26 (s, 2H, H-9) ppm; ^13^C NMR (100 MHz, DMSO-*d*_6_): δ = 183.2 (C-1), 180.2 (C-4), 148.4 (q, ^3^*J*_C,F_ = 1.8 Hz, C-2), 142.6 (C-1′), 134.7 (C-6), 134.4 (C-7), 132.9 (q, ^2^*J*_C,F_ = 27.1 Hz, C-3), 131.7 (q, ^4^*J*_C,F_ = 1.5 Hz, C-4a), 131.1 (C-8a), 128.8 (C-2′, C-6′), 127.0 (q, ^2^*J*_C,F_ = 31.9 Hz, C-4′), 126.4 (C-8), 125.9 (C-5), 125.2 (q, ^3^*J*_C,F_ = 3.9 Hz, C-3′, C-5′), 124.2 (q, ^1^*J*_C,F_ = 278.1 Hz, 4′-CF_3_), 122.3 (q, ^1^*J*_C,F_ = 278.3 Hz, C-10), 31.7 (q, ^4^*J*_C,F_ = 3.2 Hz, C-9) ppm; HRMS (APCI) calcd. for C_19_H_10_F_6_O_2_ [M]^−^ = 384.0585; found: 384.0584.

*2-{[2-Fluoro-4-(trifluoromethyl)phenyl]methyl}-3-(trifluoromethyl)-1,4-naphthoquinone* (**5c**). Compound **5c** was obtained from **4c** after stirring for 26 h and purified by flash chromatography using toluene/cyclohexane (15:1). Yellow solid; yield: 40%; *R*_f_ = 0.30 (toluene:cyclohexane = 15:1); m.p.: 75–76 °C. ^1^H NMR (400 MHz, DMSO-*d*_6_) δ = 8.09 (m, 1H, H-5), 8.04 (m, 1H, H-8), 7.95 (td, *J* = 7.5, 1.7 Hz, 1H, H-6), 7.92 (td, *J* = 7.4, 1.6 Hz, 1H, H-7), 7.69 (m, 1H, H-3′), 7.47 (m, 2H, H-5′, H-6′), 4.21 (s, 2H, H-9) ppm; ^13^C NMR (100 MHz, DMSO-*d*_6_): δ = 182.9 (C-1), 180.1 (C-4), 159.5 (d, ^1^*J*_C,F_ = 247.4 Hz, C-2′), 147.6 (q, ^3^*J*_C,F_ = 2.8 Hz, C-2), 134.8 (C-6), 134.5 (C-7), 133.3 (q, ^2^*J*_C,F_ = 27.2 Hz, C-3), 131.6 (C-4a), 131.1 (C-8a), 130.8 (qd, ^2^*J*_C,F_ = 33.2, ^3^*J*_C,F_ = 8.0 Hz, C-4′, C-6′), 129.6 (d, ^2^*J*_C,F_ = 32.2 Hz, C-1′), 126.4 (C-8), 126.0 (C-5), 123.3 (qd, ^1^*J*_C,F_ = 272.0, ^4^*J*_C,F_ = 2.5 Hz, 4′-CF_3_), 122.3 (q, ^1^*J*_C,F_ = 278.1 Hz, C-10), 121.4 (C-5′), 112.6 (dq, ^2^*J*_C,F_ = 25.6, ^3^*J*_C,F_ = 3.8 Hz, C-3′), 25.4 (dq, ^3^*J*_C,F_ = 6.1, ^4^*J*_C,F_ = 3.1 Hz, C-9) ppm; HRMS (APCI) calcd. for C_19_H_9_F_7_O_2_ [M]^−^ = 402.0498; found: 402.0501.

## 4. Conclusions

In this study, we present the synthesis of 27 plasmodione-like benzylnaphthoquinone derivatives together with their antiprotozoal activities and physicochemical parameters. The antiplasmodial potential was found highly promising, which was also confirmed by remarkably high selectivities. Ligand efficiency metrics, specifically size-independent ligand efficiency (SILE), correlated strongly with antiplasmodial activity, thus guiding drug optimization.

The four-fold fluorinated benzylmenadione **2e** showed the lowest IC_50_ of all prepared compounds against the *P. falciparum* NF54 strain at 0.006 μM along with very good selectivity (SI = 7495). The trypanocidal effect among the synthesized compounds was also assessed. It was found strongest in benzyl-NQ, **4a**, albeit much less pronounced.

The findings presented in this work demonstrate the potential of benzylmenadiones as promising chemical leads in the development of new antiplasmodial drugs.

## Figures and Tables

**Table 1:** Synthesis of benzyl-substituted naphthoquinone and menadione derivatives (overall yields calculated from NQ, MD, and tetralone).

Compd	Structure	Route	Yield (%)	Compd	Structure	Route	Yield (%)
**2a**	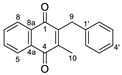	A	88	**2o**	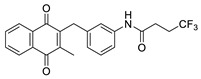	C	14
**2b**	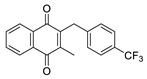	A	83	**2p**	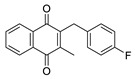	D	17
**2c**	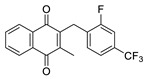	A	69	**2q**	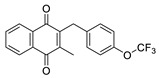	D	<5
**2d**	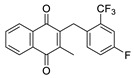	A	66	**2r**	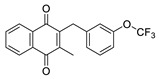	D	<5
**2e**	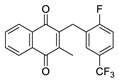	A	63	**2s**	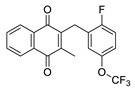	D	<5
**2f**	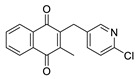	A	60	**4a**	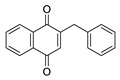	B	75
**2g**	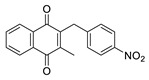	A	66	**4b**	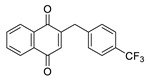	B	66
**2h**	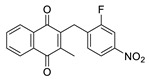	A	58	**4c**	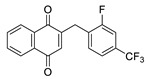	B	58
**2i**	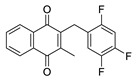	A	55	**4d**	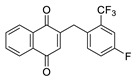	B	61
**2j**	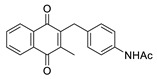	C	94	**4e**	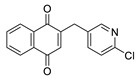	B	55
**2k**	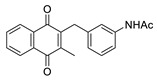	C	90	**5a**	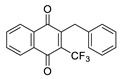	B	45
**2l**	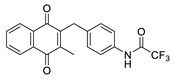	C	67	**5b**	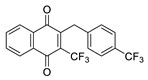	B	38
**2m**	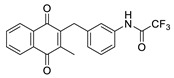	C	28	**5c**	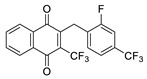	B	23
**2n**	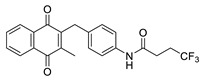	C	66				

**Table 2:** In vitro antiparasitic activity, mammalian cell toxicity, and predicted oral toxicity of the synthesized compounds.

Compd	*P. falc.* ^1^	SI ^2^	*P. falc.* ^1^	*T. b. rhod.* ^3^	SI ^2^	*T. b. rhod.* ^3^	Cyt L6 ^4^	Oral Toxicity ^6^
	IC_50_ μM		pIC_50_^5^	IC_50_ μM		pIC_50_^5^	IC_50_ μM	LD_50_ mg/kg
Chl.	0.003	30367	8.52				91.1	
Mel.				0.006	4033	8.22	24.2	
Pod.							0.024	
**2a**	0.358	126	6.45	62.793	1	4.20	45.023	500
**2b**	0.048	994	7.31	65.276	1	4.19	48.152	500
**2c**	0.011	5377	7.94	81.396	1	4.09	61.746	500
**2d**	0.020	3099	7.70	23.210	3	4.63	62.274	500
**2e**	**0.006**	**7495**	**8.24**	93.021	0	4.03	43.038	2000
**2f**	0.037	654	7.43	8.269	3	5.08	24,172	2000
**2g**	0.111	172	6.96	28.034	1	4.55	19.069	500
**2h**	0.022	810	7.67	25.543	1	4.59	17.427	500
**2i**	0.044	1231	7.35	30.353	2	4.52	54.509	500
**2j**	0.695	24	6.16	28.886	1	4.54	16.317	500
**2k**	0.758	22	6.12	39.457	0	4.40	16.568	1000
**2l**	0.147	109	6.83	23.384	1	4.63	16.109	1033
**2m**	0.321	43	6.49	24.265	1	4.62	13.706	948
**2n**	0.159	92	6.80	72.250	0	4.14	14.687	1000
**2o**	0.670	131	6.17	50.799	2	4.29	87.547	1000
**2p**	0.043	1194	7.37	33.225	2	4.48	51.124	500
**2q**	0.029	4250	7.54	81.575	2	4.09	122.724	500
**2r**	0.049	1641	7.31	26.064	3	4.58	80.536	500
**2s**	0.038	2166	7.42	75.763	1	4.12	83.229	1000
**4a**	2.755	22	5.56	**0.318**	**193**	**6.50**	61.551	500
**4b**	0.686	28	6.16	0.503	38	6.30	19.189	500
**4c**	1.762	21	5.75	0.790	47	6.10	36.997	500
**4d**	2.307	17	5.64	1.744	22	5.76	38.379	500
**4e**	2.006	2	5.70	0.522	8	6.28	3.944	2000
**5a**	3.804	3	5.42	1.884	6	5.72	10.769	500
**5b**	1.457	1	5.84	1.962	1	5.71	2.004	500
**5c**	2.314	0	5.64	9.414	0	5.03	0.805	500

^1^ *P. falciparum*, strain NF54, erythrocytic stages; ^2^ SI is defined as the ratio: IC_50_ in L6 cells/IC_50_ in each parasite; ^3^ *T. brucei rhodesiense*, strain STIB900 trypomastigote bloodstream forms; ^4^ cytotoxicity L6 cells rat skeletal myoblasts; ^5^ pIC_50_ = −log_10_ IC_50_ (M); ^6^ The oral toxicity was calculated using the ProTox 3.0 software (https://tox.charite.de/protox3/, accessed on 25 October 2024), and the calculated median lethal dose (LD_50_) is given in mg/kg body weight. Reference drugs: chloroquine (chl.), melarsoprol (mel.), podophyllotoxin (pod.). Values in bold highlight the most striking results.

## Data Availability

The data presented in this study are available on request from corresponding author.

## Supplementary Materials

The following supporting information can be downloaded at: https://www.mdpi.com/article/10.3390/ijms26052114/s1.
