# Supplementary material for: Pharmacomodulation of the Redox-Active Lead Plasmodione: Synthesis of Substituted 2-Benzylnaphthoquinone Derivatives, Antiplasmodial Activities, and Physicochemical Properties
Source: Int J Mol Sci. 2025 Feb 27;26(5):2114. doi: 10.3390/ijms26052114 (PMC11900971; doi:10.3390/ijms26052114)

Pharmacomodulation of the Redox-Active Lead Plasmodione: Synthesis of substituted 2-Benzylnaphthoquinone Derivatives, Antiplasmodial Activities and Physicochemical Properties

Armin Presser [1,*], Gregor Blaser [1], Eva-Maria Pferschy-Wenzig [2], Marcel Kaiser [3,4], Pascal Mäser [3,4] and Wolfgang Schuehly [2]

1 Institute of Pharmaceutical Sciences, Pharmaceutical Chemistry, University of Graz, Schubertstrasse 1, 8010 Graz, Austria
2 Institute of Pharmaceutical Sciences, Pharmacognosy, University of Graz, Universitaetsplatz 4, 8010 Graz, Austria
3 Swiss Tropical and Public Health Institute, Kreuzstrasse 2, 4123 Allschwil, Switzerland
4 Faculty of Philosophy and Natural Sciences, University of Basel, Swiss TPH, Petersplatz 1, 4003 Basel, Switzerland

Supplementary Material

Table of contents

1. Methodology for biological testing **S2**

2. Calculated physicochemical properties **S3-S6**

3. Fully annotated correlation plots **S7-S9**

4. ^{1}H- and ^{13}C-NMR spectra for the target compounds **S10-S36**

1. Methodology for biological testing

1.1 In vitro growth inhibition assay of Plasmodium falciparum NF54

Plasmodium falciparum, strain NF54, erythrocytic stages, and the standard drug, chloroquine, were used for the assay. The parasite cultures incubated in RPMI 1640 medium with 5% AlbuMAX™ (without hypoxanthine) were exposed to serial drug dilutions in microtiter plates. After 48 h of incubation at 37 °C in a reduced oxygen atmosphere, 0.5 μCi [^{3}H]-hypoxanthine was added to each well of the plate. Cultures were incubated for a further 24 h before they were harvested onto glass-fiber filters and washed with distilled water. The radioactivity was counted using a Betaplate™ liquid scintillation counter (Wallac, Zurich). The results were recorded as counts per minute (CPM) per well at each drug concentration and expressed as percentage of the untreated controls. IC_{50} values were calculated from the sigmoidal inhibition curves using Microsoft Excel. Chloroquine was used as control.

1.2 In vitro growth inhibition assay of Trypanosoma brucei rhodesiense

Trypanosoma brucei rhodesiense, STIB 900 strain, and the standard drug, melarsoprol, were used for the assay. Minimum Essential Medium (50 μL) supplemented with 25 mM HEPES, 1g/L additional glucose, 1% MEM non-essential amino acids (100x), 0.2 mM 2-mercaptoethanol, 1 mM Na-pyruvate and 15% heat-inactivated horse serum was added to each well of a 96-well microtiter plate. Serial drug dilutions of 11 three-fold dilution steps covering a range from 100 to 0.002 μg/mL were prepared. Then 4 x 10^{3} bloodstream forms of *T. b. rhodesiense* (STIB 900) in 50 μL were added to each well and the plate incubated at 37 °C under a 5% CO_2 atmosphere for 72 h. 10 μL Alamar Blue (resazurin, 12.5 mg in 100 mL double-distilled water) was then added to each well and incubation continued for a further 2–4 h. Then, the plates were read with a Spectramax Gemini XS microplate fluorometer (Molecular Devices Cooperation, Sunnyvale, CA, USA) using an excitation wavelength of 536 nm and an emission wavelength of 588 nm. The IC_{50} values were calculated from the sigmoidal inhibition curves using the microplate reader software Softmax Pro (Molecular Devices Cooperation, Sunnyvale, CA, USA). Melarsoprol was used as control.

1.3 Cytotoxicity against L6 cells

Assays were performed in 96-well microtiter plates, each well containing 100 μL of RPMI 1640 medium supplemented with 1% L-glutamine (200 mM) and 10% foetal bovine serum, and 4000 L6 cells (a primary cell line derived from rat skeletal myoblasts). Serial drug dilutions of 11 threefold dilution steps covering a range from 100 to 0.002 μg/mL were prepared. After 72 h of incubation, the plates were inspected under an inverted microscope to assure growth of the controls and sterile conditions. 10 μL of Alamar Blue solution was then added to each well and the plates incubated for another 2 h. Then the plates were read with a Spectramax Gemini XS microplate fluorometer (Molecular Devices Cooperation, Sunnyvale, CA, USA) using an excitation wavelength of 536 nm and an emission wavelength of 588 nm. The IC_{50} values were calculated by linear regression from the sigmoidal dose inhibition curves using the microplate reader software Softmax Pro (Molecular Devices Cooperation, Sunnyvale, CA, USA). Podophyllotoxin (Sigma P4405) was used as control.

Table S1: Calculated physicochemical properties of the tested compounds.

compd	Ligand efficiency metrics (*T. brucei rhodesiense*)							Physicochemical parameters				
	LE [1]	LLE [1]	LELP [1]	SILE [2]	LLE_{AT} [2]	nBEI [3]	NSEI [3]	MW [1]	log*P* [4]	log$D_{7.4}$ [4]	TPSA [4]	pKa (basic) [4]
**2a**	0.2882	0.4404	13.0510	1.7106	0.1402	5.50	2.10	262.31	3.97	3.78	34.14	2.20
**2b**	0.2392	-0.4248	19.2700	1.6131	0.0858	5.57	2.09	330.31	4.35	4.06	34.14	2.64
**2c**	0.2244	-0.6214	20.9920	1.5570	0.0759	5.49	2.04	348.30	4.50	4.14	34.14	2.86
**2d**	0.2543	-0.0765	18.5240	1.7644	0.1058	6.03	2.32	348.30	4.47	4.15	34.14	2.82
**2e**	0.2212	-0.6794	21.2940	1.5349	0.0728	5.43	2.02	348.30	4.49	4.11	34.14	3.00
**2f**	0.3320	1.6183	10.4330	2.0390	0.2156	6.40	1.69	297.74	3.36	3.36	47.03	3.88
**2g**	0.2715	1.7122	10.4600	1.7771	0.2120	5.91	0.91	307.31	3.56	3.41	77.28	1.20
**2h**	0.2625	1.6518	11.2020	1.7701	0.2043	5.97	0.92	325.30	3.60	3.55	77.28	1.78
**2i**	0.2695	0.4537	15.0820	1.7636	0.1370	5.88	2.26	316.28	3.96	3.85	34.14	3.22
**2j**	0.2595	1.0739	13.3550	1.7496	0.1713	5.92	1.13	319.36	3.30	3.19	63.24	4.18
**2k**	0.2517	0.9385	13.7660	1.6974	0.1636	5.78	1.10	319.36	2.17	3.12	63.24	4.16
**2l**	0.2353	0.6814	16.7850	1.7230	0.1446	6.06	1.16	373.33	3.83	3.48	63.24	1.99
**2m**	0.2345	0.6653	16.8440	1.7170	0.1438	6.05	1.15	373.33	3.76	3.46	63.24	2.28
**2n**	0.1959	-0.7173	24.8010	1.5080	0.0761	5.60	1.04	401.39	4.32	3.76	63.24	3.32
**2o**	0.2031	-0.5644	23.9170	1.5637	0.0833	5.76	1.07	401.39	4.23	3.70	63.24	3.35
**2p**	0.2926	0.6160	13.2020	1.7967	0.1502	5.80	2.24	280.30	4.01	3.91	34.14	3.02
**2q**	0.2244	-0.7723	21.6650	1.5566	0.0677	5.49	1.36	346.31	4.63	4.19	43.37	2.79
**2r**	0.2515	-0.2767	19.3230	1.7453	0.0948	5.98	1.53	346.31	4.61	4.17	43.37	2.80
**2s**	0.2174	-0.8410	22.8200	1.5505	0.0657	5.54	1.37	364.30	4.85	4.24	43.37	3.13
**4a**	0.4691	3.1758	7.0801	2.6860	0.3390	7.78	3.25	248.28	3.32	3.33	34.14	2.00
**4b**	0.3757	2.1289	11.0990	2.4588	0.2368	7.66	3.15	316.28	3.84	3.86	34.14	3.11
**4c**	0.3488	1.8319	12.2430	2.3520	0.2299	7.48	3.05	334.27	4.06	4.00	34.14	3.39
**4d**	0.3292	1.4878	12.9740	2.2194	0.2183	7.14	2.88	334.27	4.01	3.96	34.14	3.32
**4e**	0.4309	3.2586	7.0171	2.5576	0.3332	7.58	2.09	283.71	2.65	2.74	47.03	3.45
**5a**	0.3415	1.4788	12.4350	2.2348	0.1981	7.09	2.86	316.28	4.07	4.01	34.14	2.46
**5b**	0.2900	0.6130	17.5670	2.1233	0.1412	7.14	2.85	384.28	4.78	4.31	34.14	2.64
**5c**	0.2463	-0.1689	21.0960	1.8497	0.1017	6.47	2.51	402.27	5.07	4.48	34.14	3.96

[1] The ligand efficiency (LE), lipophilic ligand efficiency (LLE) and ligand efficiency lipophilic price (LELP) are based on IC_{50} data in nmol/L, these values and the molecular weight (MW) were calculated using the DataWarrior software, version 6.04.01 (https://openmolecules.org/datawarrior/index.html); [2] The size-independent ligand efficiency (SILE) and the Astex lipophilic ligand efficiency (LLE_{AT}) were calculated according to Ref. [57]; [3] The binding- efficiency index (nBEI) and the surface-binding efficiency index (NSEI) were calculated according to Ref. [55]; [4] The log*P*, log$D_{7.4}$, topological polar surface area (TPSA) and pKa (basic) were calculated using the ADMETlab 3.0 software (https://admetlab3.scbdd.com), the pKa (basic) refers to the acidic dissociation of a conjugated acid from a given base.

Table S2. Calculated physicochemical properties of the tested compounds (continued).

compd	Ligand efficiency metrics (*P. falciparum*)							Multi-parameter scores			
	LE [1]	LLE [1]	LELP [1]	SILE [2]	LLE_{AT} [2]	nBEI [3]	NSEI [3]	PFI [2]	AB-MPS [4]	CNS-MPO [5]	QED [6]
**2a**	0.4421	2.6840	8.5080	2.6240	0.2939	7.75	3.22	5.8	4.8	4.3	0.83
**2b**	0.4181	2.7048	11.0250	2.8193	0.2644	8.70	3.66	6.1	6.1	4.0	0.80
**2c**	0.4357	3.2293	10.8120	3.0230	0.2870	9.34	3.97	6.1	6.1	4.0	0.73
**2d**	0.4224	2.9860	11.1530	2.9304	0.2736	9.09	3.85	6.2	6.2	4.0	0.73
**2e**	0.4522	3.5303	10.4170	3.1376	0.3035	9.64	4.12	6.1	6.1	4.0	0.73
**2f**	0.4856	3.9683	7.1346	2.9817	0.3689	8.75	2.48	5.4	4.4	5.1	0.80
**2g**	0.4149	4.1160	6.8451	2.7155	0.3552	8.32	1.39	5.4	5.4	5.0	0.64
**2h**	0.4383	4.7263	6.7103	2.9551	0.3798	9.05	1.53	5.5	5.5	4.9	0.64
**2i**	0.4386	3.2899	9.2652	2.8708	0.3060	8.72	3.68	5.8	4.8	4.3	0.78
**2j**	0.3520	2.6925	9.8450	2.3734	0.2637	7.54	1.54	5.2	5.2	5.1	0.94
**2k**	0.3499	2.6551	9.9052	2.3590	0.2616	7.50	1.53	5.1	5.1	5.3	0.94
**2l**	0.3471	2.8820	11.3780	2.5417	0.2562	8.26	1.71	5.5	6.5	4.6	0.88
**2m**	0.3299	2.5432	11.9720	2.4156	0.2390	7.92	1.62	5.5	6.5	4.6	0.88
**2n**	0.3216	1.9389	15.1090	2.4753	0.2016	8.26	1.70	5.8	8.8	4.0	0.78
**2o**	0.2921	1.3153	16.6350	2.2482	0.1721	7.64	1.54	5.7	8.7	4.1	0.78
**2p**	0.4814	3.5060	8.0241	2.9560	0.3387	8.69	3.68	5.9	4.9	4.2	0.84
**2q**	0.4137	2.6787	11.7490	2.8705	0.2568	8.94	2.51	6.2	7.2	4.2	0.82
**2r**	0.4011	2.4483	12.1190	2.7828	0.2428	8.71	2.44	6.2	7.2	4.2	0.82
**2s**	0.3913	2.4538	12.6810	2.7902	0.2377	8.83	2.47	6.2	7.2	4.0	0.74
**4a**	0.4015	2.2384	8.2738	2.2985	0.2714	6.84	2.78	5.3	4.3	4.9	0.82
**4b**	0.3676	1.9938	11.3420	2.4061	0.2288	7.53	3.08	5.9	5.9	4.4	0.83
**4c**	0.3289	1.4834	12.9840	2.2177	0.2086	7.13	2.88	6.0	6.0	4.2	0.76
**4d**	0.3222	1.3664	13.2540	2.1726	0.1985	7.02	2.82	6.0	6.0	4.2	0.76
**4e**	0.3908	2.6738	7.7373	2.3195	0.2932	7.00	1.90	4.7	4.3	5.6	0.80
**5a**	0.3233	1.1738	13.1340	2.1158	0.1799	6.78	2.71	6.0	6.0	4.2	0.83
**5b**	0.2966	0.7422	17.1780	2.1714	0.1477	7.27	2.92	6.3	7.3	3.6	0.67
**5c**	0.2761	0.4405	18.8150	2.0739	0.1316	7.08	2.82	6.5	7.5	3.4	0.64

[1] The ligand efficiency (LE), lipophilic ligand efficiency (LLE) and ligand efficiency lipophilic price (LELP) are based on IC_{50} values in nmol/L and were calculated using the DataWarrior software, version 6.04.01 (https://openmolecules.org/datawarrior/index.html); [2] The size-independent ligand efficiency (SILE), Astex lipophilic ligand efficiency (LLE_{AT}) and the Property Forecast Index (PFI) were calculated according to Ref. [57]; [3] The binding- efficiency index (nBEI) and the surface-binding efficiency index (NSEI) were calculated according to Ref. [55]; [4] The AbbVie Multi-Parameter Score (Abb-MPS) were calculated according to Ref. [60]; [5] The central nervous system multiparameter optimization (CNS MPO) was calculated according to Ref [62]; [6] The quantitative estimate of drug-likeness (QED) was calculated using the ADMETlab 3.0 software (https://admetlab3.scbdd.com).

Figure S1. Scatterplot of LLE vs SILE for the synthesized compounds including some optimization trajectories

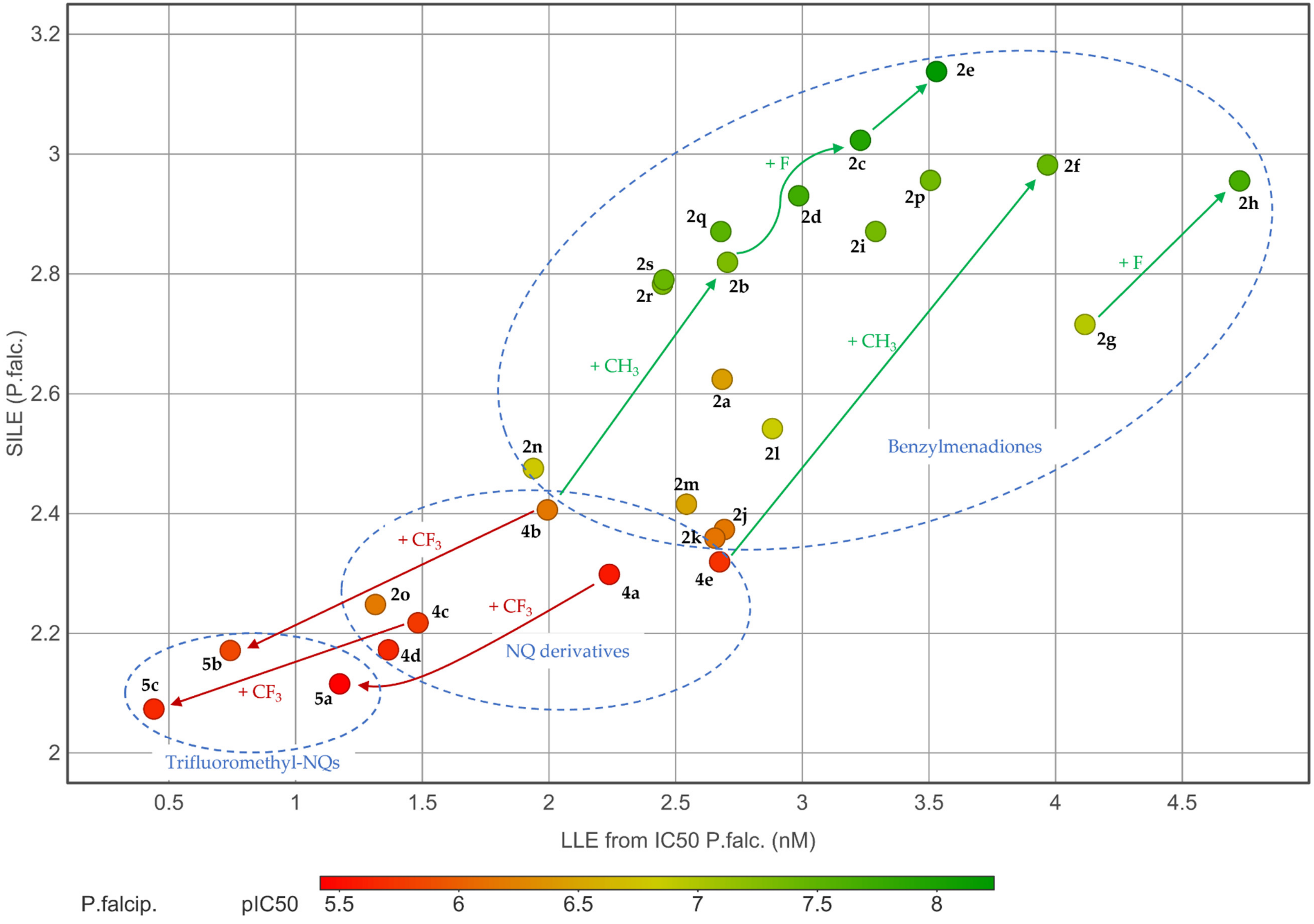

Figure S2. Schematic NSEI-*n*BEI plane analysis of the synthesised benzyl-naphthoquinones including optimization trajectories

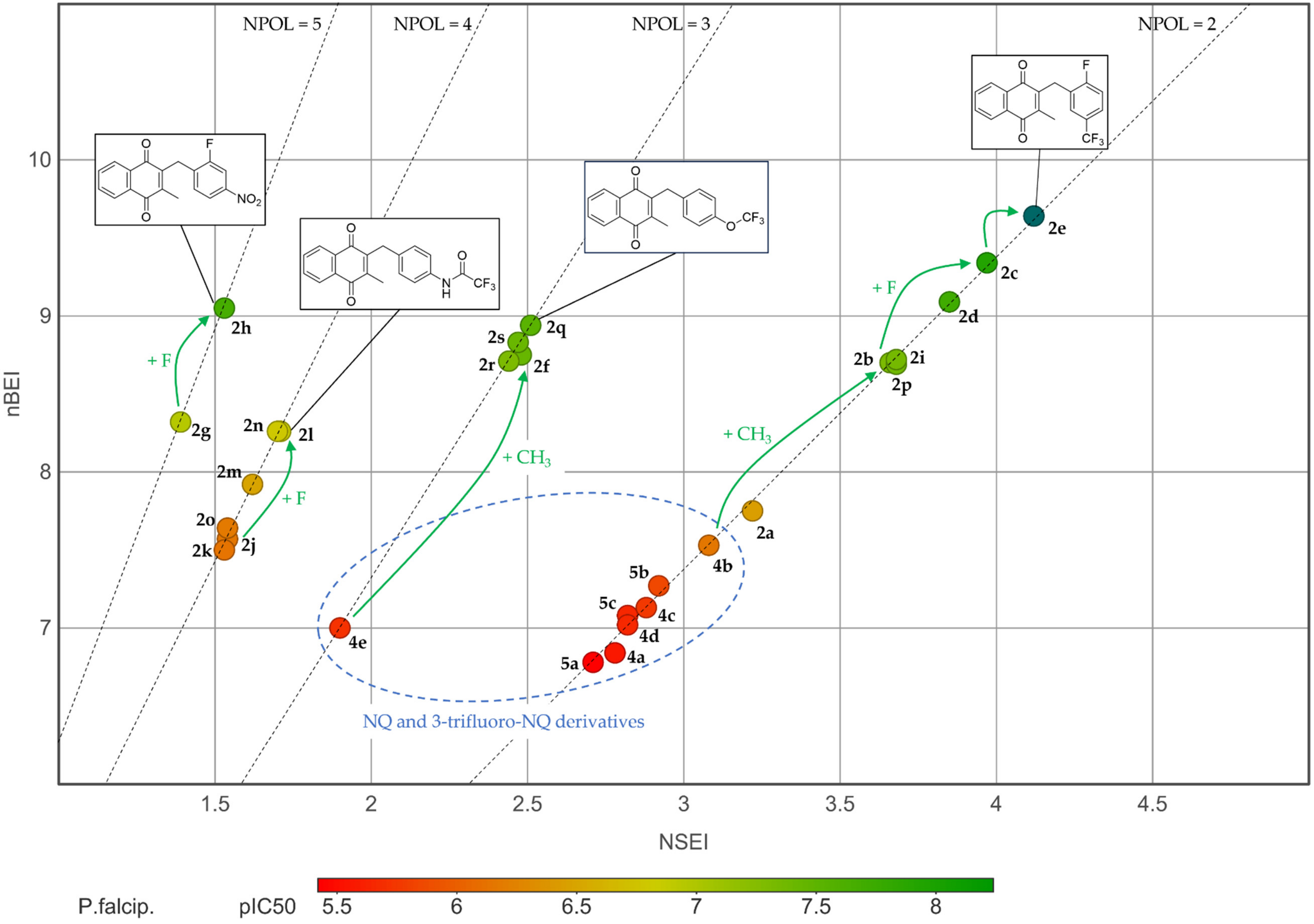

Figure S3. Scatterplot of SILE vs calculated log SI (*P. falciparum*) for the synthesized compounds

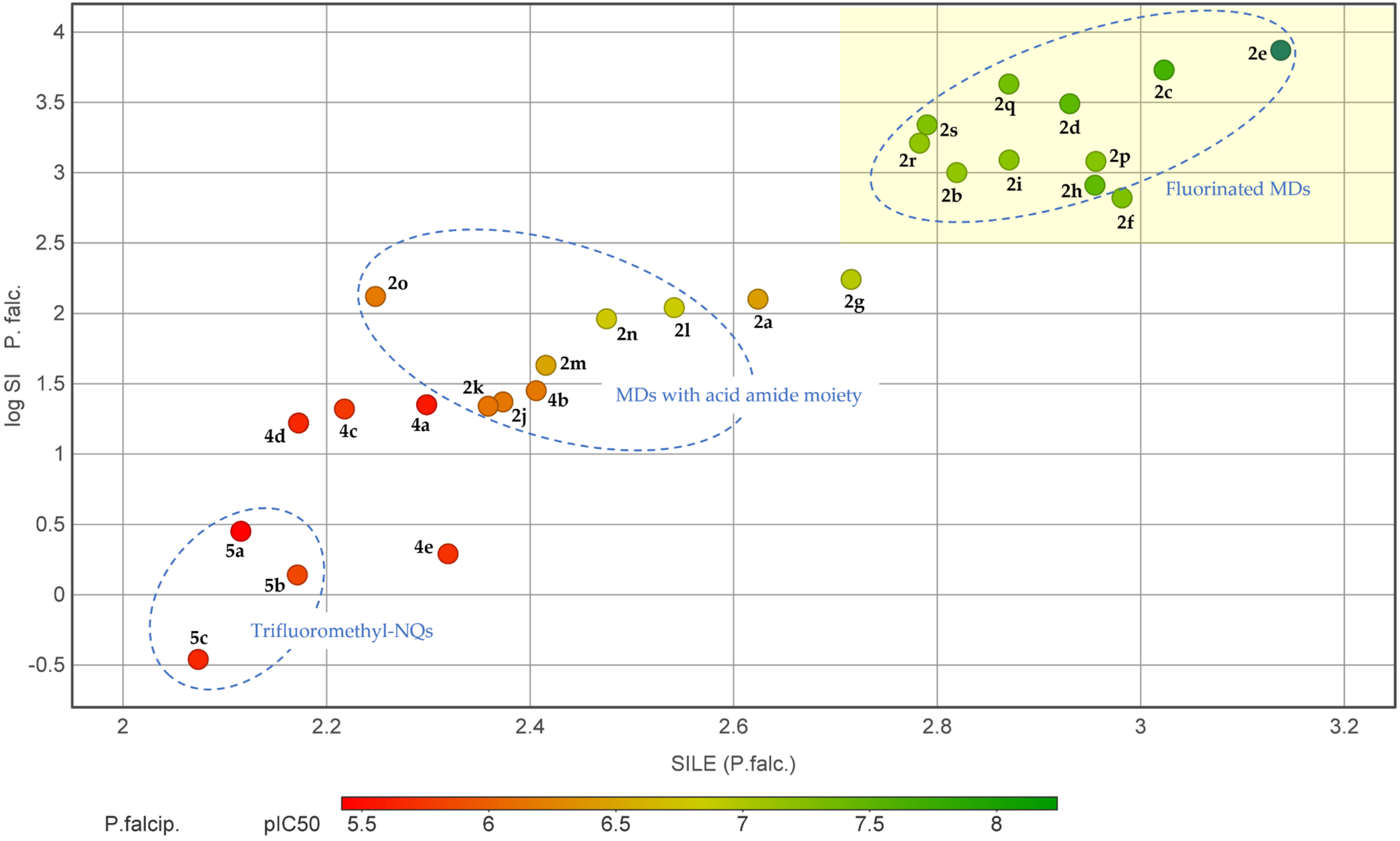

^{1}H- and ^{13}C-NMR spectra for the target compounds

2-Benzyl-3-methyl-1,4-naphthoquinone (**2a**)

^{1}H NMR

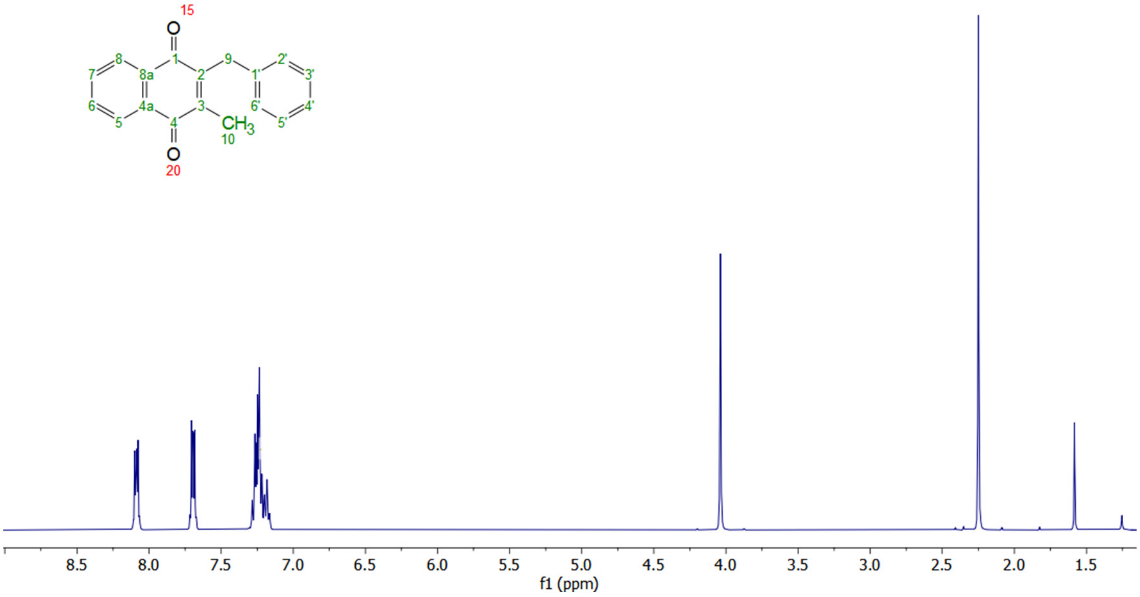

^{13}C NMR

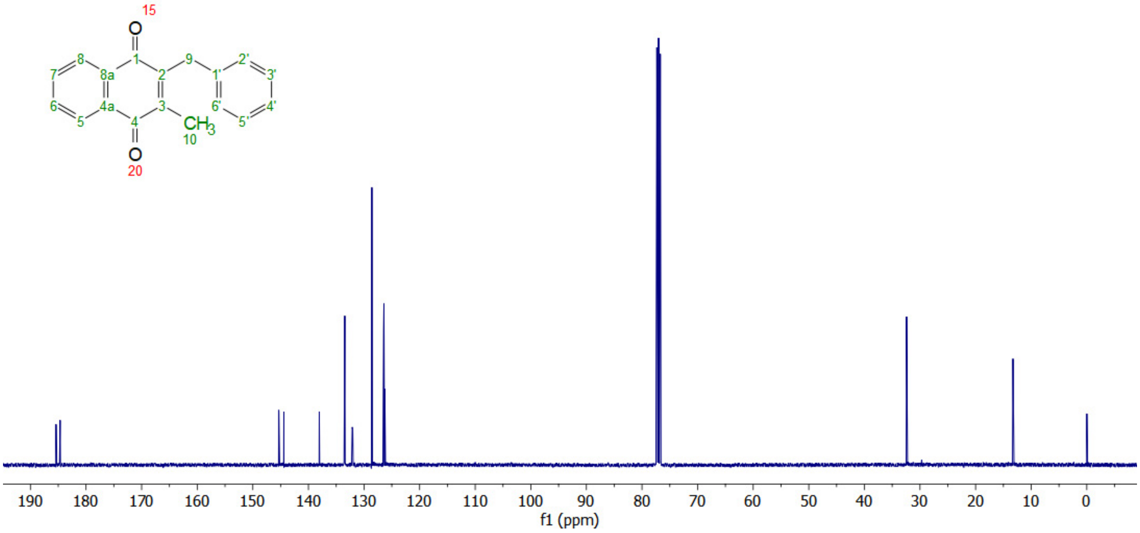

2-Methyl-3-{[4-(trifluoromethyl)phenyl]methyl}-1,4-naphthoquinone (**2b**).

^{1}H NMR

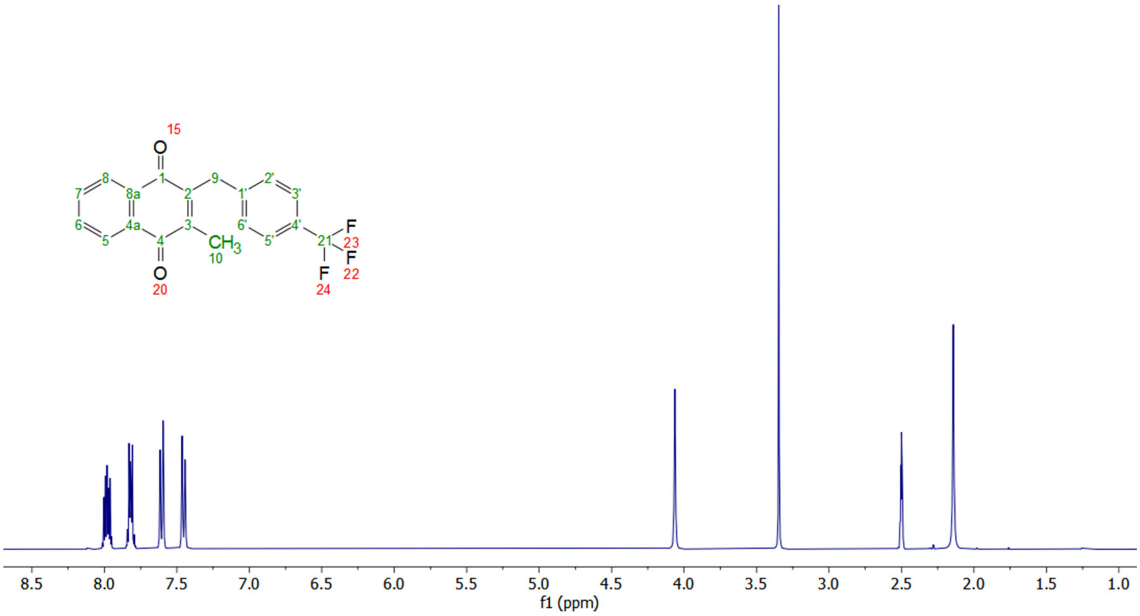

^{13}C NMR

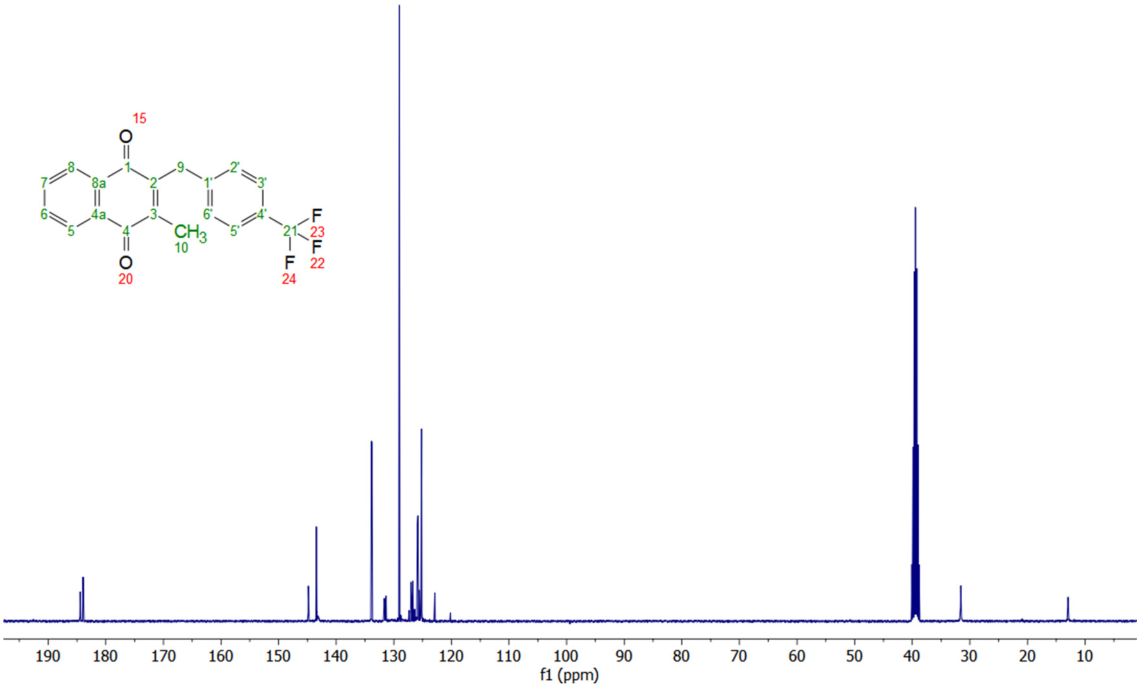

2-{[2-Fluoro-4-(trifluoromethyl)phenyl]methyl}-3-methyl-1,4-naphthoquinone (**2c**).

^{1}H NMR

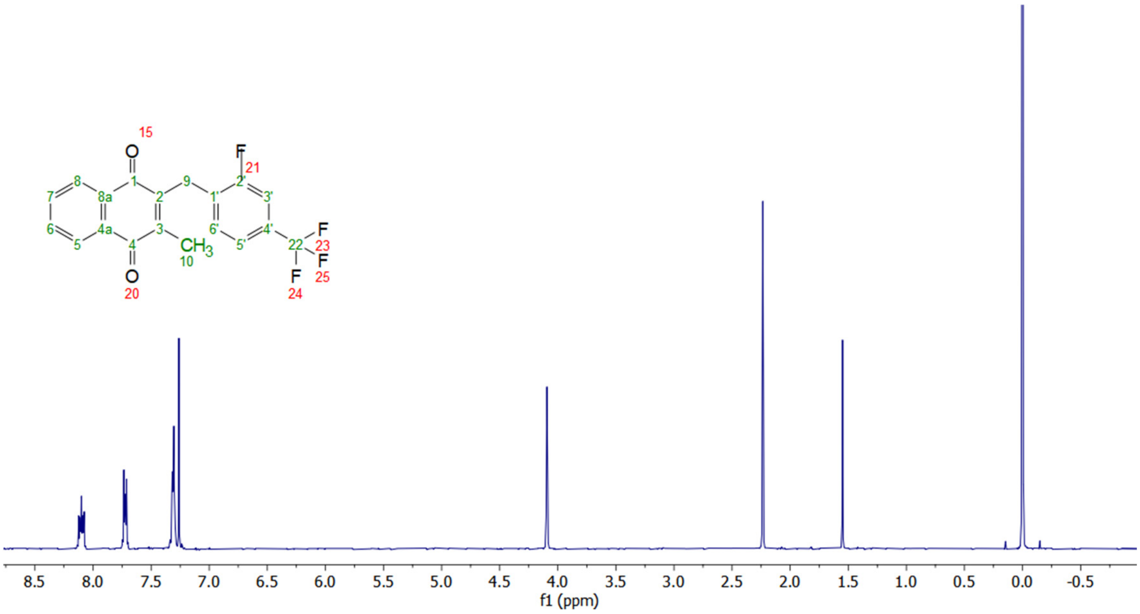

^{13}C NMR

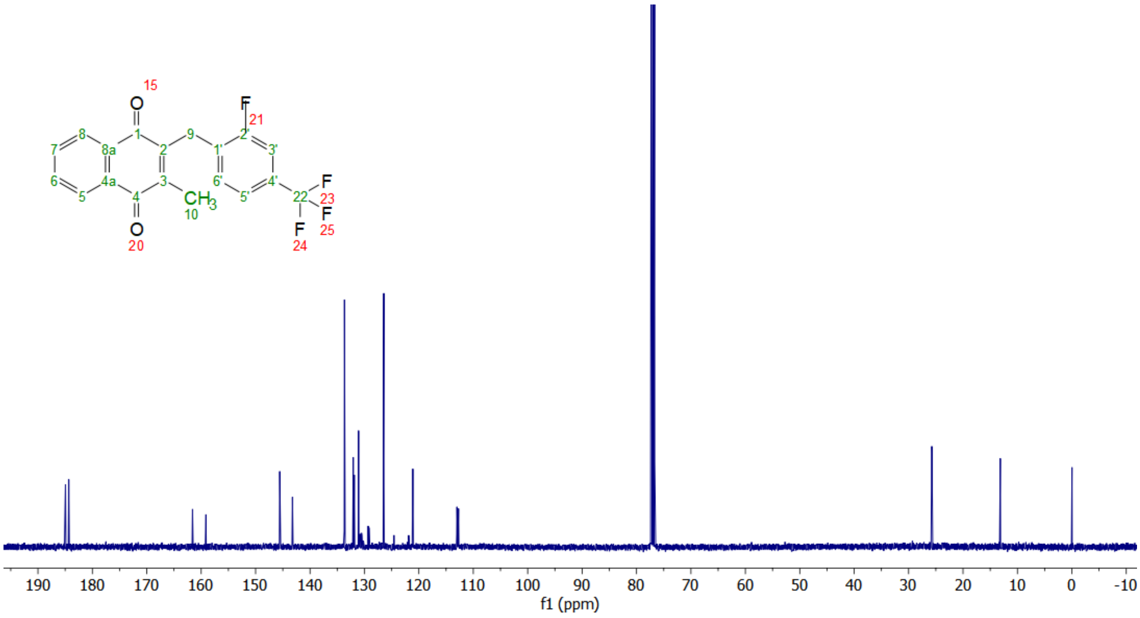

2-{[4-Fluoro-2-(trifluoromethyl)phenyl]methyl}-3-methyl-1,4-naphthoquinone (**2d**)

^{1}H NMR

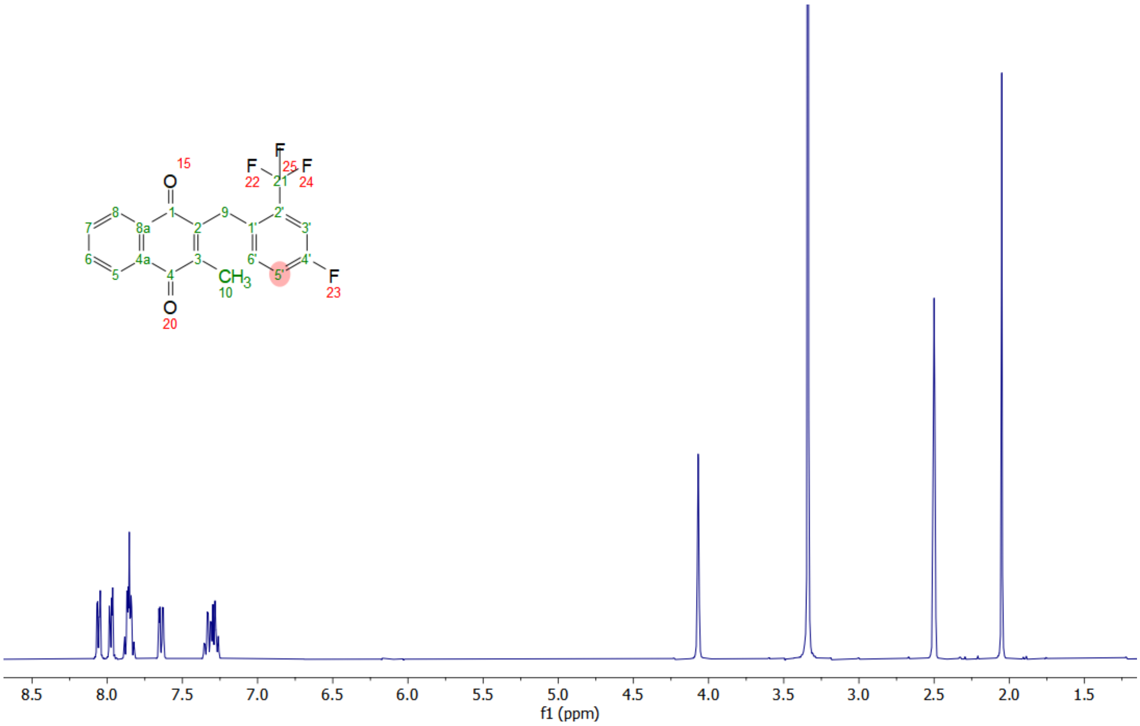

^{13}C NMR

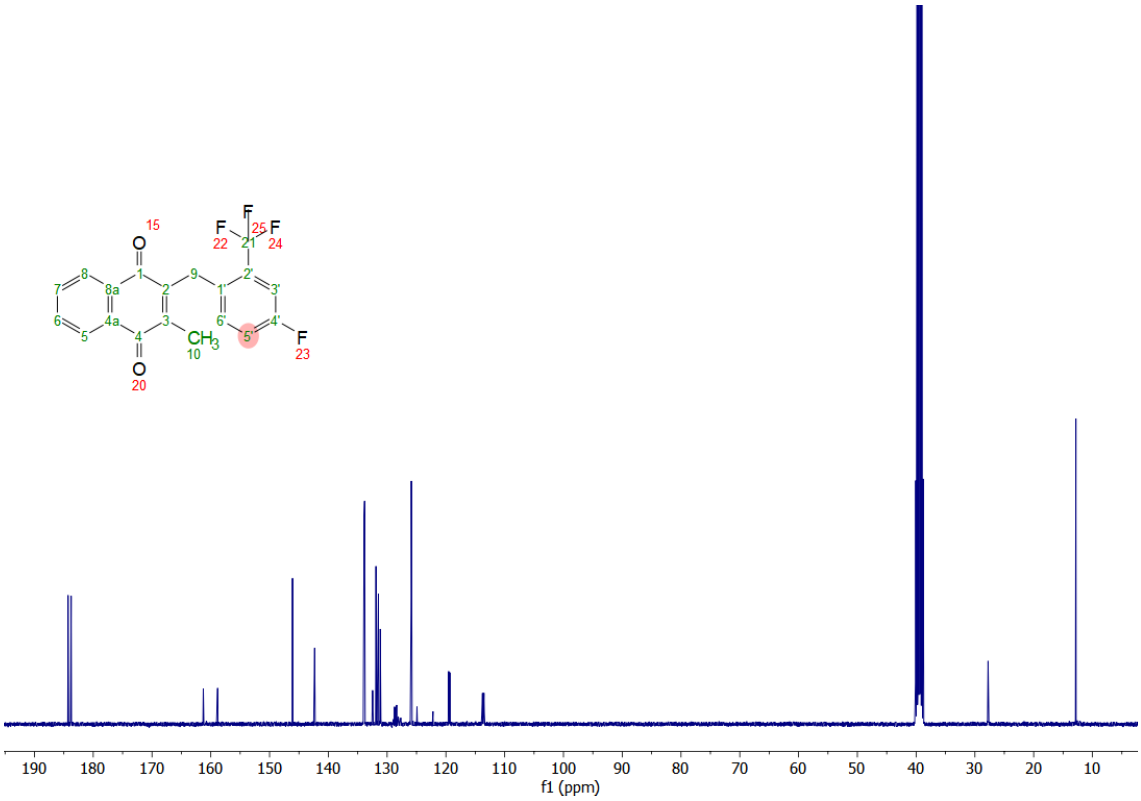

2-{[2-Fluoro-5-(trifluoromethyl)phenyl]methyl}-3-methyl-1,4-naphthoquinone (**2e**)

^{1}H NMR

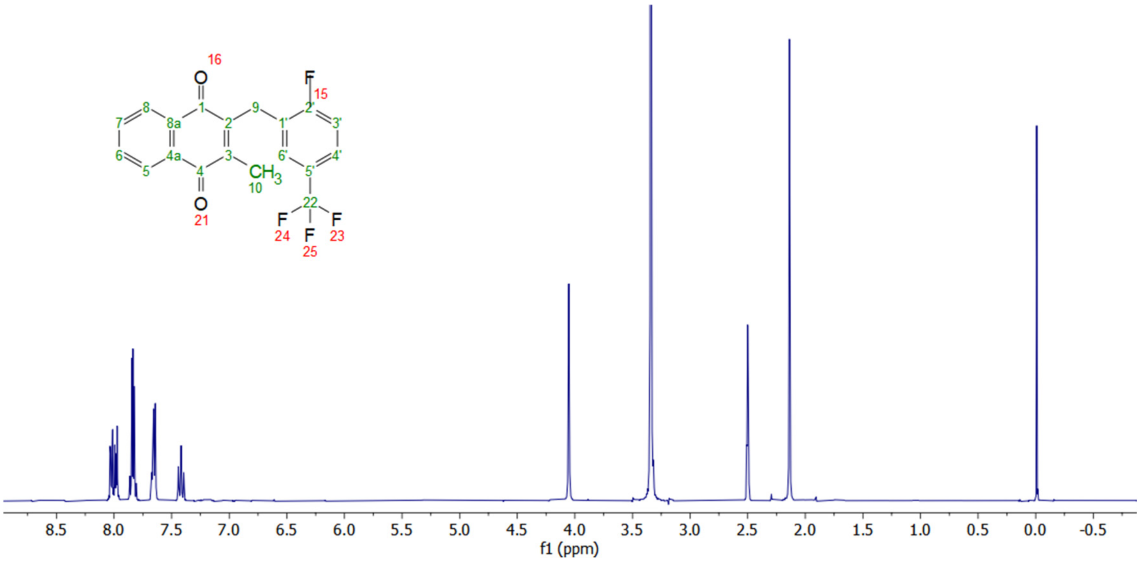

^{13}C NMR

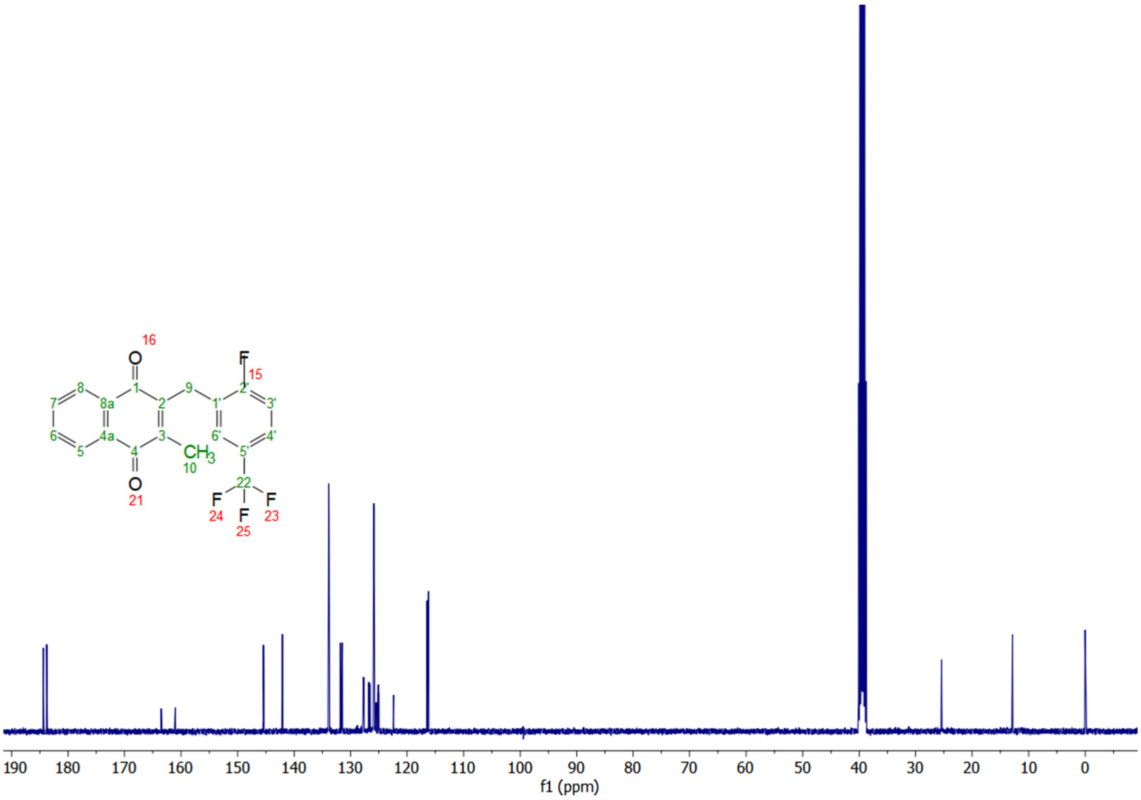

2-[(6-Chloropyridin-3-yl)methyl]-3-methyl-1,4-naphthoquinone (**2f**)

^{1}H NMR

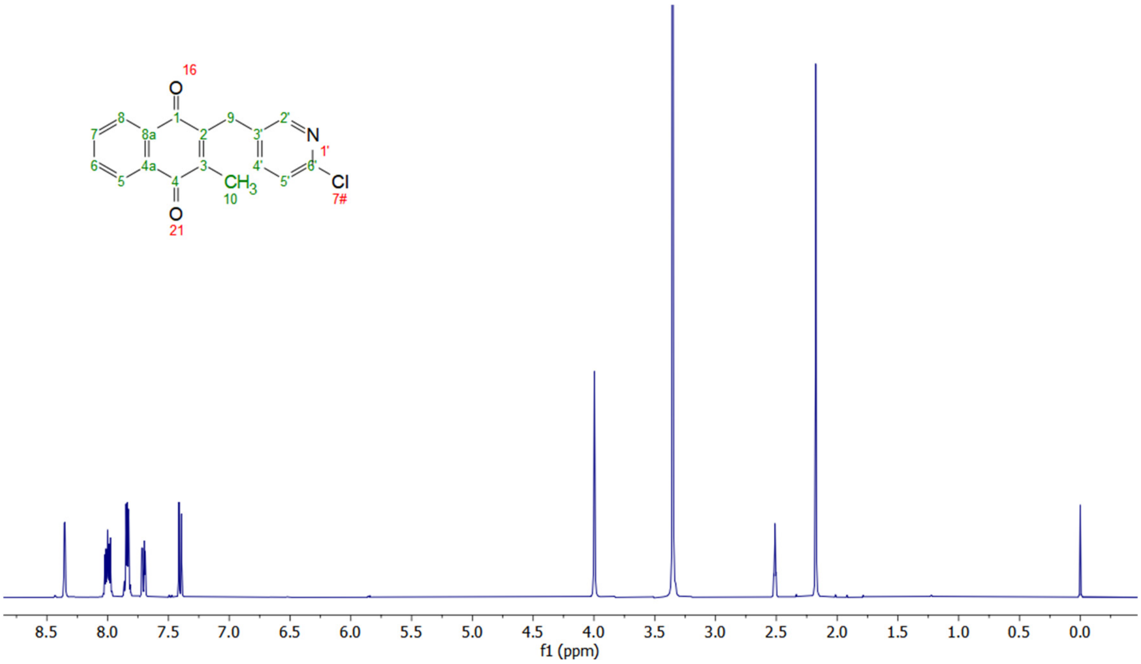

^{13}C NMR

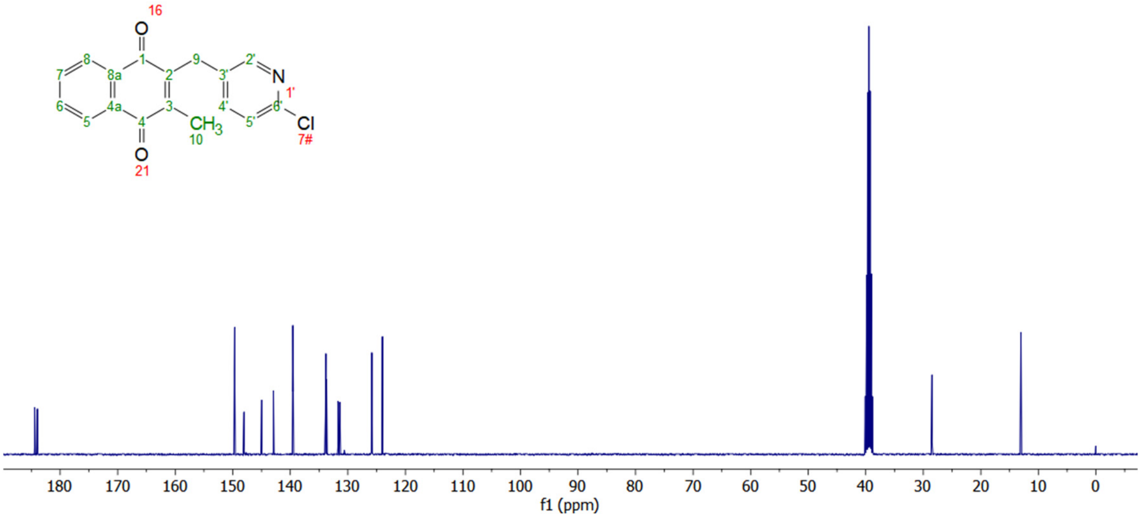

2-Methyl-3-[(4-nitrophenyl)methyl]-1,4-naphthoquinone (**2g**)

^{1}H NMR

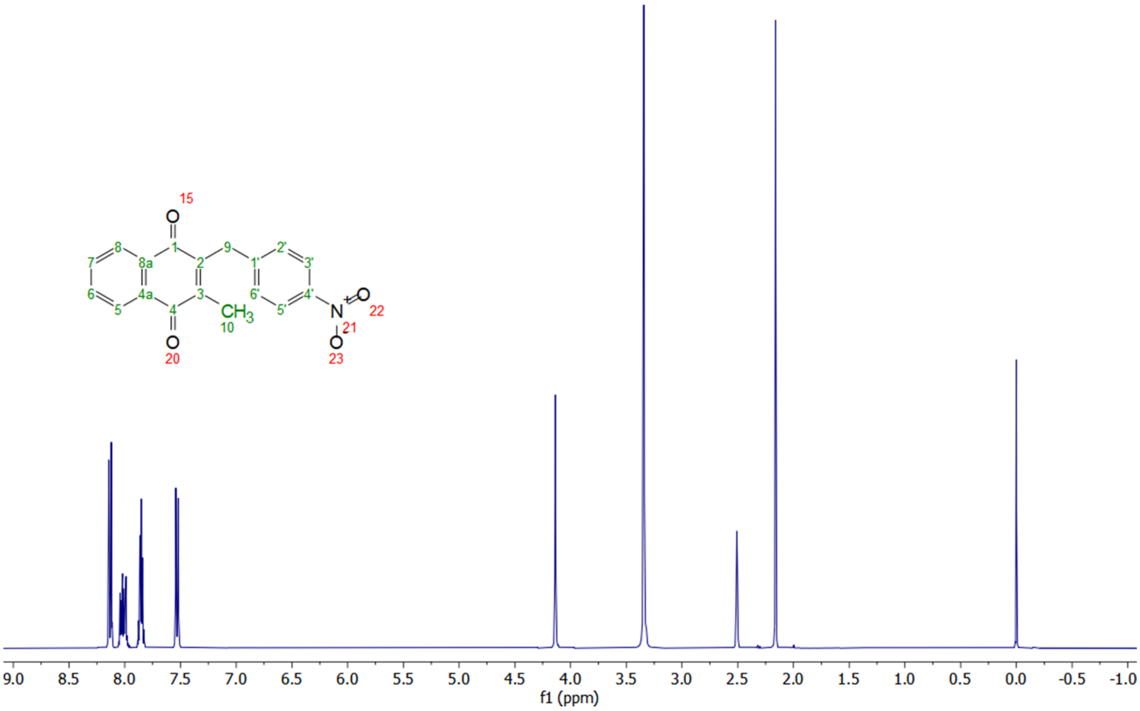

^{13}C NMR

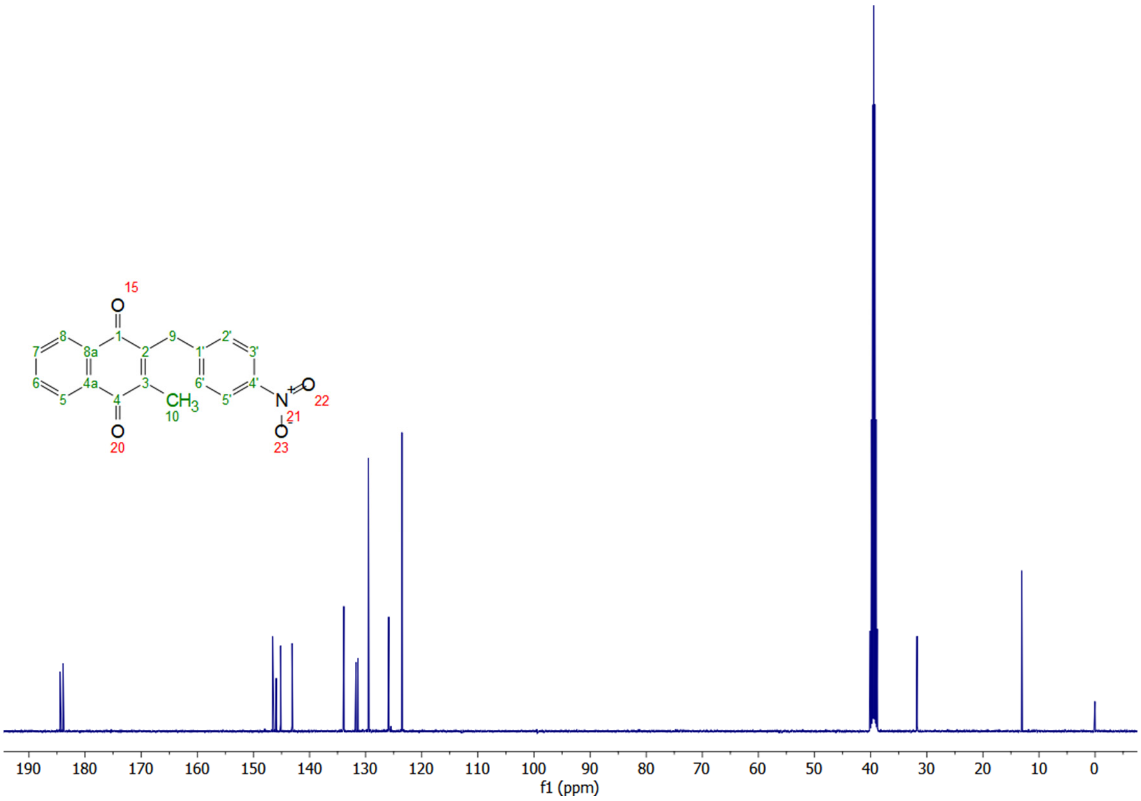

2-[(2-Fluoro-4-nitrophenyl)methyl]-3-methyl-1,4-naphthoquinone (**2h**)

^{1}H NMR

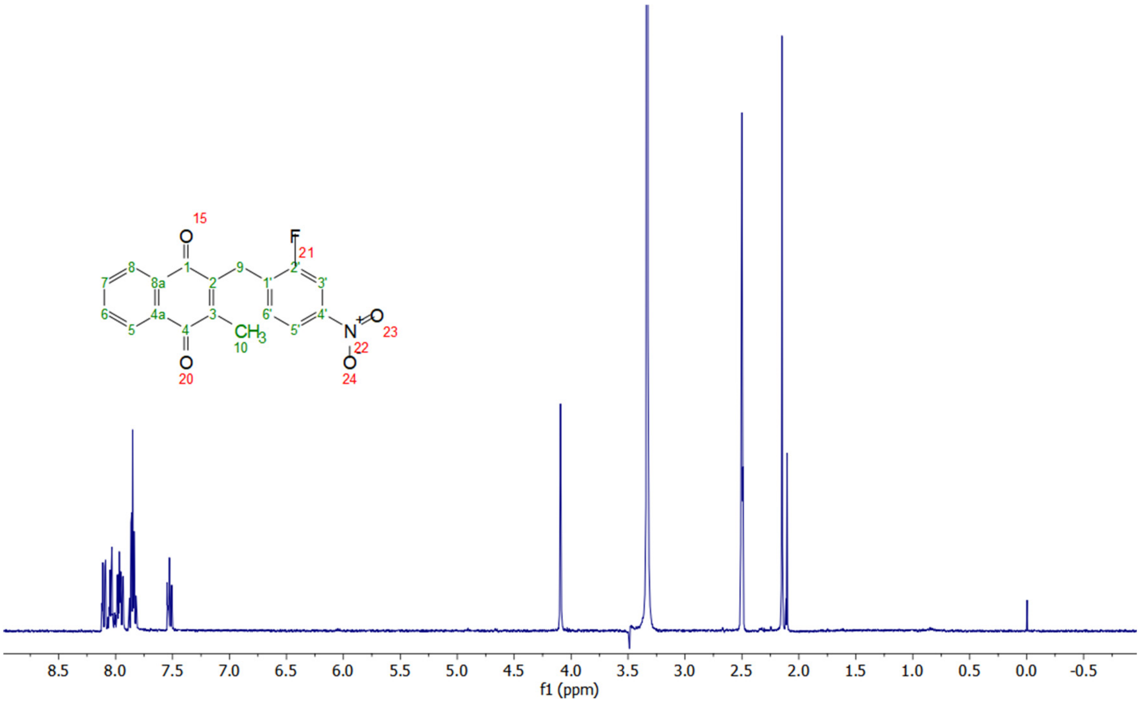

^{13}C NMR

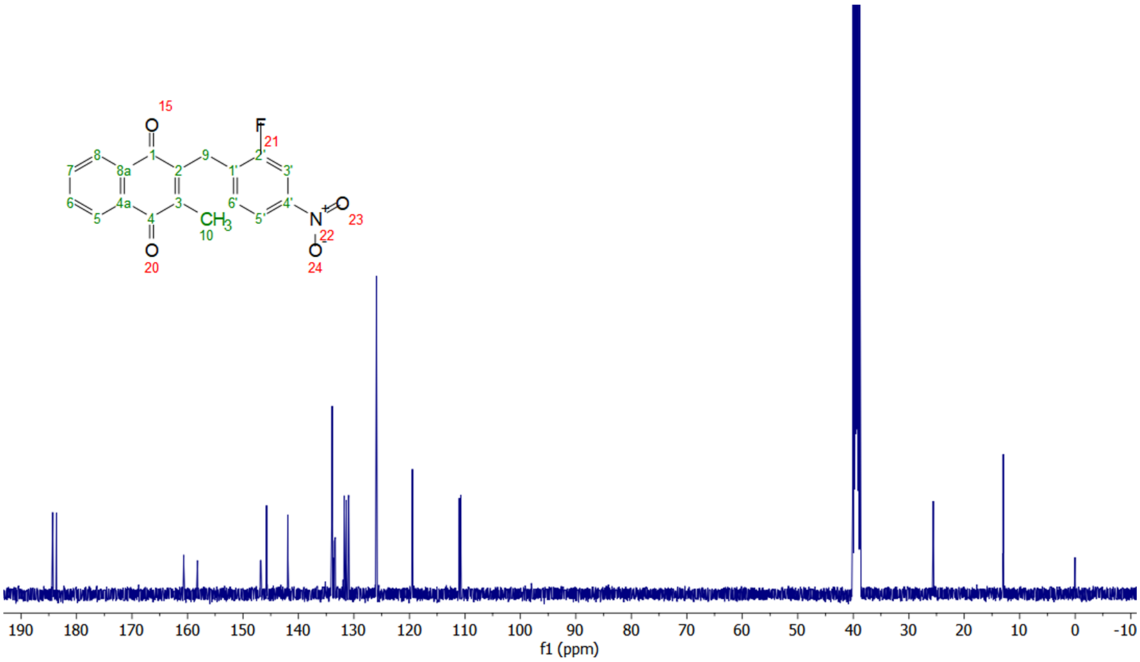

2-Methyl-3-[(2,4,5-trifluorophenyl)methyl]-1,4-naphthoquinone (**2i**)

^{1}H NMR

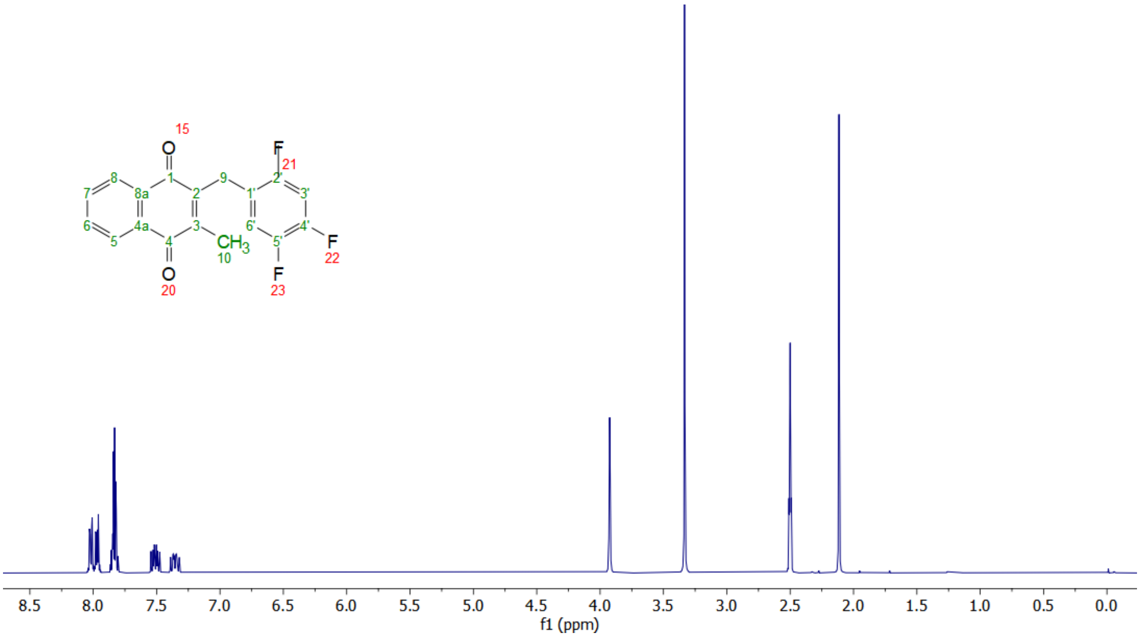

^{13}C NMR

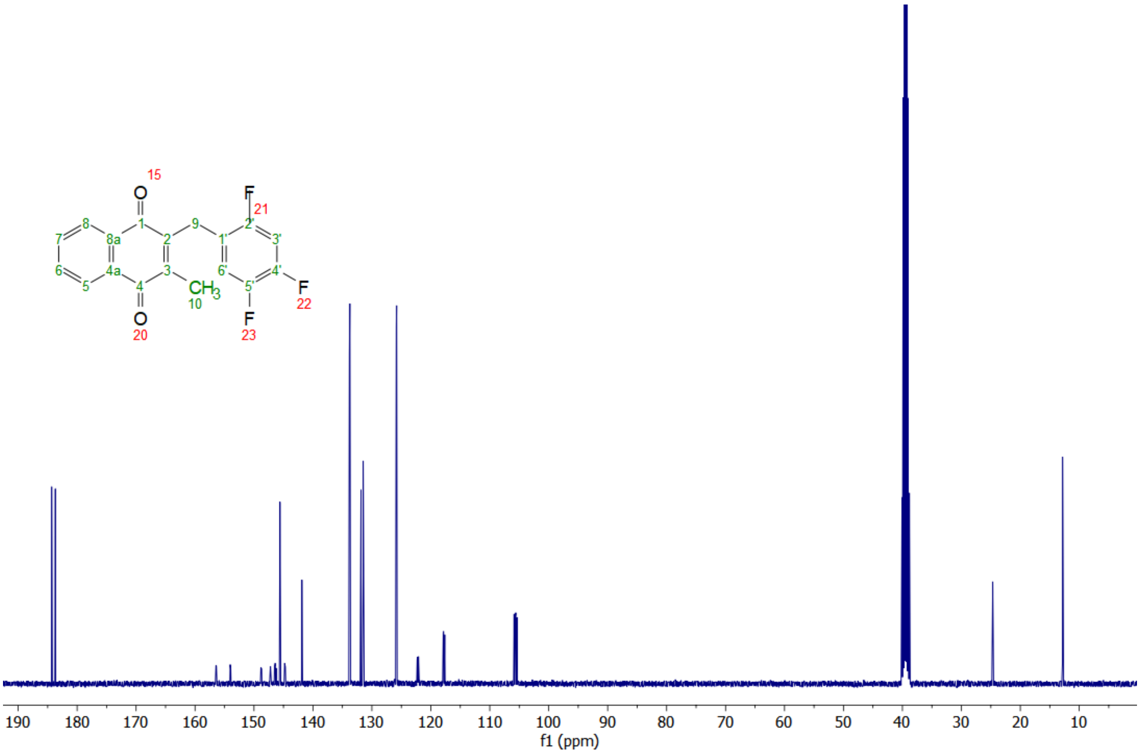

2-[(4-Acetamidophenyl)methyl]-3-methyl-1,4-naphthoquinone (**2j**)

^{1}H NMR

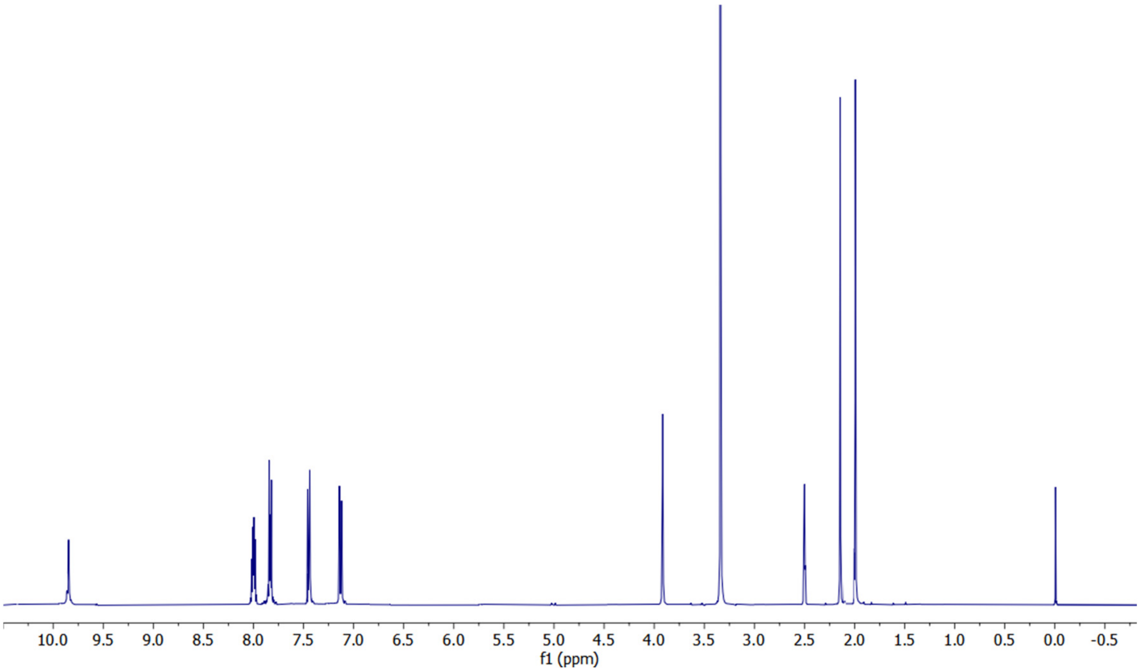

^{13}C NMR

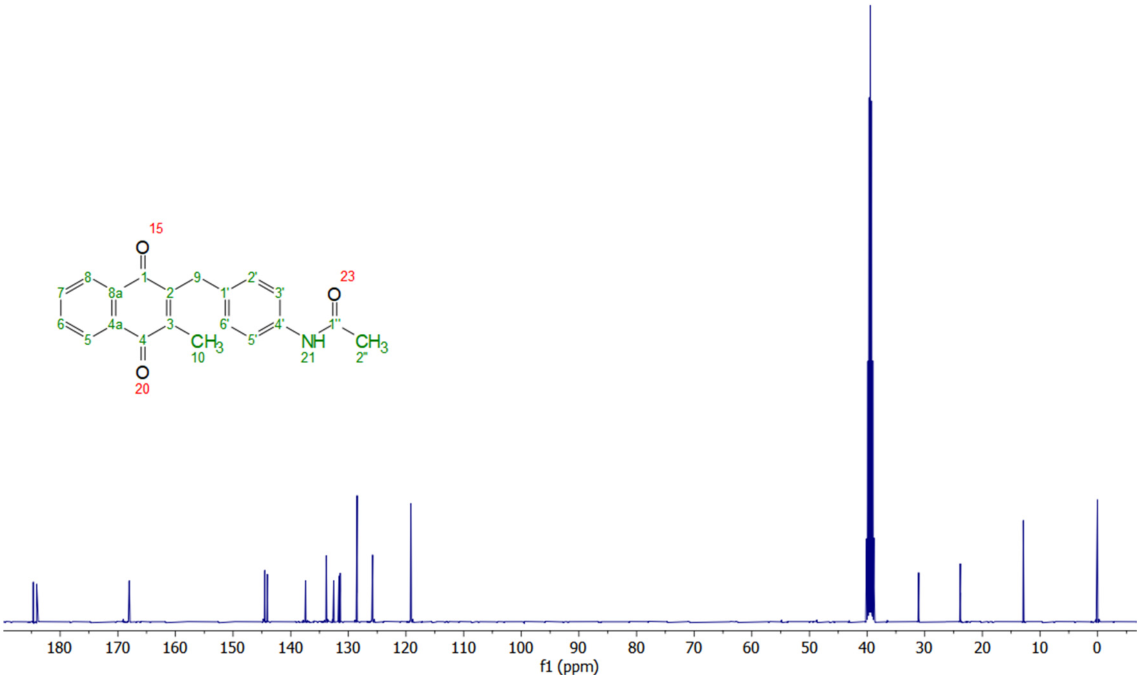

2-[(3-Acetamidophenyl)methyl]-3-methyl-1,4-naphthoquinone (**2k**)

^{1}H NMR

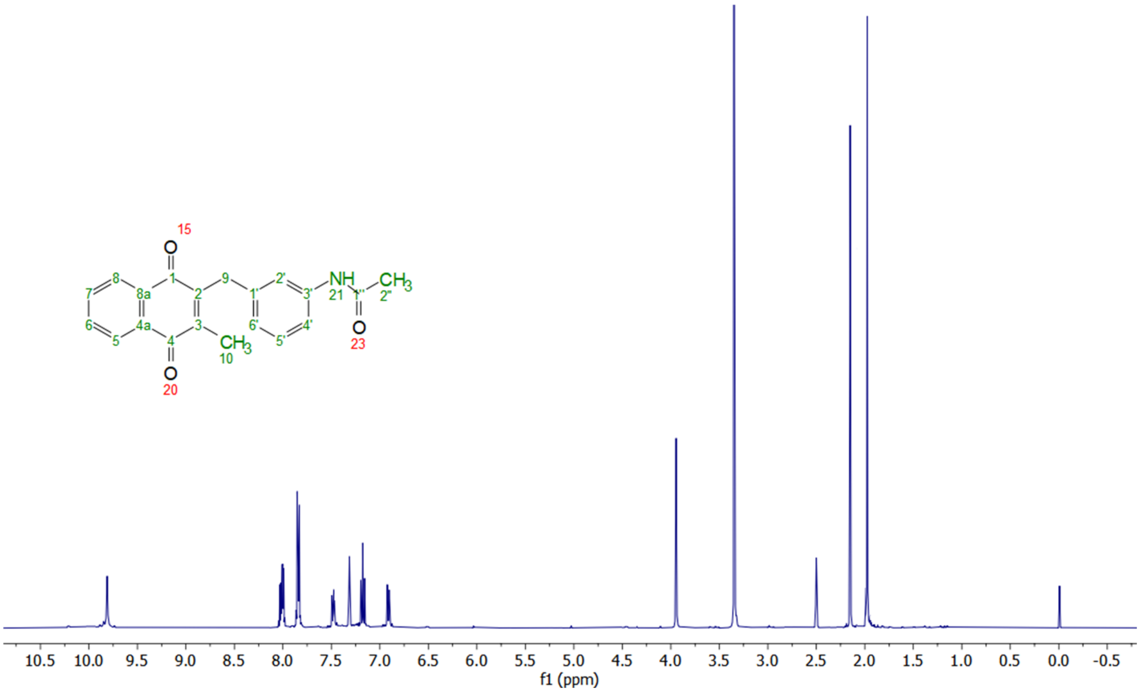

^{13}C NMR

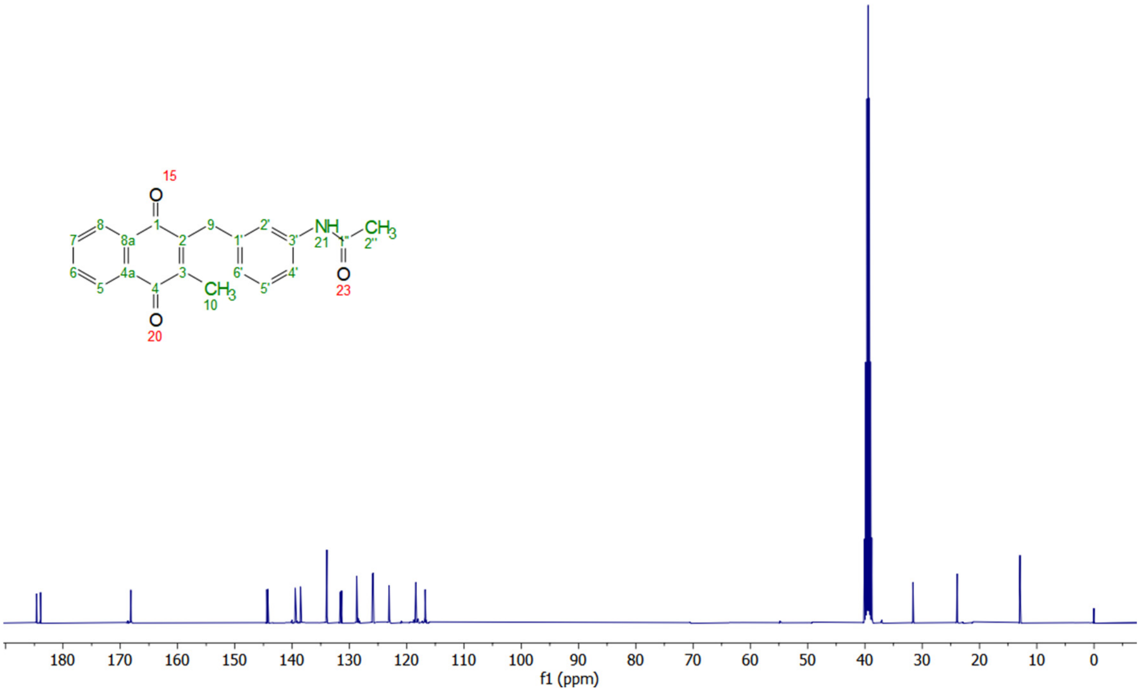

2-Methyl-3-{[4-(2,2,2-trifluoroacetamido)phenyl]methyl}-1,4-naphthoquinone (**2l**)

^{1}H NMR

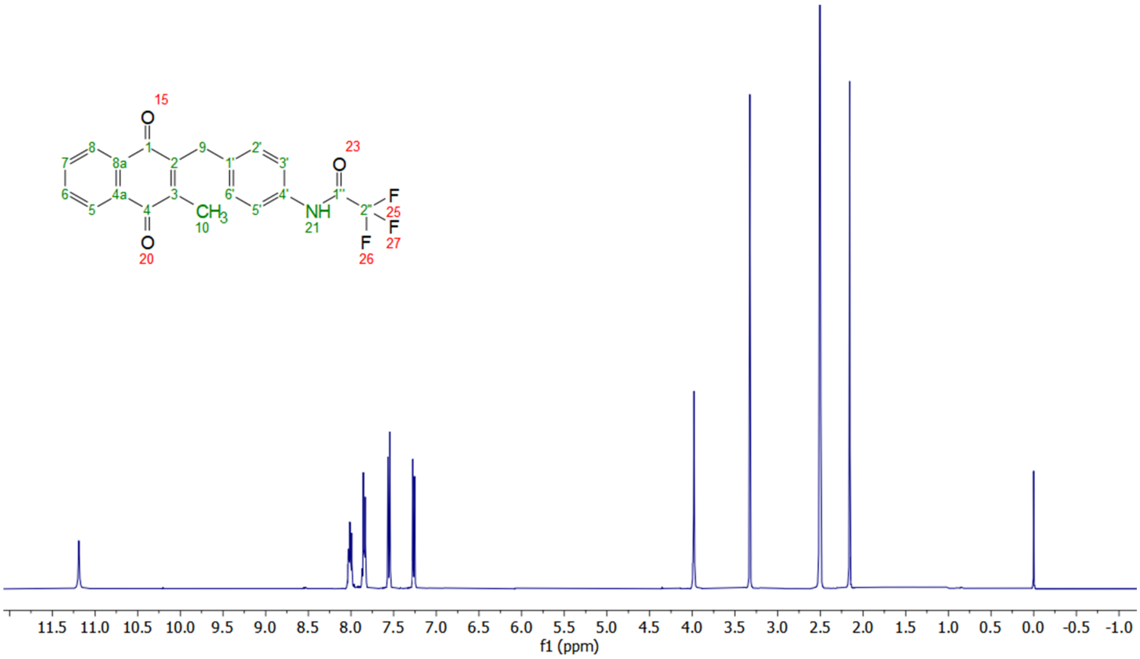

^{13}C NMR

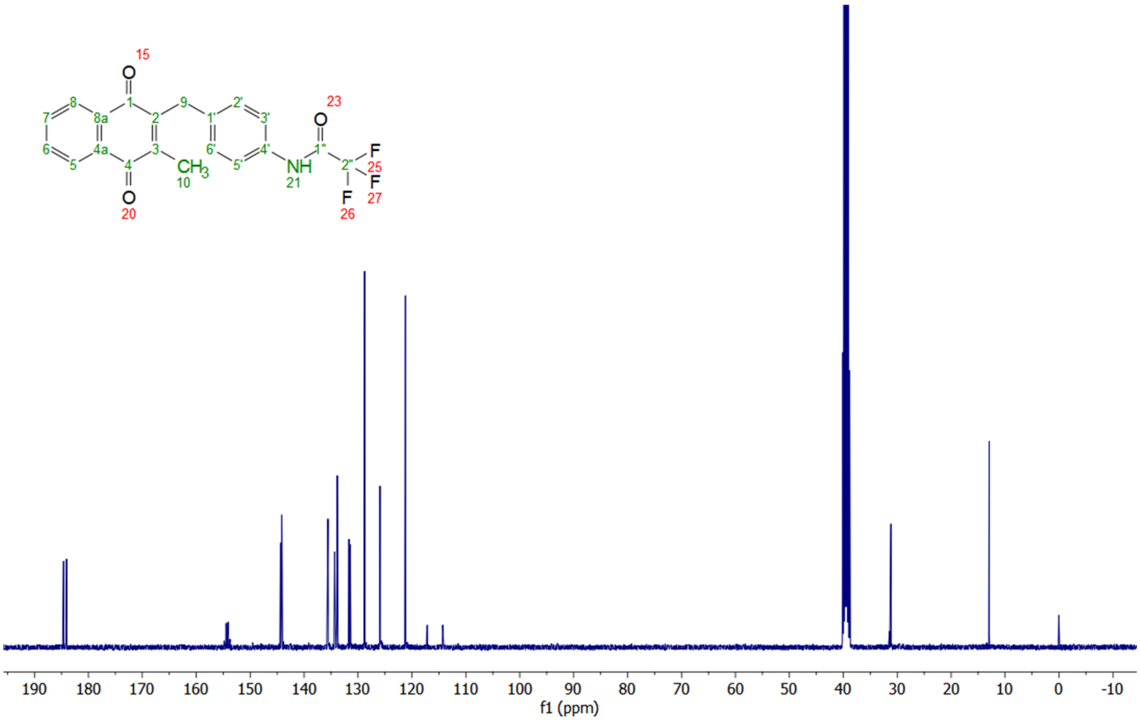

2-Methyl-3-{[3-(2,2,2-trifluoroacetamido)phenyl]methyl}-1,4-naphthoquinone (**2m**)

^{1}H NMR

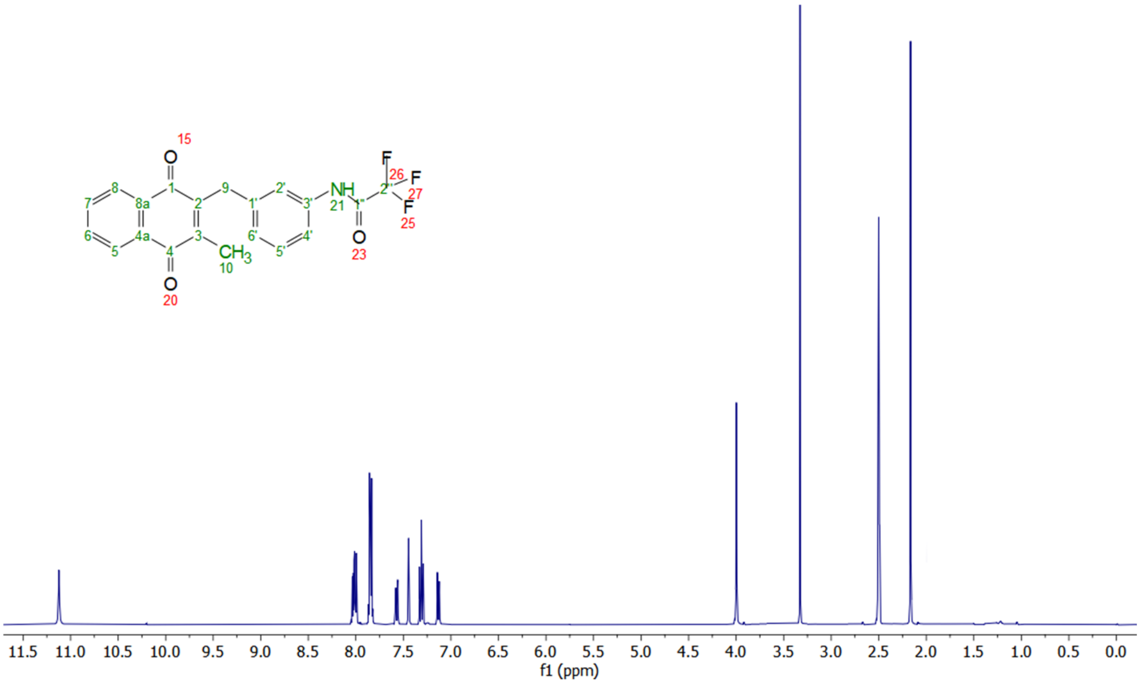

^{13}C NMR

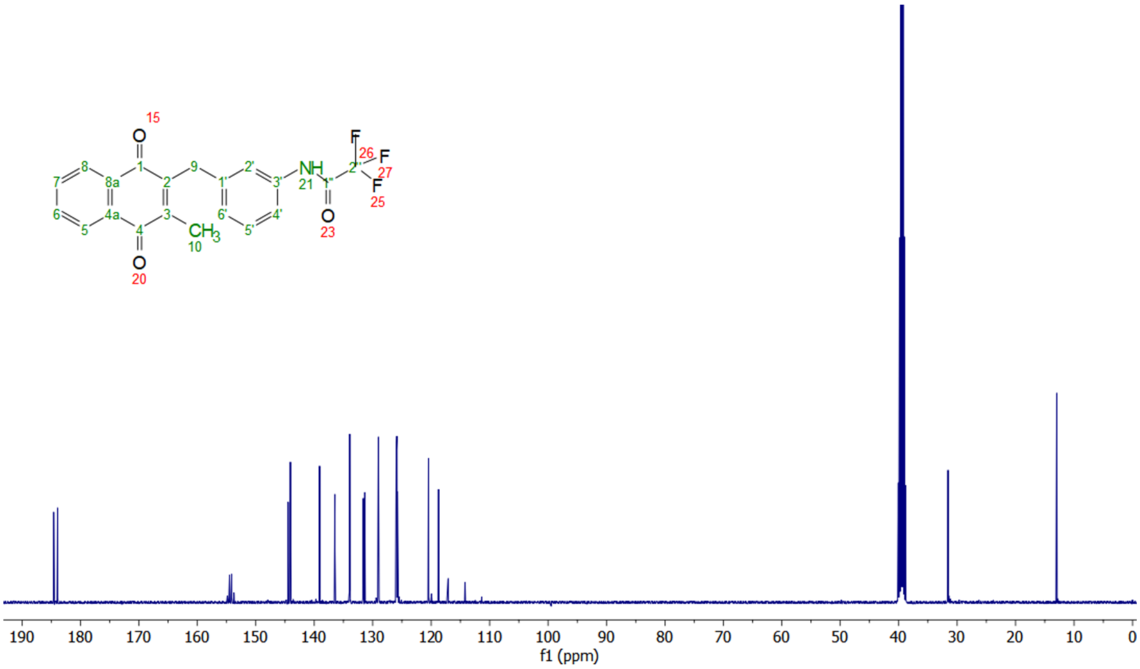

2-Methyl-2-{[4-(4,4,4-trifluorobutanamido)phenyl]methyl}-1,4-naphthoquinone (**2n**)

^{1}H NMR

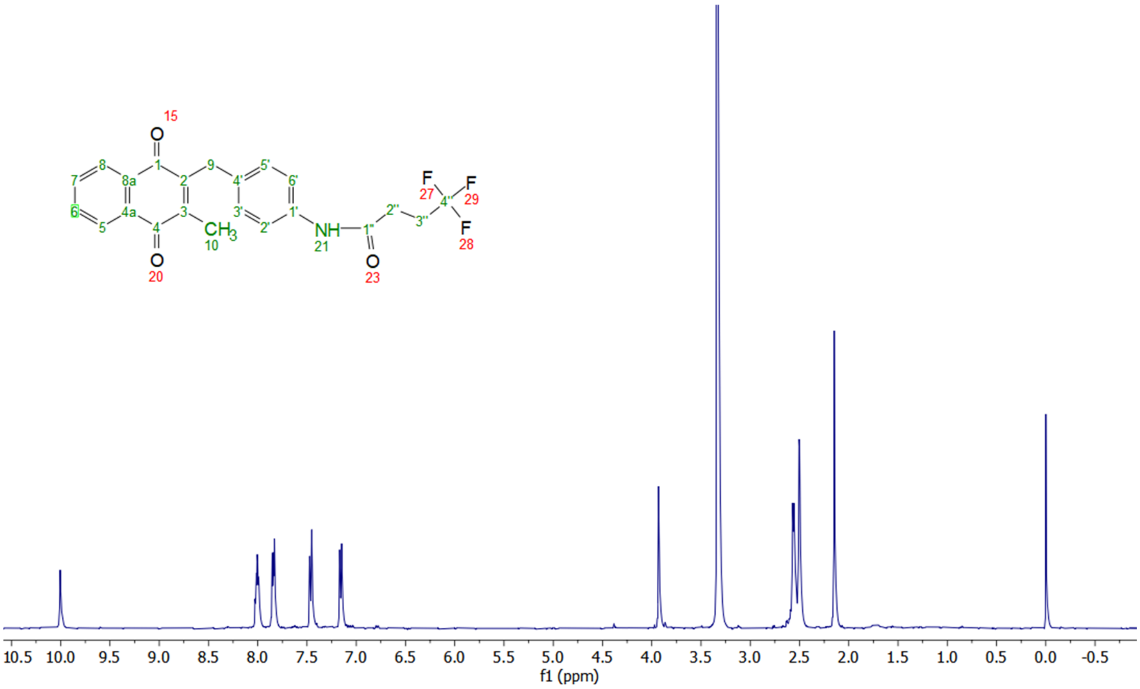

^{13}C NMR

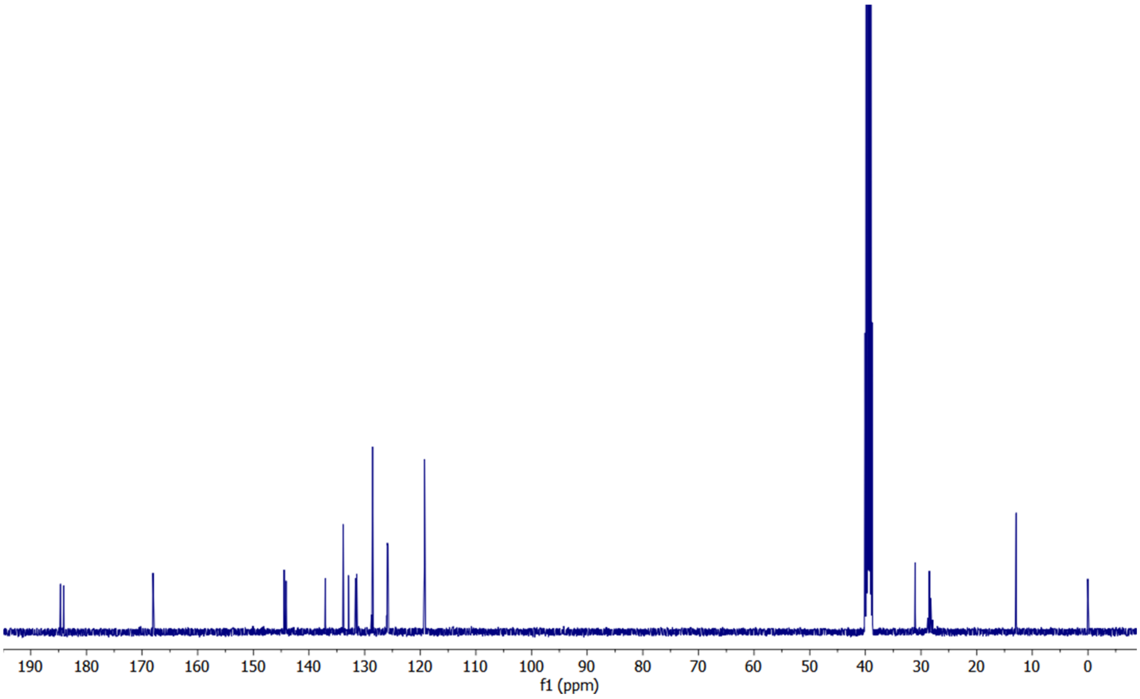

2-Methyl-3-{[3-(4,4,4-trifluorobutanamido)phenyl]methyl}-1,4-naphthoquinone (**2o**)

^{1}H NMR

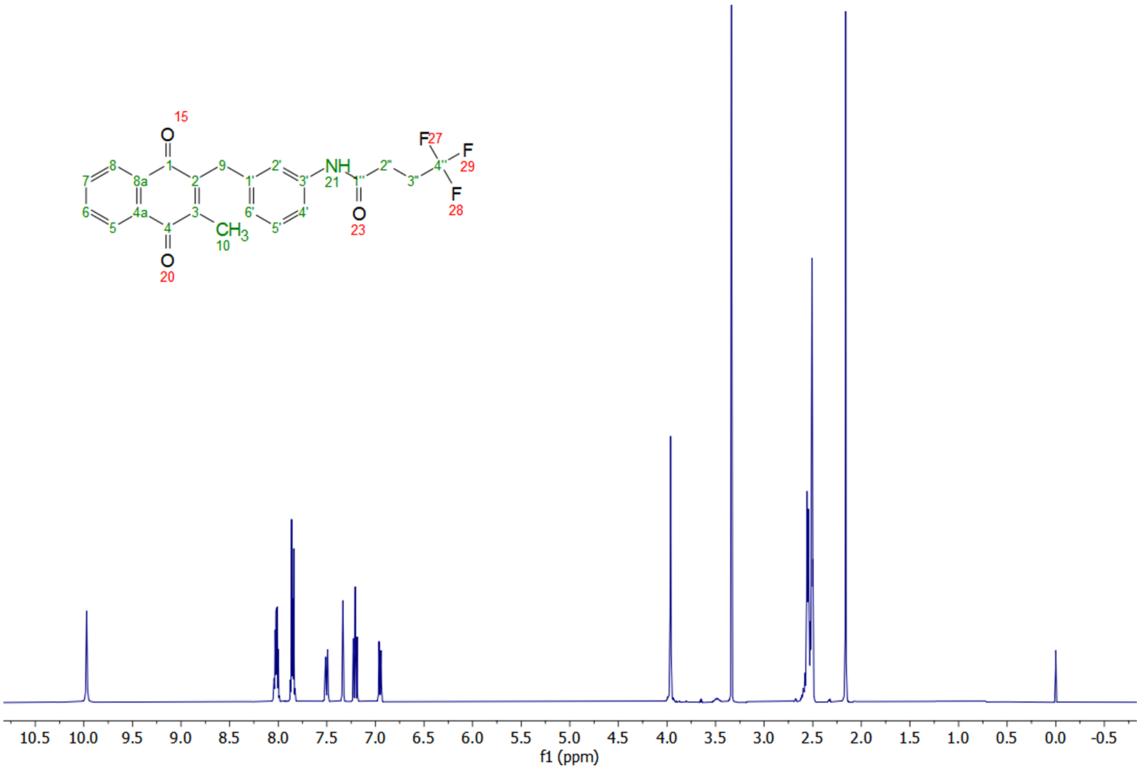

^{13}C NMR

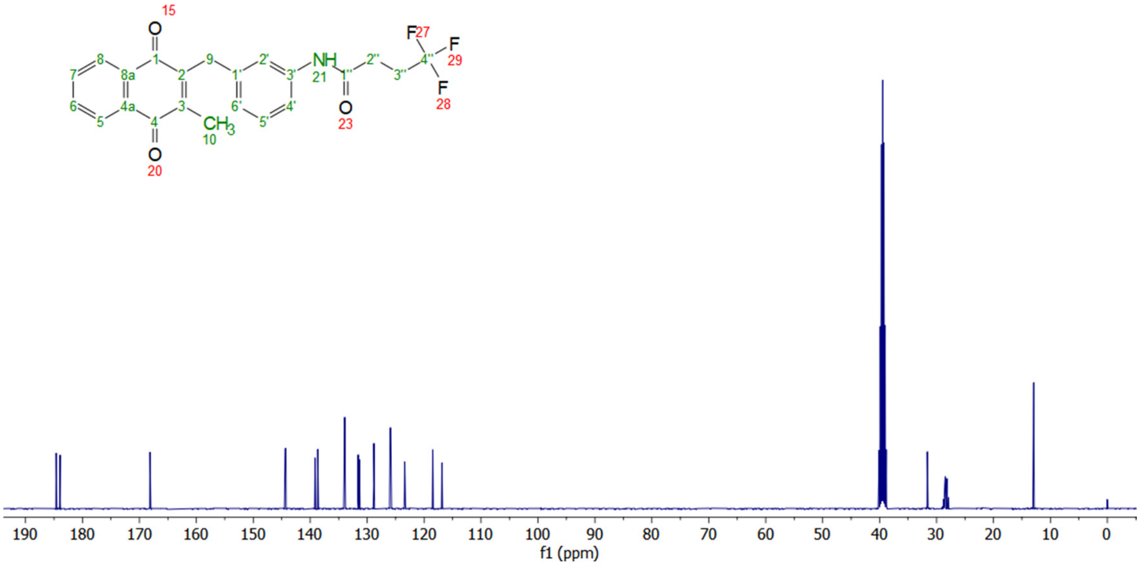

2-[(4-Fluorophenyl)methyl]-3-methyl-1,4-naphthoquinone (**2p**)

^{1}H NMR

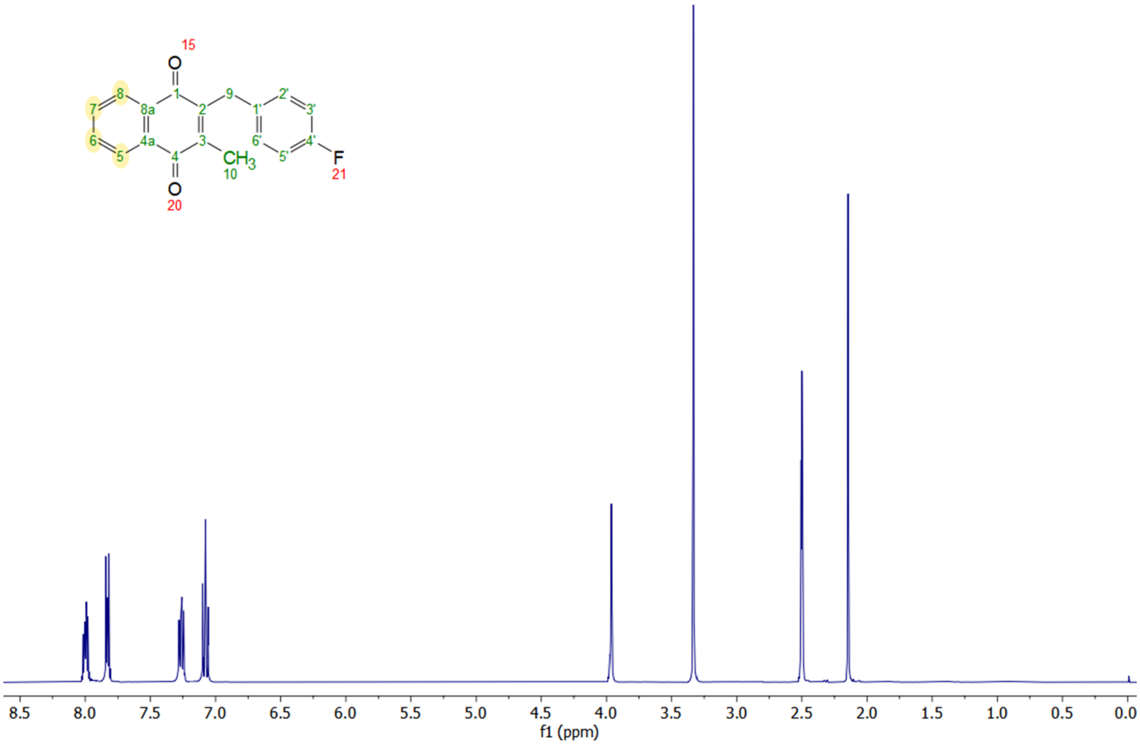

^{13}C NMR

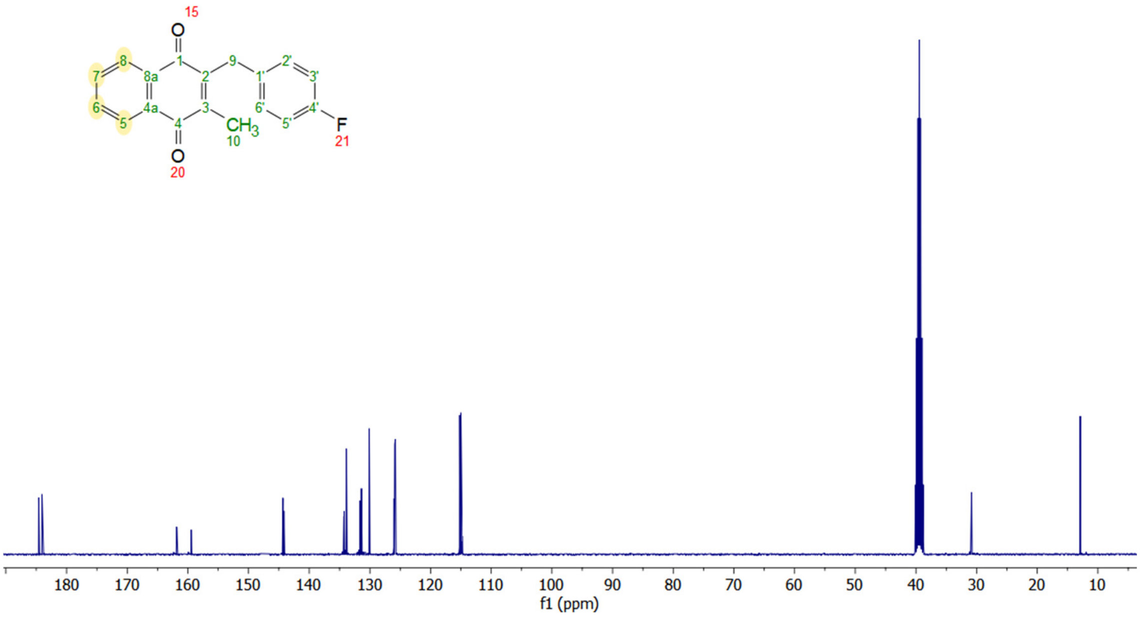

2-Methyl-3-{[4-(trifluoromethoxy)phenyl]methyl}-1,4-naphthoquinone (**2q**)

^{1}H NMR

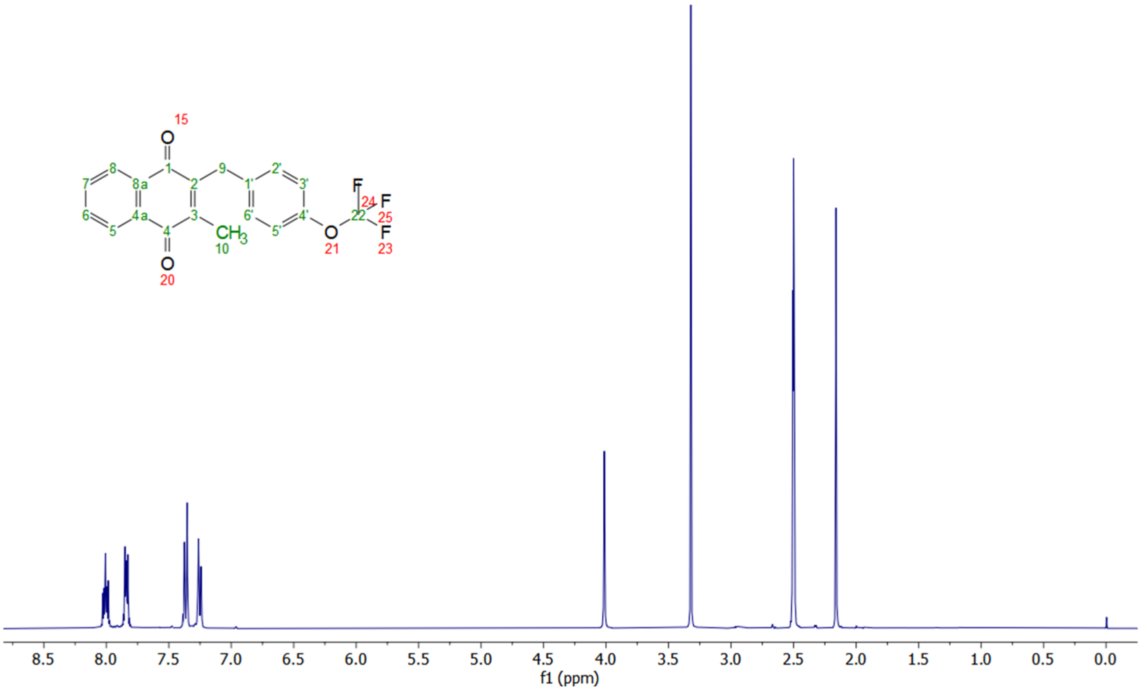

^{13}C NMR

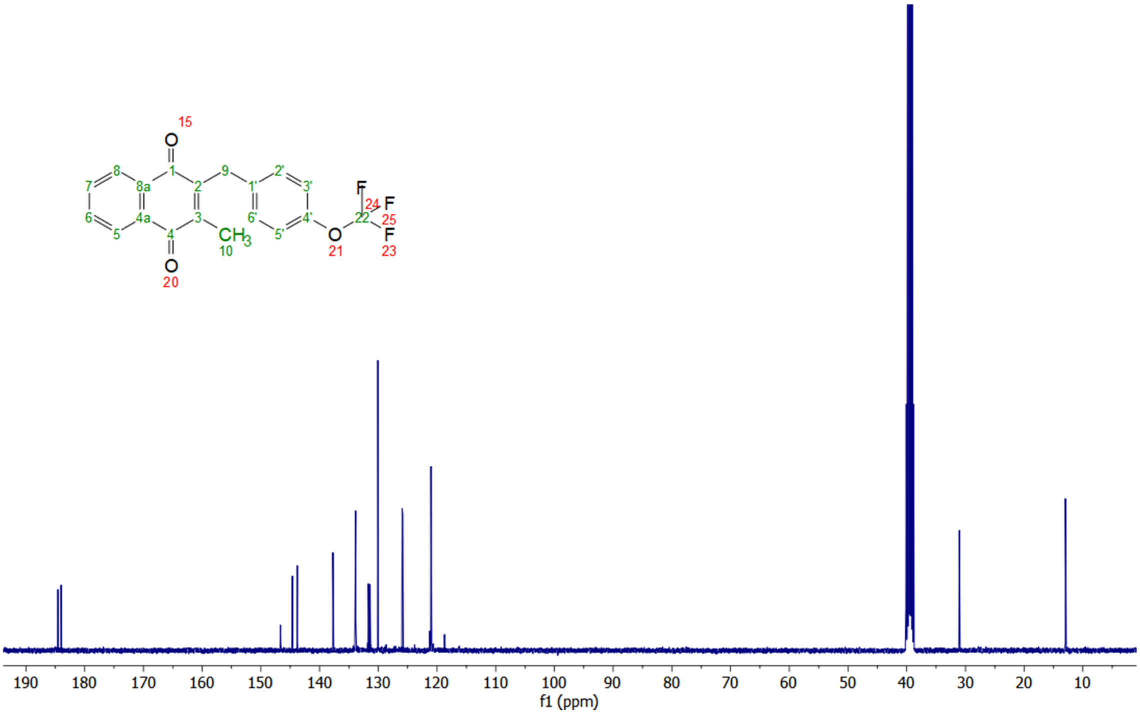

2-Methyl-3-{[3-(trifluoromethoxy)phenyl]methyl}-1,4-naphthoquinone (**2r**)

^{1}H NMR

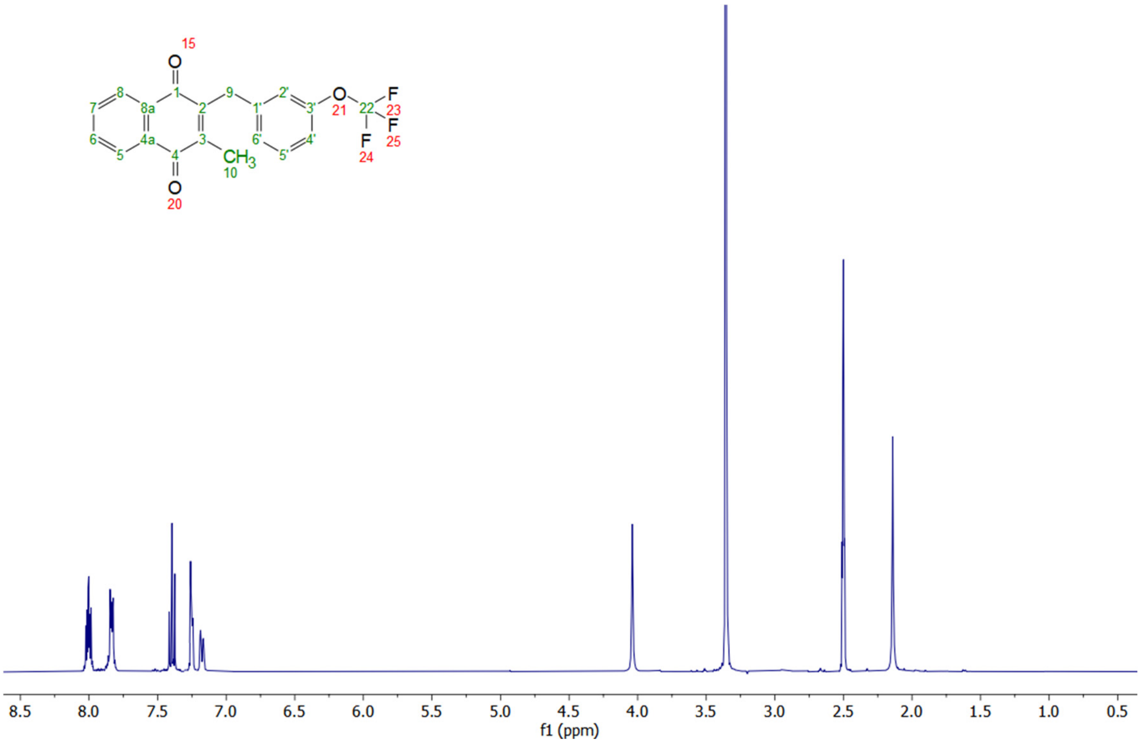

^{13}C NMR

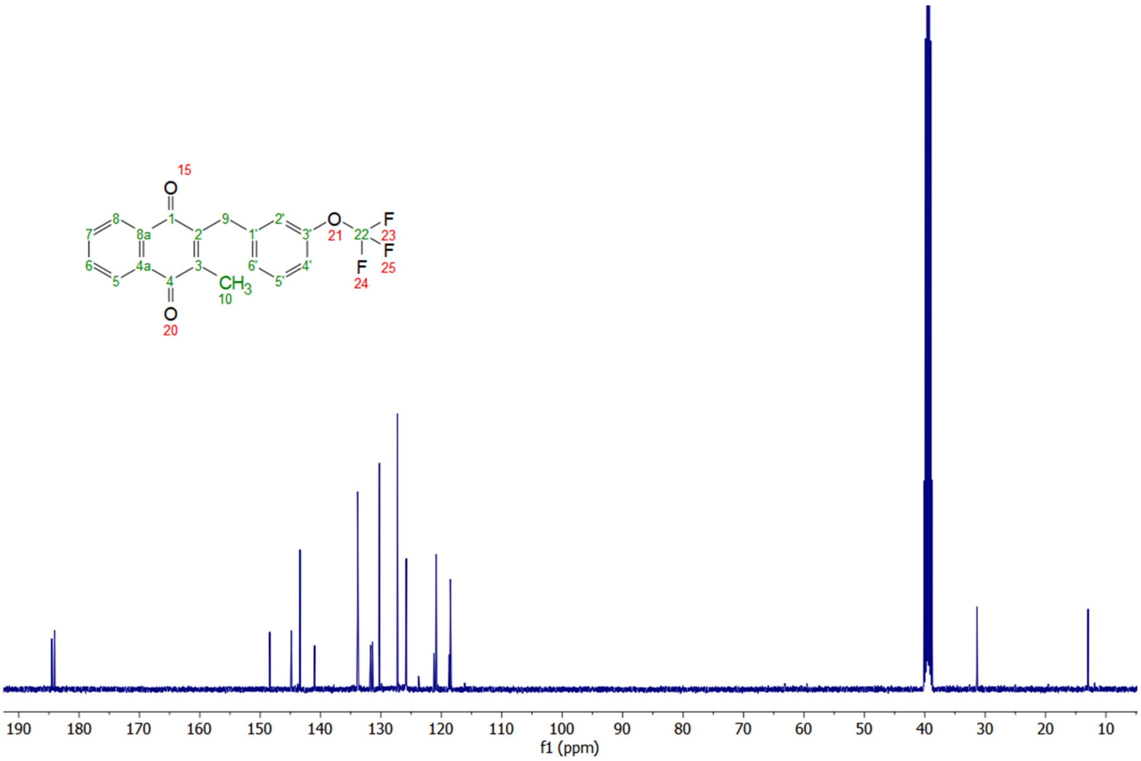

2-{[2-Fluoro-5-(trifluoromethoxy)phenyl]methyl}-3-methyl-1,4-naphthoquinone (**2s**)

^{1}H NMR

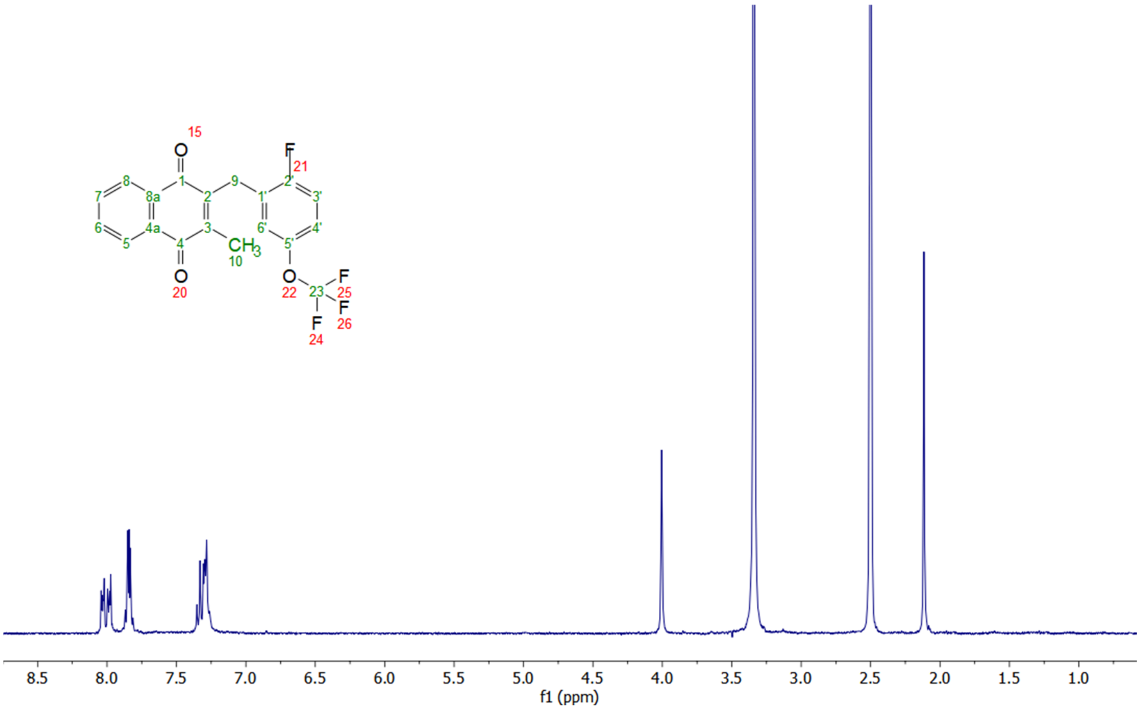

^{13}C NMR

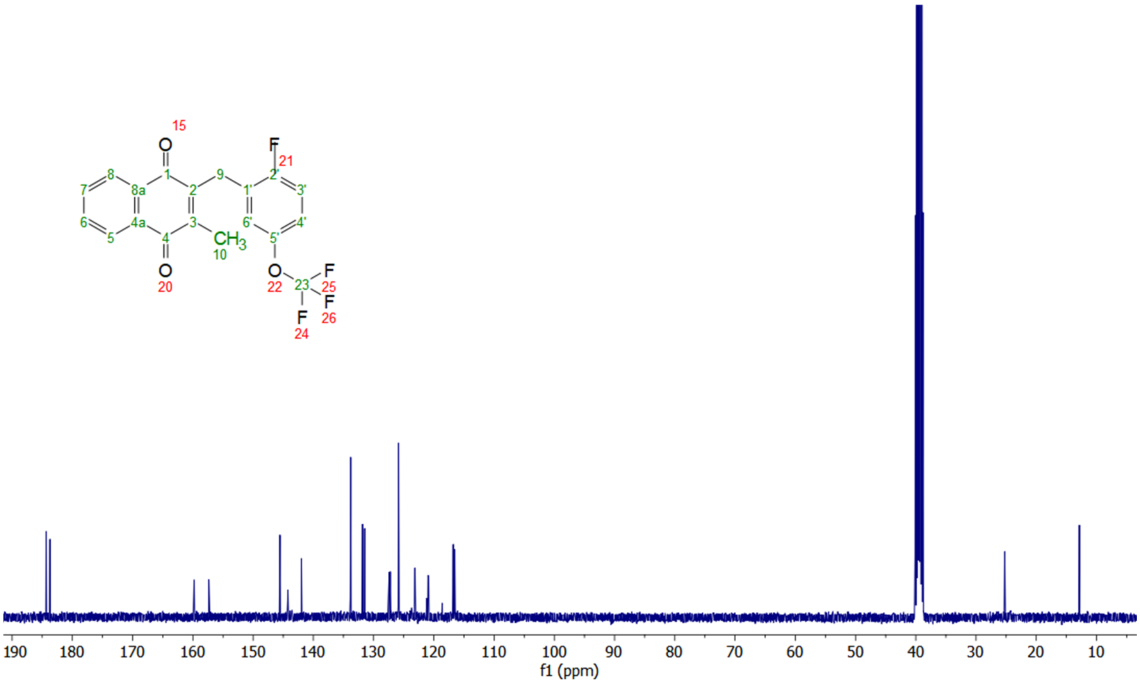

2-Benzyl-1,4-naphthoquinone (**4a**)

^{1}H NMR

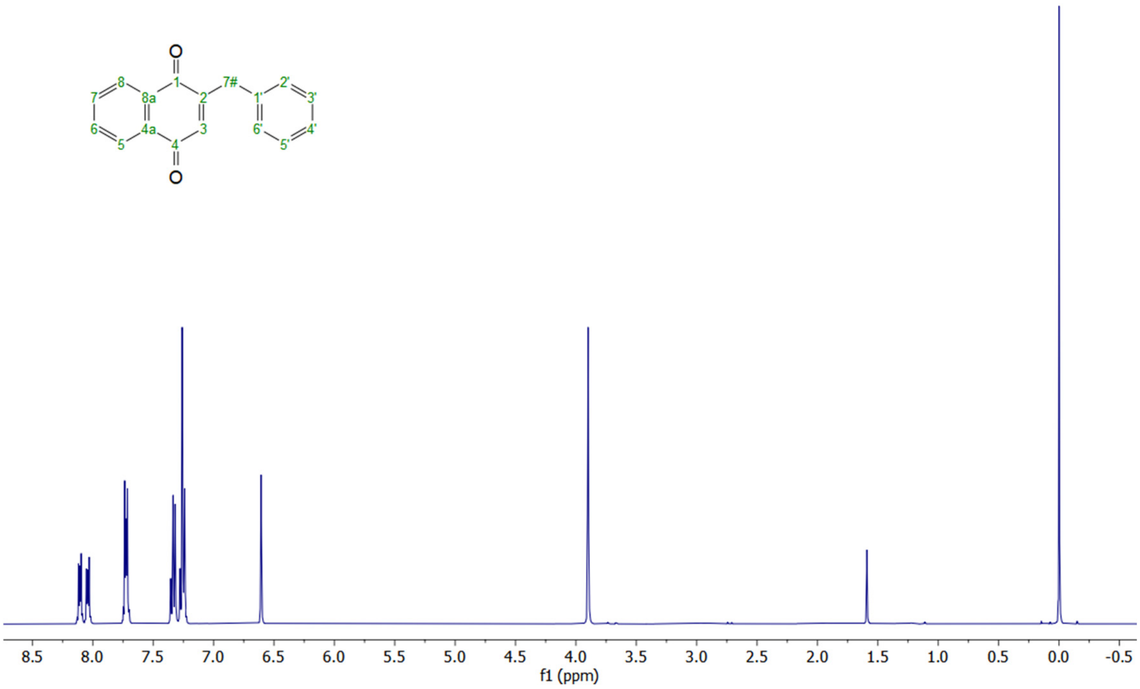

^{13}C NMR

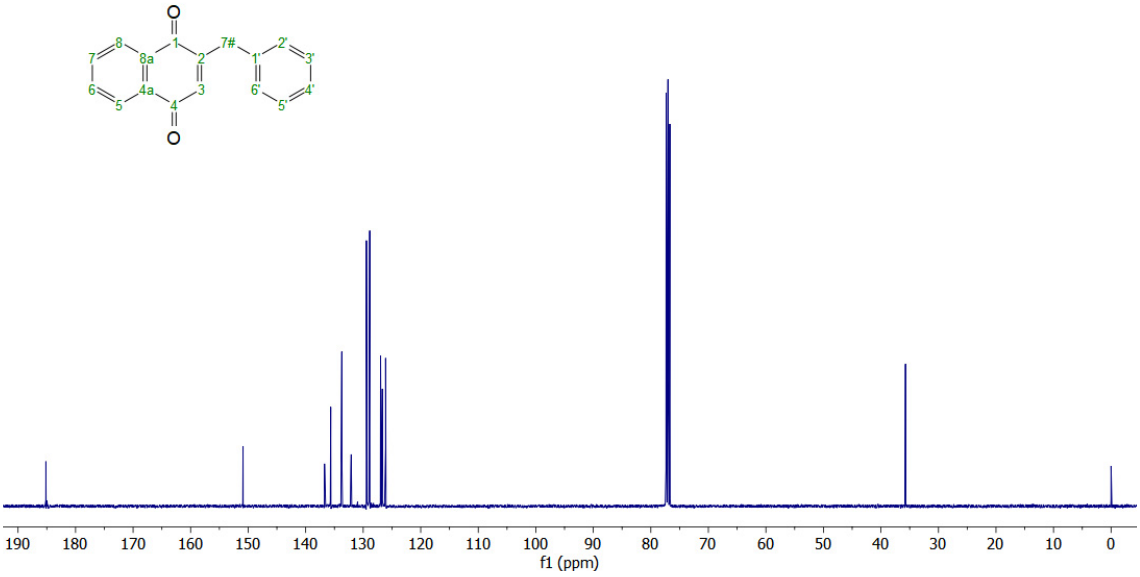

2-{[4-(Trifluoromethyl)phenyl]methyl}-1,4-naphthoquinone (**4b**)

^{1}H NMR

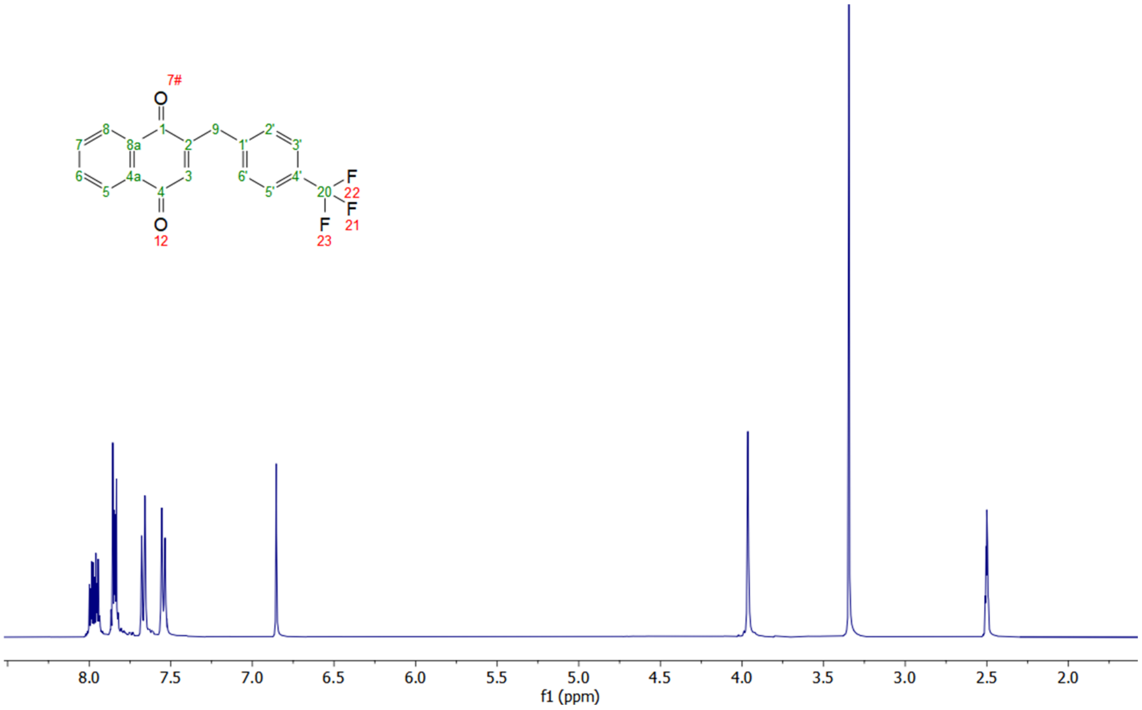

^{13}C NMR

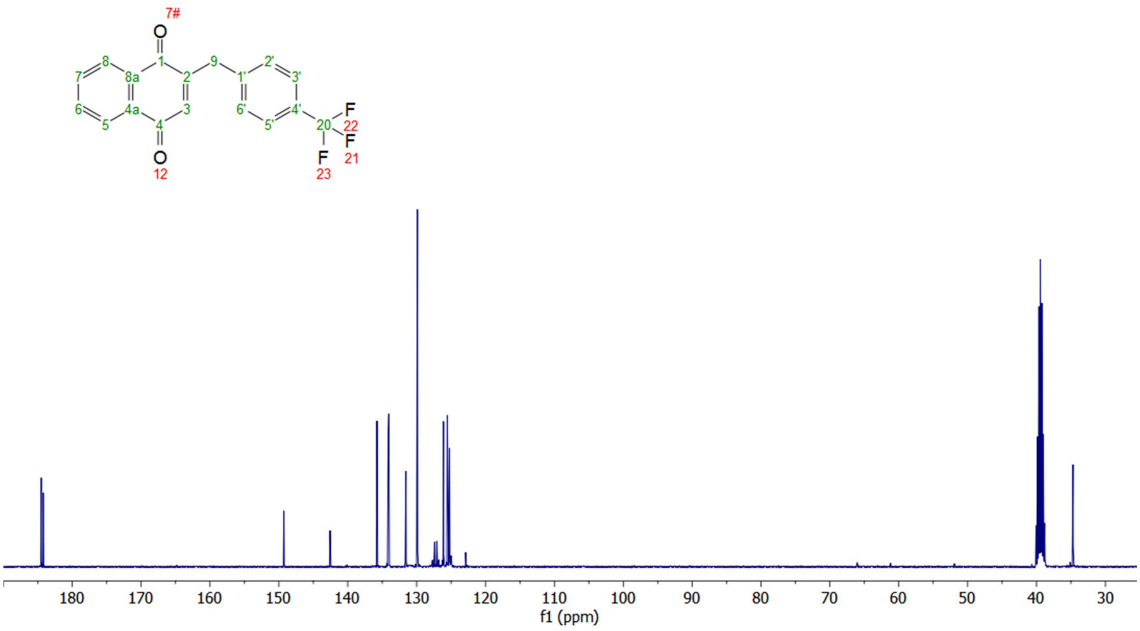

2-{[2-Fluoro-4-(trifluoromethyl)phenyl]methyl}-1,4-naphthoquinone (**4c**)

^{1}H NMR

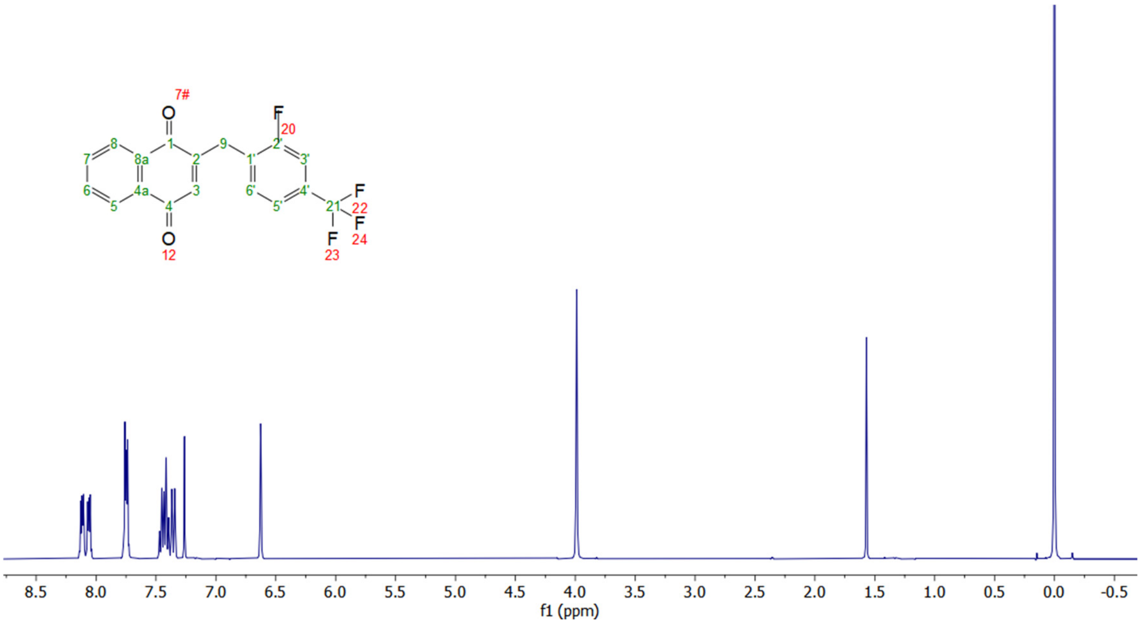

^{13}C NMR

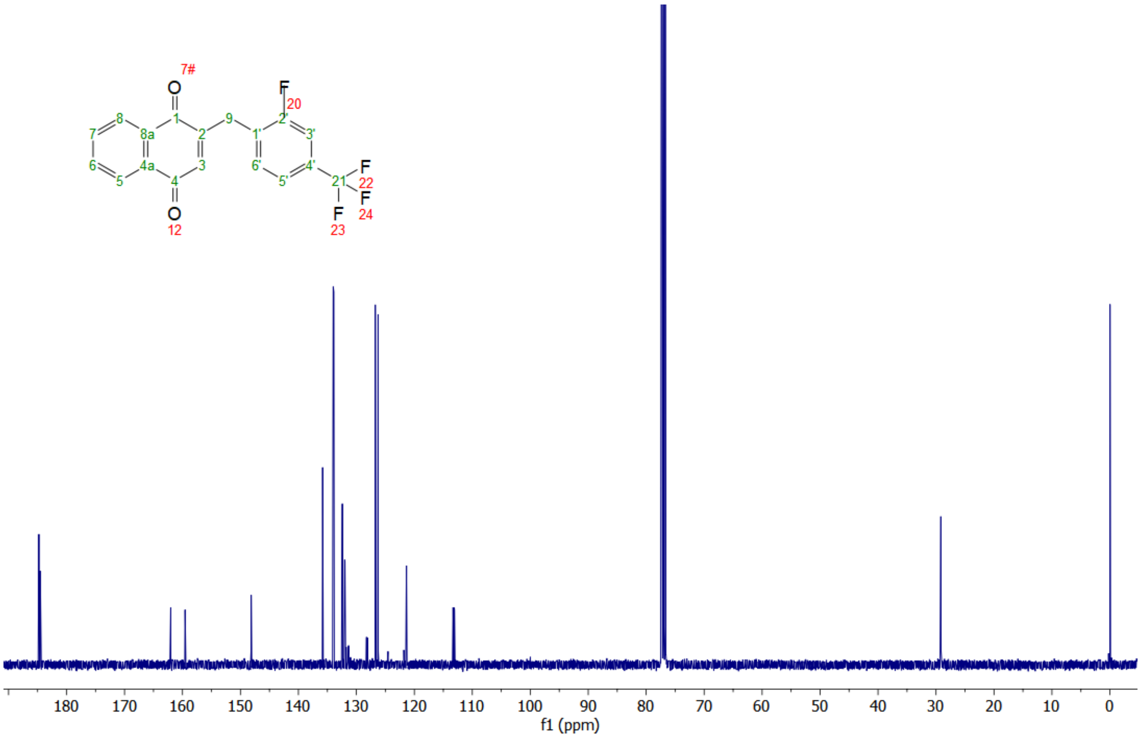

2-{[4-Fluoro-2-(trifluoromethyl)phenyl]methyl}-1,4-naphthoquinone (**4d**)

^{1}H NMR

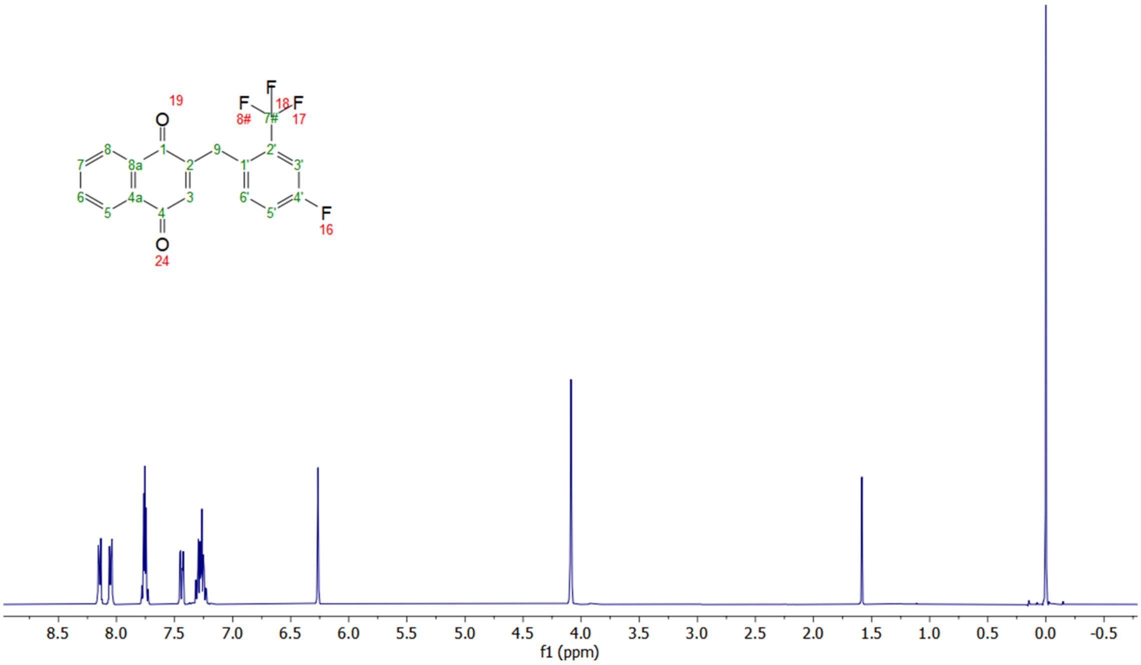

^{13}C NMR

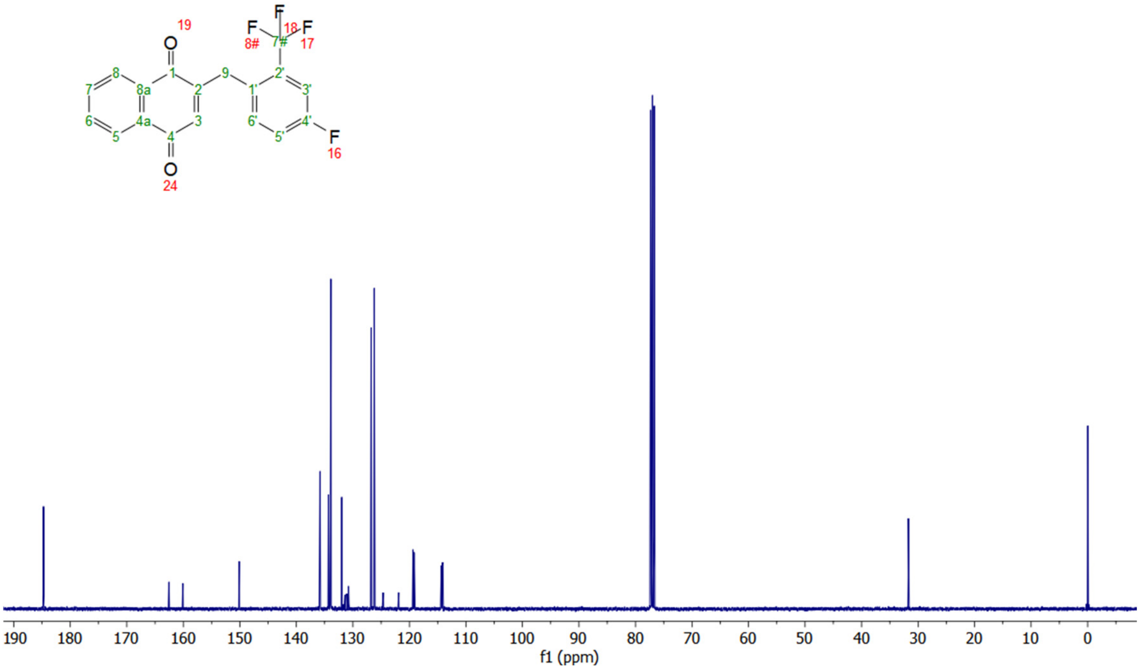

2-[(6-Chloropyridin-3-yl)methyl]-1,4-naphthoquinone (**4e**)

^{1}H NMR

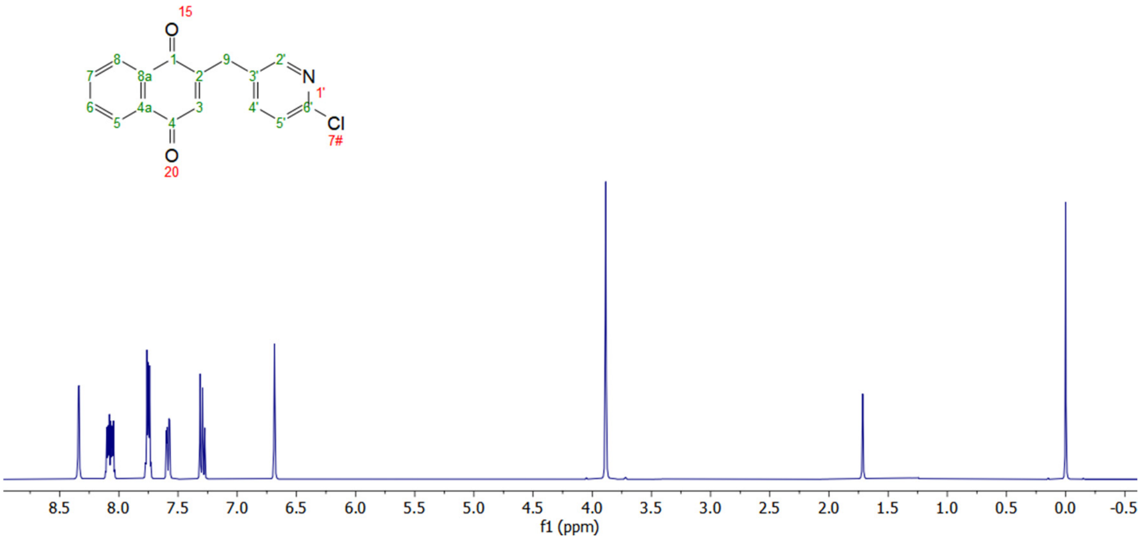

^{13}C NMR

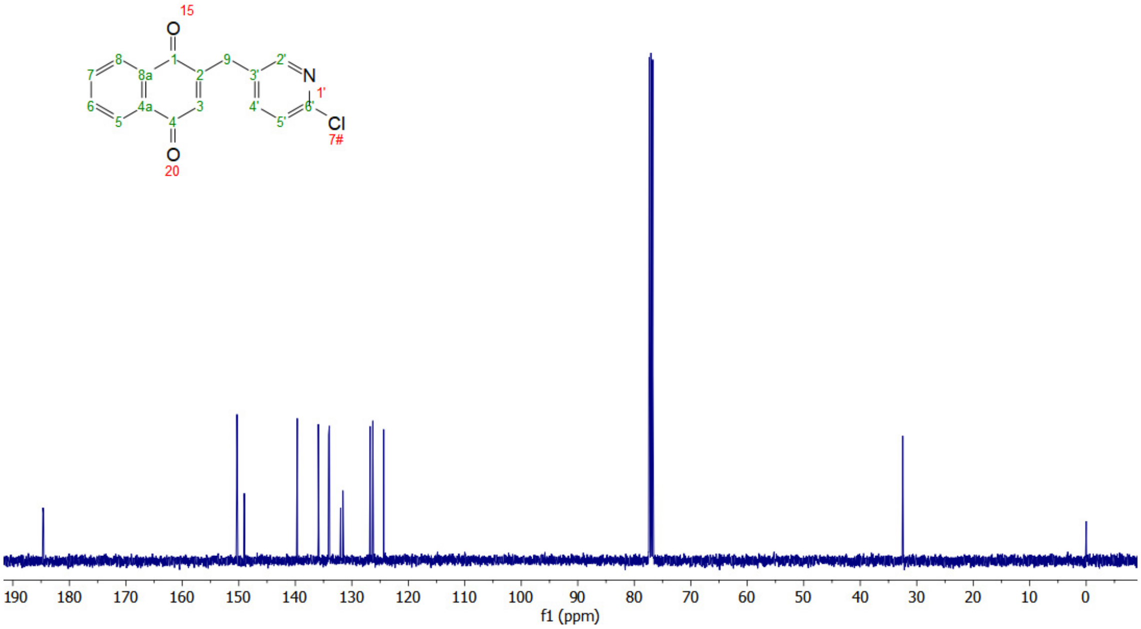

2-Benzyl-3-(trifluoromethyl)-1,4-naphthoquinone (**5a**)

^{1}H NMR

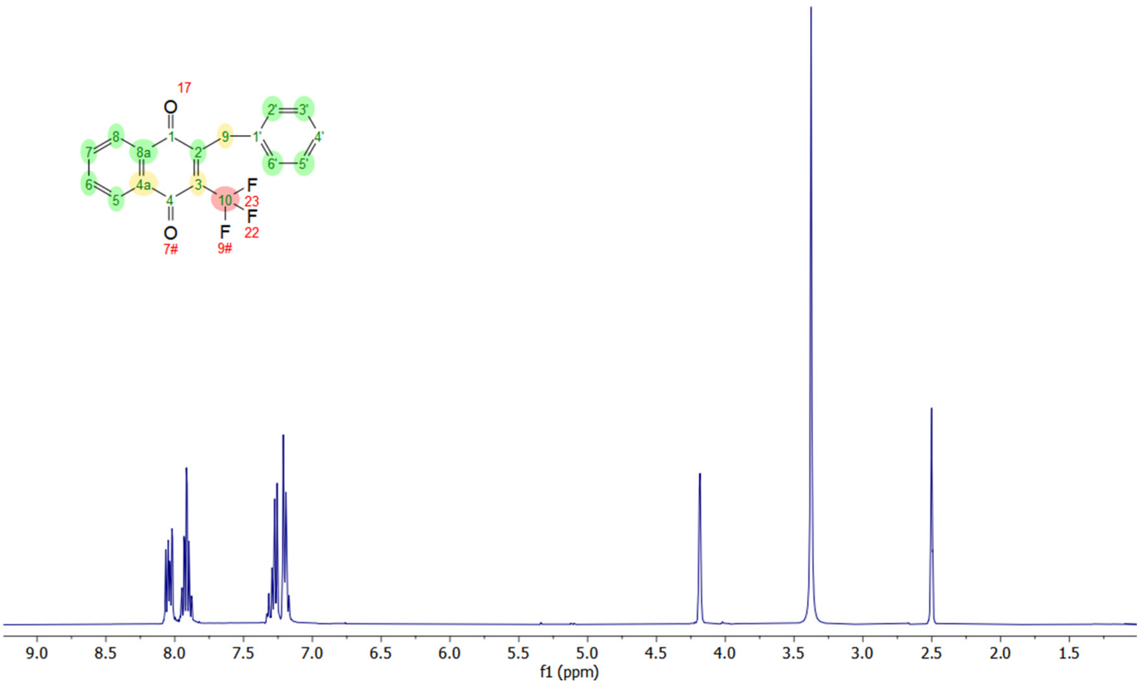

^{13}C NMR

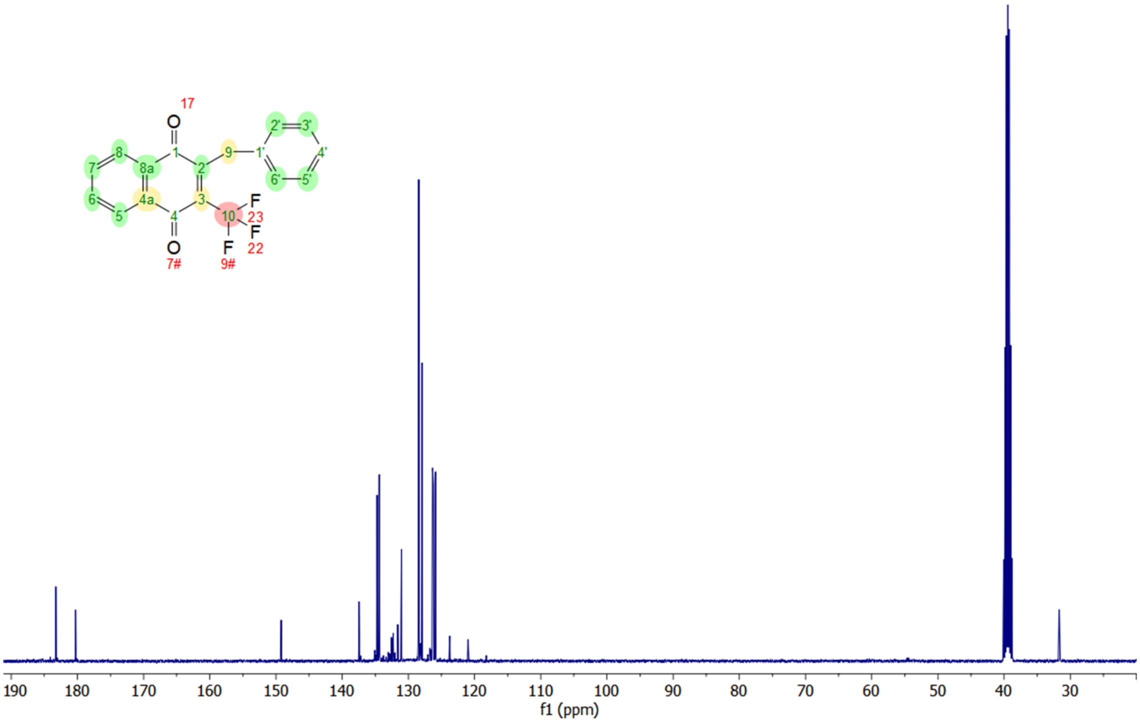

2-(Trifluoromethyl)-3-{[4-(trifluoromethyl)phenyl]methyl}-1,4-naphthoquinone (**5b**)

^{1}H NMR

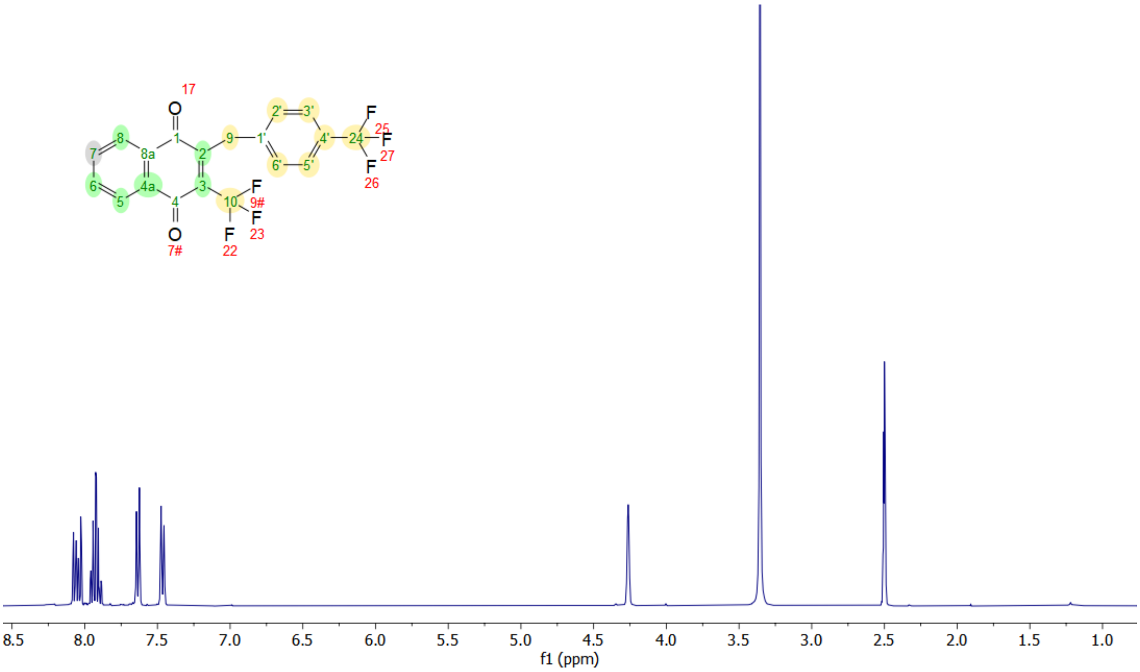

^{13}C NMR

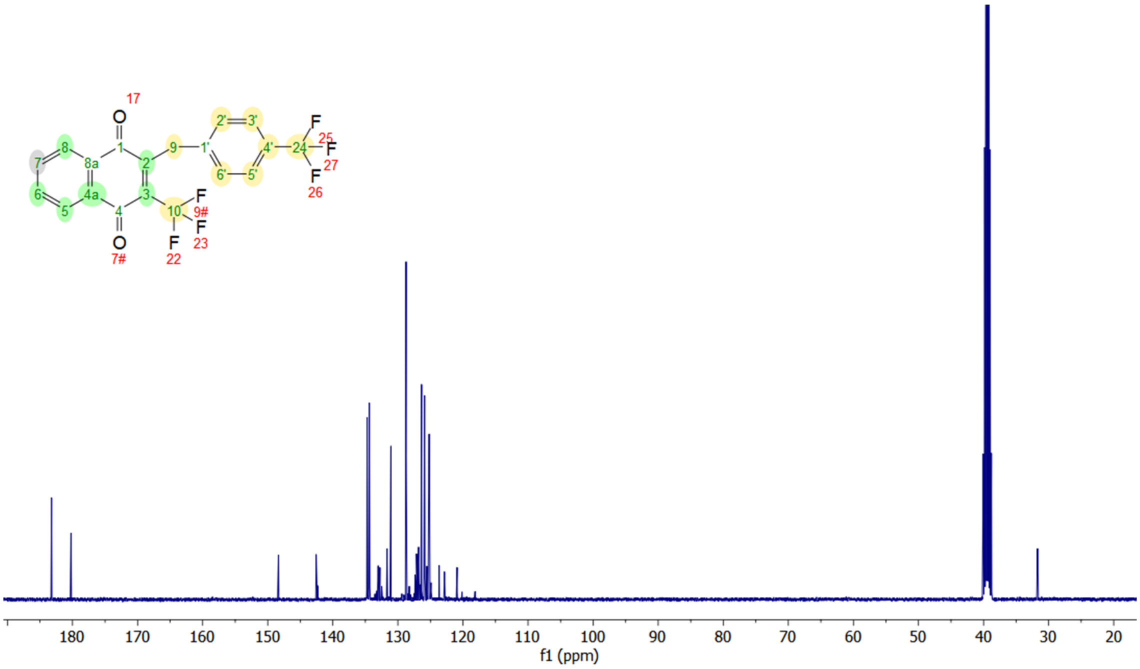

2-{[2-Fluoro-4-(trifluoromethyl)phenyl]methyl}-3-(trifluoromethyl)-1,4-naphthoquinone (**5c**)

^{1}H NMR

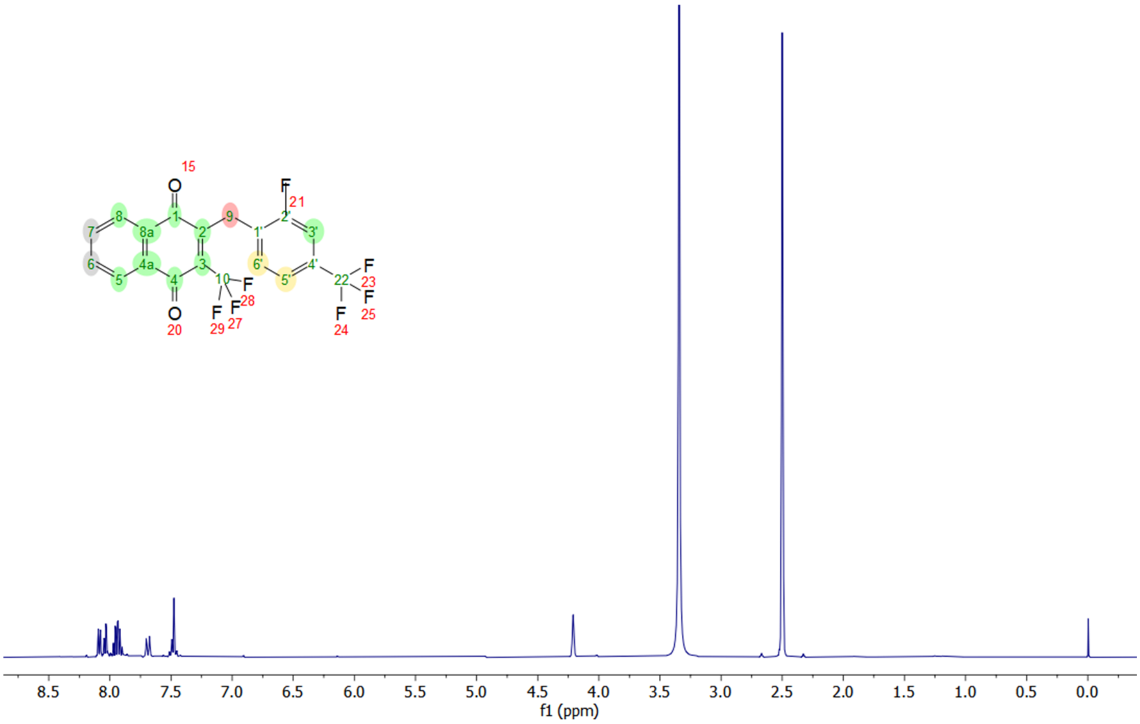

^{13}C NMR

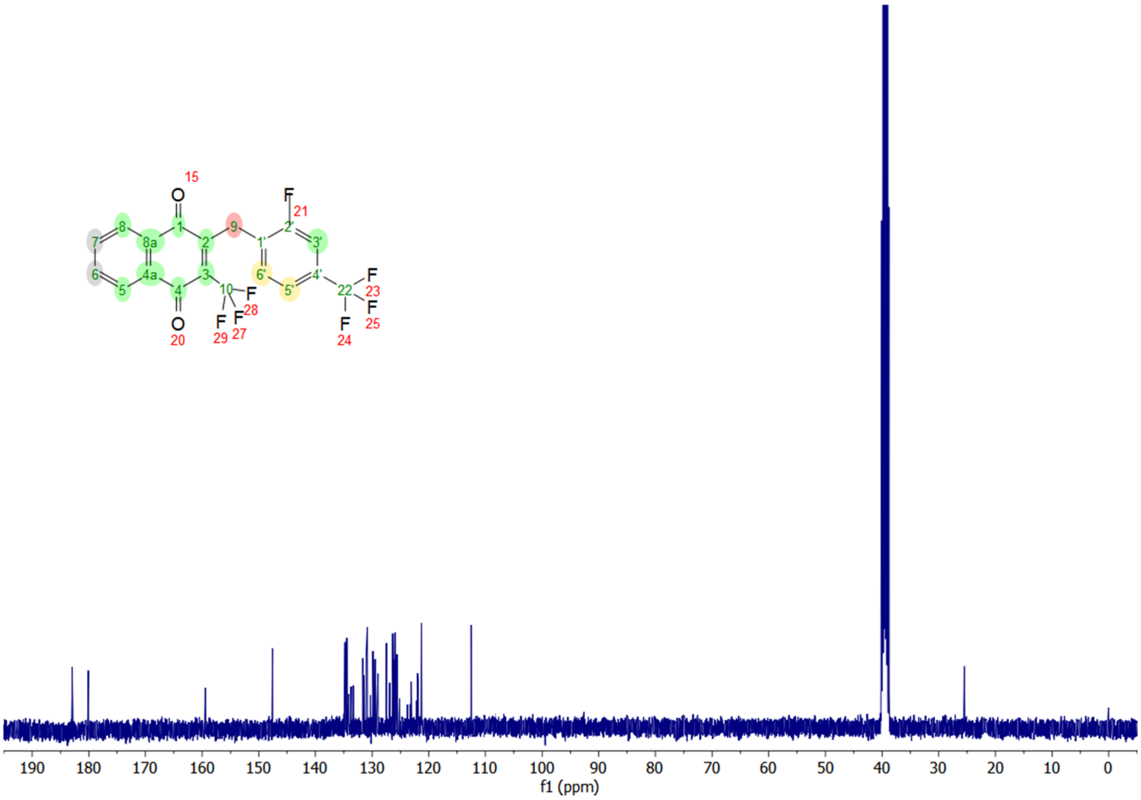

Supplement: Supplementary file 1 [file ijms-26-02114-s001.zip › ijms-3481667-supplementary.pdf]
